# Self‐Assembled Pd(II) Nano‐Adsorbents for Iodine and Methyl Iodide Capture in Vapor and Aqueous Phases

**DOI:** 10.1002/smll.202504242

**Published:** 2025-06-29

**Authors:** Monotosh Dalapati, Raghunath Singha, Pankaj Maity, Debashree Manna, Dipak Samanta

**Affiliations:** ^1^ School of Chemical Sciences National Institute of Science Education and Research (NISER) Bhubaneswar An OCC of Homi Bhabha National Institute (HBNI) PO Bhimpur‐ Padanpur, Via Jatni Khordha Odisha 752050 India; ^2^ Institute of Organic Chemistry and Biochemistry Czech Academy of Sciences v.v.i., Flemingovo nám. 2 Prague 6 Praha 16610 Czech Republic; ^3^ Center for Interdisciplinary Sciences (CIS) National Institute of Science Education and Research (NISER) An OCC of Homi Bhabha National Institute (HBNI) PO Bhimpur‐ Padanpur, Via Jatni Khordha Odisha 752050 India

**Keywords:** coordination cages, dynamic adsorption, iodine and methyl iodide, nonporous, water purification

## Abstract

The excessive release of iodine from industrial and medical activities has led to severe contamination of air and water, causing major concern to public health. Effective capture and secure storage of radioactive iodine in both vapor and aqueous phases are crucial for nuclear safety and ecological protection. In this study, it is explore four non‐porous metal‐organic coordination cages (**MC1**–**MC4**) comprising of Pd‐acceptors and diverse aromatic ligands for iodine adsorption across different media. These cages exhibit remarkable iodine uptake, reaching 3.38 g g⁻¹ in the vapor phase at 75 °C and ≈2.73 g g⁻¹ in aqueous solution, with significantly faster adsorption kinetics than covalent organic framework (COF)‐ and metal‐organic framework (MOF)‐based materials. Moreover, high adsorption capacities are observed in dynamic flow‐through experiments, with **MC2** achieving an elution volume of up to 7.8 L g⁻¹ in a 0.5 mM I₃⁻ solution. Practical tests confirm their efficiency in removing iodine from seawater and drinking water, reducing 5 ppm concentrations to ppb levels. Additionally, the cages exhibit outstanding adsorption of methyl iodide vapor, achieving uptake capacities as high as 0.94 g g⁻¹ under ambient conditions. With high stability, recyclability, and scalable synthesis, these metal‐organic cages emerge as promising nano‐adsorbents for iodine and methyl iodide removal from various environmental matrices.

## Graphical abstract

Industrial and medical activities have led to severe iodine contamination, posing risks to public health. This study investigates four non‐porous self‐assembled coordination cages for efficient iodine capture in vapor and aqueous phases. These cages demonstrate exceptional adsorption capacity, rapid kinetics, and recyclability, making them promising candidates for environmental iodine removal and nuclear safety applications.

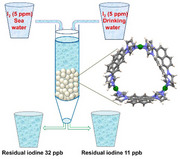

## 1 Introduction

As the world seeks sustainable alternatives to fossil fuels, nuclear energy has emerged as a viable clean energy source, offering a significant reduction in greenhouse gas emissions. Currently, nuclear power contributes ≈10% of global energy production, with projections indicating an increase to 12% by 2050.^[^ ^1^, ^2^ ^]^ However, despite its advantages, the widespread adoption of nuclear energy presents significant challenges, particularly in the safe disposal of radioactive waste.^[^ ^3^ ^]^ During the reprocessing of spent nuclear fuel, several hazardous radionuclides are inevitably released, including isotopes such as ^129^I, ^131^I, ^3^H, ^14^C, ^85^Kr, ^90^Sr, and ^137^Cs, alongside radioactive elements like ^235^U and ^99^Tc.^[^ ^4^ ^]^ Among these, the volatile iodine isotopes ^129^I and ^131^I present the most severe risks. With an extensive half‐life of ≈1.6 × 10⁷ years, ^129^I is highly toxic, highly mobile in environmental systems, and prone to bioaccumulation. In contrast, ^131^I, with a much shorter half‐life of eight days, emits intense radiation that disrupts metabolic processes.^[^ ^5^, ^6^ ^]^ Additionally, substantial amounts of radioactive iodine are discharged into water bodies from nuclear reactor cooling systems, contaminating aquatic ecosystems and posing serious health risks.^[^ ^7^, ^8^, ^9^, ^10^, ^11^ ^]^

Beyond nuclear contamination, ensuring access to clean water remains a fundamental global challenge.^[^ ^12^, ^13^, ^14^ ^]^ Iodine is frequently used as an effective and inexpensive antimicrobial agent for water disinfection, with applications such as the I_2_/KI–based purification system aboard the International Space Station.^[^ ^14^, ^15^ ^]^ However, residual iodine in drinking water must be removed to prevent adverse metabolic effects and unpleasant taste.^[^ ^16^, ^17^ ^]^ Iodine is readily absorbed by human tissues, with the thyroid gland demonstrating the highest retention efficiency. Prolonged internal exposure to radioactive iodine significantly increases the risk of thyroid‐related diseases, including hypothyroidism and thyroid cancer. Furthermore, iodine's strong migration and diffusion properties enable its circulation through environmental compartments–air, water, soil, and living organisms–exacerbating contamination risks.^[^ ^18^ ^]^ Thus, the selective and efficient removal of iodine from gaseous and aqueous waste streams is critical for advancing nuclear energy safety and improving water purification technologies.^[^ ^6^, ^19^ ^]^

Conventional methods for iodine removal from industrial waste streams include wet scrubbing with alkaline solutions (e.g., NaOH or Hg(NO₃)₂) and solid‐phase adsorbents such as silver salt zeolites, activated carbon, and bismuth‐containing minerals.^[^ ^20^, ^21^ ^]^ However, these techniques suffer from limitations such as complex processing, toxic by‐product generation, and low adsorption capacities.^[^ ^9^, ^22^ ^]^ To overcome these challenges, researchers have explored a range of advanced adsorbent materials, including silica,^[^ ^23^ ^]^ activated carbon,^[^ ^24^, ^25^ ^]^ zeolites,^[^ ^26^, ^27^ ^]^ covalent organic frameworks (COFs),^[^ ^28^, ^29^, ^30^ ^]^ metal‐organic frameworks (MOFs),^[^ ^31^, ^32^, ^33^ ^]^ porous organic polymers (POPs),^[^ ^34^, ^35^, ^36^ ^]^ porous organic cages (POCs),^[^ ^37^, ^38^, ^39^ ^]^ and metal‐organic cages (MOCs).^[^ ^40^, ^41^ ^]^ Most studies on iodine capture have focused on adsorption in the solid phase or organic solvents (e.g., *n*‐hexane, cyclohexane), with limited research addressing aqueous‐phase iodine removal due to the poor water stability of many adsorbent materials.^[^ ^7^, ^20^, ^42^ ^]^ Despite these efforts, significant challenges remain in achieving fast kinetics, high adsorption capacities, and selectivity in the presence of competing ions. A particularly pressing issue in nuclear waste management is the efficient removal of low‐concentration iodine (5–16 ppm) from both freshwater and seawater, where contamination levels are significantly lower than those typically studied (>100 ppm). The development of adsorption materials capable of removing iodine at these lower concentrations is crucial for ensuring the safety of drinking water. Recent studies have shown promising results: for example, Ke et al. demonstrated the use of ionic organic frameworks (IOFs) to reduce iodine levels in distilled water from 5 to 0.22 ppm,^[^ ^16^ ^]^ while Van Der Voort et al. employed COFs to achieve a residual iodine concentration of 0.061 ppm in seawater.^[^ ^15^ ^]^ However, despite these advancements, simultaneous remediation of both molecular iodine (I₂) and iodide (I⁻) at trace levels remains an ongoing challenge in the field of environmental remediation and nuclear waste management.^[^ ^43^ ^]^

While extensive research has focused on iodine adsorption, there are relatively few reports on the capture of methyl iodide (CH₃I). Since both I₂ and CH₃I often coexist in off‐gas streams, developing materials capable of simultaneously adsorbing both species remains a challenge. For example, Han et al. synthesized a pyridine‐based covalent organic framework (COF) that achieved concurrent adsorption of 6.0 g g^−1^ of I₂ and 1.45 g g^−1^ of CH₃I.^[^ ^44^ ^]^ In a subsequent study, they developed a triazine‐based COF that showed enhanced adsorption capacities of 8.61 g g^−1^ for I₂ and 1.53 g g^−1^ for CH₃I.^[^ ^29^ ^]^ Similarly, Guo and co‐workers reported cross‐linked polymers that effectively captured both I₂ and CH₃I under similar conditions.^[^ ^45^, ^46^ ^]^ Most reported adsorbents are crystalline and porous materials, but non‐porous solids have long been considered ineffective for such applications.

Coordination cages offer unique structural features, including both internal (endo) cavities and external (exo) binding sites, such as metallo‐rings on their outer surfaces.^[^ ^47^ ^]^ This dual‐cavity architecture has been explored for various applications, including drug delivery,^[^ ^18^, ^48^ ^]^ stabilization of reactive intermediates,^[^ ^49^, ^50^ ^]^ selective guest recognition,^[^ ^51^, ^52^ ^]^ catalysis,^[^ ^53^, ^54^ ^]^ and tunable fluorescence properties.^[^ ^55^, ^56^ ^]^ The chemical environment within the cage is defined by the ligand framework, which influences the behaviour and reactivity of encapsulated guest molecules.^[^ ^57^ ^]^ Cages are typically formed from square‐planar Pd(II) centres and bi‐ or tri‐dentate pyridyl‐based organic ligands, allowing for precise control over cage size and functionality through careful ligand design and reaction conditions.

Iodine uptake is facilitated by strong interactions between electron‐deficient iodine species and electron‐rich binding sites within conjugated frameworks or heteroatom‐containing materials (e.g., nitrogen and oxygen). In contrast, CH₃I capture is primarily driven by N‐methylation at nucleophilic nitrogen sites, leading to the formation of pyridinium and quaternary ammonium salts.^[^ ^29^ ^]^ Developing a single material capable of simultaneously adsorbing I₂ vapor, aqueous I₃⁻, and CH₃I remains a complex challenge.

In this context, Pd(II)‐based coordination cages represent a highly promising platform, combining scalable, high‐yield synthesis with structurally tunable and flexible cavities, strong metal‐mediated binding interactions, and excellent solution processability. Cationic cages constructed with electron‐rich aromatic cores, and nitrogen‐donor ligands such as pyridine and triazole offer a compelling strategy for the simultaneous capture of iodine and methyl iodide, as their π‐rich frameworks and nucleophilic nitrogen centers—featuring both internal (endo) and external (exo) binding sites—create a chemically favorable environment for the efficient adsorption of I₂, I₃⁻, and CH₃I.

In this study, we synthesized four metal‐organic coordination cages (**MC1**, **MC2**, **MC3**, and **MC4**) by the self‐assembly of square‐planar Pd(II) centres with a series of ditopic ligands (**L1**, **L2**, **L3**, and **L4**). These ligands feature electron‐rich aromatic rings and nucleophilic nitrogen sites, creating highly efficient binding sites for radioactive iodine species. The resulting nonporous solid cages exhibited impressive dual adsorption capacities, with **MC3** achieving iodine uptake of up to 3.38 g g^−1^ and **MC1** capturing 1.01 g g^−1^ of methyl iodide at 75 °C, primarily driven by electron‐pair interactions.

Insoluble cages (**MC1**, **MC2**, and **MC3**) also demonstrated remarkable iodine adsorption from aqueous solutions, with **MC2** reaching capacities of 2.73 g g^−1^ for I₂ and 2.26 g g^−1^ for I₃⁻–the first report of iodine uptake by self‐assembled metal‐organic cages in aqueous media. Notably, **MC2** maintained high selectivity for iodine even in the presence of a 100‐fold excess of competing anions such as F⁻, Cl⁻, Br⁻, NO₃⁻, and SO₄^2^⁻, highlighting its potential for practical water purification. The adsorption kinetics was exceptionally fast, with **MC2** removing 96.83% of iodine within 60 sec outperforming previously reported polymeric, COF, and MOF–based materials.

In dynamic flow‐through experiments using 5 ppm iodine solutions, the cages achieved near‐complete iodine removal, reducing iodine concentrations to the low ppb range (as low as 28 ppb for seawater and 11 ppb for drinking water). **MC2** demonstrated particularly high dynamic adsorption capacity, with elution volumes exceeding 7.8 L g^−1^ for a 0.5 mM I₃⁻ solution. Furthermore, the adsorbed iodine could be efficiently recovered through thermal treatment and organic solvent extraction, confirming the material's recyclability and potential for long‐term use in iodine remediation.

## 2 Results and Discussion

### 2.1 Synthesis and Characterization

The ligands L1 and L2, featuring pyridine and naphthalene cores, were synthesized via Ullmann coupling reactions between imidazole and the respective dihaloaromatic precursors, 2,6‐dibromopyridine and 2,7‐dibromonaphthalene (Schemes S1 and S2, Supporting Information) and characterized using NMR and mass spectrometry (Figures S1–S4, Supporting Information).

**Scheme 1:**
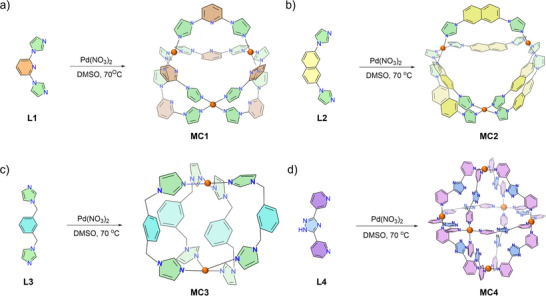
Two‐component self‐assembly of Pd(II) cations with ligands **L1**, **L2**, **L3**, and **L4**, resulting in the formation of cages: a) **MC1**, b) **MC2**, c) **MC3**, and (d) **MC4**.

**Figure 1:**
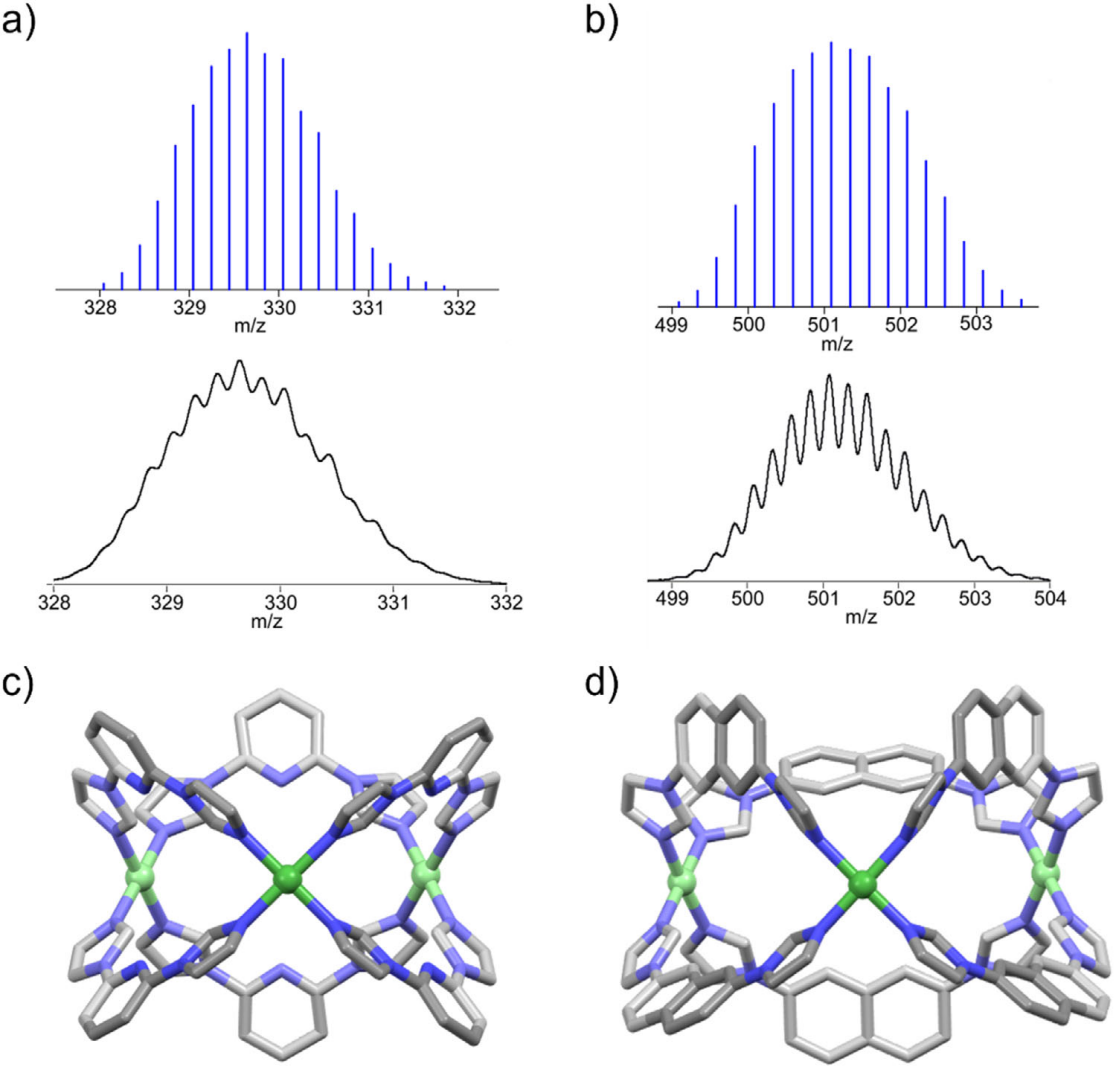
Experimental (blue) and theoretical (black) isotopic patterns of a) [**MC1** + 1NO₃]⁵⁺, and b) [**MC2** + 2NO₃]⁴⁺ fragments from ESI‐MS analysis. Single‐crystal X‐ray structures of c) **MC1**, and d) **MC2**. Color code: carbon (grey), nitrogen (blue), and palladium (green). Hydrogen atoms, counter‐anions, and solvent molecules are excluded for clarity.

Two‐component self‐assembled structures (**MC1** and **MC2**) were prepared by combining Pd(NO_3_)_2_ with ditopic ligands (**L1** and **L2**) in DMSO, maintaining molar ratios of 1:2. The reaction mixtures were stirred at 70 °C for 12 h, leading to the formation of the desired assemblies (**Scheme** **1**). Post‐reaction, the mixtures were centrifuged to obtain a clear solution, followed by precipitation with excess ethyl acetate. The obtained metal‐organic cages (**MC1**, and **MC2**) were characterized using ^1^H NMR, ^13^C NMR and a suite of 2D NMR spectroscopy (Figures S11–S24, Supporting Information). ^1^H NMR spectra of both the complexes exhibited a downfield shift of the imidazole N‐CH‐N proton resonance from 8.63 to 9.81 ppm, and from 8.03 to 9.46 ppm in DMSO‐*d*_6_, for **MC1**, and **MC2**, respectively, which indicates the coordination of the imidazole nitrogen to the palladium canter (Figures S11 and S18, Supporting Information). In addition, DOSY NMR spectra show single band for the complexes with diffusion coefficients D ≈ 7.76 × 10^−11^ m^2^ s^−1^, and 6.76 × 10^−11^ m^2^ s^−1^ for **MC1**, and **MC2**, respectively, representing the formation of a single self‐assembled complex (Figures S16 and S23, Supporting Information). The composition of the coordination complexes was confirmed from the ESI‐MS analysis of the cages which displayed several prominent peaks at m/z = 590.0657 and 329.6454 corresponding to the fragments [**MC1**+3(NO_3_)]^3+^ and [**MC1**+1(NO_3_)]^5+^, respectively, and 1064.1582, 688.1104, and 501.0912 corresponding to the fragments [**MC2**+4(NO_3_)]^2+^, [**MC2**+3(NO_3_)]^3+^, and [**MC2**+2(NO_3_)]^4+^, respectively (Figures S17 and S24, Supporting Information). In addition, the experimental isotopic distribution patterns for the fragments [**MC1**+1NO_3_]^5+^, [**MC2**+4(NO_3_)]^2+^, and [**MC2**+2(NO_3_)]^4+^, closely matched with theoretical value (**Figure** **1**a,b; Figure S24, Supporting Information).

Finally, the structure of **MC1**, and **MC2** were confirmed by single‐crystal X‐ray diffraction analysis (Figure 1c,d; Tables S1 and S2, Supporting Information). Single crystals were obtained through gradual vapor diffusion of THF and 1,4‐dioxane into a DMSO solution of the complexes **MC1** and **MC2**, respectively, over two weeks. X‐ray diffraction analysis revealed that **MC1** crystallizes in the hexagonal space group *P*6_3_/*mmc* with the asymmetric unit containing one Pd(II) ion and half of the ligand (Figure 1c). The pyridine nitrogen of the ligand **L1** points toward the cavity, adopting a trifacial barrel‐like conformation, where Pd(II) metal canters occupy the vertices, with an intermetallic distance of 8.57 Å.

Similarly, complex **MC2** crystallized in the orthorhombic, specially *Pnma* space group, adopting an idealized *D*_3_h symmetry (Figure 1d). The asymmetric unit comprises one and a half palladium and two independent ligands and two half of ligands. Each palladium centre adopts a nearly square planar coordination geometry, with average Pd‐N bond lengths ranging from 1.98 to 2.00 Å. The magnetic environments surrounding all Pd(II) ions are nearly identical, with a metal‐metal separation of ≈11.68 Å.

Because iodine is electron‐deficient, it readily forms stable charge‐transfer complexes with the π‐electron‐rich aromatic framework of the synthesized cages, **MC1** and **MC2**. To explore the impact of a modified aromatic core, ligand **L3** and the corresponding **MC3** cage were prepared using a reported literature procedure (Scheme S3, Figures S5, S6, and S25–S31, Supporting Information).^[^ ^58^ ^]^ Additionally, the sp^2^‐hybridized nitrogen atoms in the pyridine ring can interact with iodine through electron‐pair donation and react with methyl iodide to form pyridinium salts. Building on this interactions, we synthesized cage **MC4**, which incorporates a triazole ring (Scheme 1d; Figures S32–S37, Supporting Information).^[^ ^59^ ^]^ The higher nitrogen content in **MC4** is expected to enhance its adsorption capacity for both I₂ and CH₃I, thereby broadening the potential of these self‐assembled coordination cages for selective adsorption and capture.

Thermogravimetric analysis (TGA) under a nitrogen atmosphere was conducted to evaluate the thermal stability of the cages. The results showed that all four cages remain stable up to 220 °C, with initial weight loss attributed to solvent removal and decomposition occurs beyond 220 °C (Figure S56, Supporting Information). Nitrogen adsorption‐desorption isotherms at 77 K were measured to assess the porosity of the cages. Before analysis, the as‐prepared powders were degassed at 100 °C under high vacuum for 12 h to remove residual solvents. The Brunauer‐Emmett‐Teller (BET) surface areas of **MC1**, **MC2**, **MC3**, and **MC4** were measured to be 12.92, 12.55, 13.19, and 45.4 m^2^ g^−1^, respectively (Figure S44, Supporting Information), with corresponding pore diameters of ≈1.65, 1.56, 1.85, and 1.56 nm (Figure S45 and Table S3, Supporting Information). These TGA and BET results confirm that the cages are thermally stable and nonporous.

### 2.2 Static Iodine Vapor Adsorption

**Figure 2:**
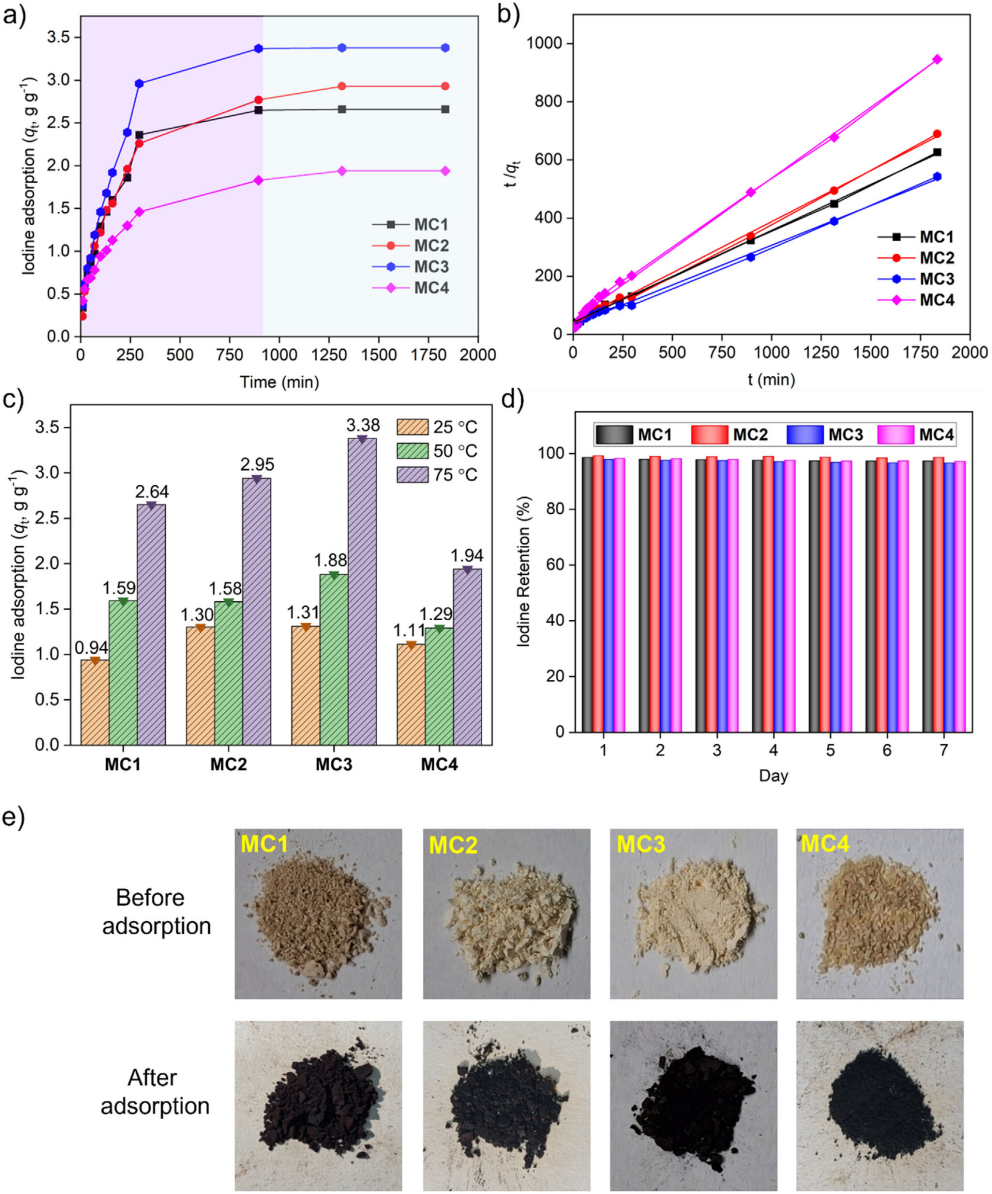
a) Time‐dependent iodine vapor adsorption isotherms at 75 °C. b) Linear fit of pseudo‐second‐order kinetic models for iodine vapor uptake by **MC1**, **MC2**, **MC3**, and **MC4**. c) Comparative static iodine vapor uptake capacities of the cages at different temperatures. d) Iodine retention performance under ambient conditions. e) Photographs showing the color change of each cage upon iodine vapor exposure.

The cationic frameworks of **MC1**, **MC2**, **MC3**, and **MC4**, enriched with nitrogen‐containing heteroatoms and π‐conjugated aromatic units, were anticipated to exhibit a strong affinity for iodine, making them promising candidates for I₂ adsorption. Iodine uptake under static, closed conditions was evaluated by exposing the cages to I₂ vapor at 75 °C—a representative temperature for nuclear fuel reprocessing (Figure S51, Supporting Information). Upon exposure, the adsorbents underwent a noticeable color change from yellowish‐white to dark black over time (Figure 2e). The mass of absorbed iodine increased steadily, with a significant rise during the first 280 min, likely due to the availability of abundant adsorption sites within the cage framework (**Figure** **2**a). After 36 h of exposure, the adsorption capacities for **MC1**, **MC2**, **MC3**, and **MC4** were determined to be 2.65, 2.92, 3.38, and 1.94 g g^−1^, respectively (Figure 2c). To understand the specific influence of nitrogen donor groups, we synthesized a control cage, **MC5**, from a meta‐phenylene‐cored ligand (1,3‐diimidazole benzene) (Scheme S6 and Figures S38–S43, Supporting Information). This allowed us to maintain a cavity size similar to **MC1** but systematically remove the nitrogen donor sites. The observed iodine adsorption capacity of **MC5** was 1.48 g g^−1^, considerably lower than that of **MC1** (Figure S53, Supporting Information). This disparity provides compelling evidence for the powerful contribution of pyridyl nitrogen lone pairs in mediating strong iodine interactions and enhancing adsorption. The adsorption capacity of **MC3** under similar conditions is highest among the reported metal‐organic cages (Table S14, Supporting Information). While **MC4**, with its higher surface area, would typically be expected to show greater adsorption capacity, **MC1**, **MC2**, and **MC3**, despite their lower surface areas, exhibited higher iodine uptake. This suggests that iodine adsorption is not solely dependent on surface area and pore volume, as indicated by N₂ adsorption‐desorption isotherms.^[^ ^9^, ^60^ ^]^ Therefore, surface chemistry and pore architecture likely play a crucial role in controlling the adsorption behaviour of these materials.

These findings indicate that the high iodine uptake capacity of the cages arises from a synergistic interplay of nitrogen donor sites, π‐electron‐rich aromatic scaffolds, and multiple supramolecular non‐covalent interactions—including halogen bonding, π–I, metal–halogen interactions, and van der Waals forces. In addition, electrostatic attraction between polyiodide anions and the positively charged Pd(II) centers contributes significantly to stabilizing the adsorbed iodine species. Powder X‐ray diffraction (PXRD) and field‐emission scanning electron microscopy (FE‐SEM) analyses revealed that **MC3** has a crystalline structure, whereas **MC1**, **MC2**, and **MC4** are amorphous (Figures S46 and S57, Supporting Information). The crystallinity of **MC3** likely contributes to its enhanced I₂ adsorption capacity compared to the amorphous cages. At ambient conditions, the iodine uptake capacities for **MC1**, **MC2**, **MC3**, and **MC4** were measured to be 0.94, 1.3, 1.31, and 1.11 g g^−1^, respectively (Figure S54, Supporting Information). Increasing the temperature further enhanced iodine uptake, likely due to the increased kinetic energy of I₂ molecules, which promotes more frequent and effective interactions with the adsorbent surface (Figure 2c). Additionally, kinetic studies showed that iodine vapor adsorption on these cages followed a pseudo‐second‐order mechanism, with a high correlation coefficient (R^2^ = 0.99), indicating a strong fit to the model (Figure S52 and Table S4, Supporting Information). The pseudo‐second‐order rate constants for **MC1**, **MC2**, **MC3**, and **MC4** were calculated as 0.2046, 0.1452, 0.1332, and 0.2946 g g^−1^ h^−1^, respectively, suggesting that chemisorption is the dominant adsorption mechanism (Figure 2b).^[^ ^61^ ^]^ Among the four cages, **MC4** exhibited the highest rate constant, which can be attributed to its greater porosity and higher sp^2^ nitrogen content, facilitating rapid initial iodine uptake (Figure S52 and Table S4, Supporting Information). The adsorption kinetics of **MC4** are comparable to those reported for various porous materials.

To evaluate the long‐term iodine retention capacity, I₂‐saturated cages were exposed to ambient conditions for 7 days. Minimal weight loss (<5%) was observed over this period, confirming the strong binding affinity between iodine and the cages, and highlighting their excellent retention capabilities (Figure 2d). These results highlight the potential of these metal‐organic cages for the safe transportation and storage of radioactive iodine vapor generated during the reprocessing of spent nuclear fuel.

### 2.3 Mechanistic Insight into Iodine Adsorption

To better understand the iodine adsorption mechanism and the nature of the adsorbed species, a comprehensive set of characterizations was performed, including powder X‐ray diffraction (PXRD), electron paramagnetic resonance (EPR), scanning electron microscopy (SEM), Raman spectroscopy, Fourier‐transform infrared spectroscopy (FTIR), and X‐ray photoelectron spectroscopy (XPS).

After iodine adsorption, PXRD analysis primarily displayed diffraction peaks corresponding to the host materials, with no distinct crystalline peaks of elemental iodine. This suggests that iodine was incorporated into the cage framework in an amorphous state (Figure S57, Supporting Information).

TGA analysis of the iodine‐loaded cages showed substantial weight loss between 40–180 °C, confirming a significant amount of iodine encapsulation (Figure S56, Supporting Information). The degree of weight loss for each cage was directly correlated with its iodine adsorption capacity. Notably, the thermal stability of iodine within the I₂‐loaded cages was significantly higher than that of free iodine, which typically sublimes at 65 °C, indicating strong host‐guest interactions. SEM analysis showed that **MC1**, **MC2**, and **MC4** exhibited amorphous, irregular morphologies with varying particle size distributions, whereas **MC3** displayed well‐defined crystalline block structure (Figure S46, Supporting Information). After iodine adsorption, the cages remained amorphous, but a noticeable increase in surface roughness was observed, giving the materials a fluffier appearance (Figure S62, Supporting Information). The EDAX spectra of the iodine‐loaded cages showed characteristic iodine peaks within the 0.33–0.66 keV range (Figures S63–S66, Supporting Information).^[^ ^62^ ^]^ The intensity of these peaks correlated with the amount of iodine adsorbed by each cage. Additionally, energy‐dispersive X‐ray spectroscopy (EDS) mapping confirmed a uniform and dense distribution of iodine across the cage surfaces (Figures S63–S66, Supporting Information). This even dispersion suggests that the strong interaction between iodine and the cage framework prevents localized crystallization, enhancing the stability and adsorption efficiency of the materials.

**Figure 3:**
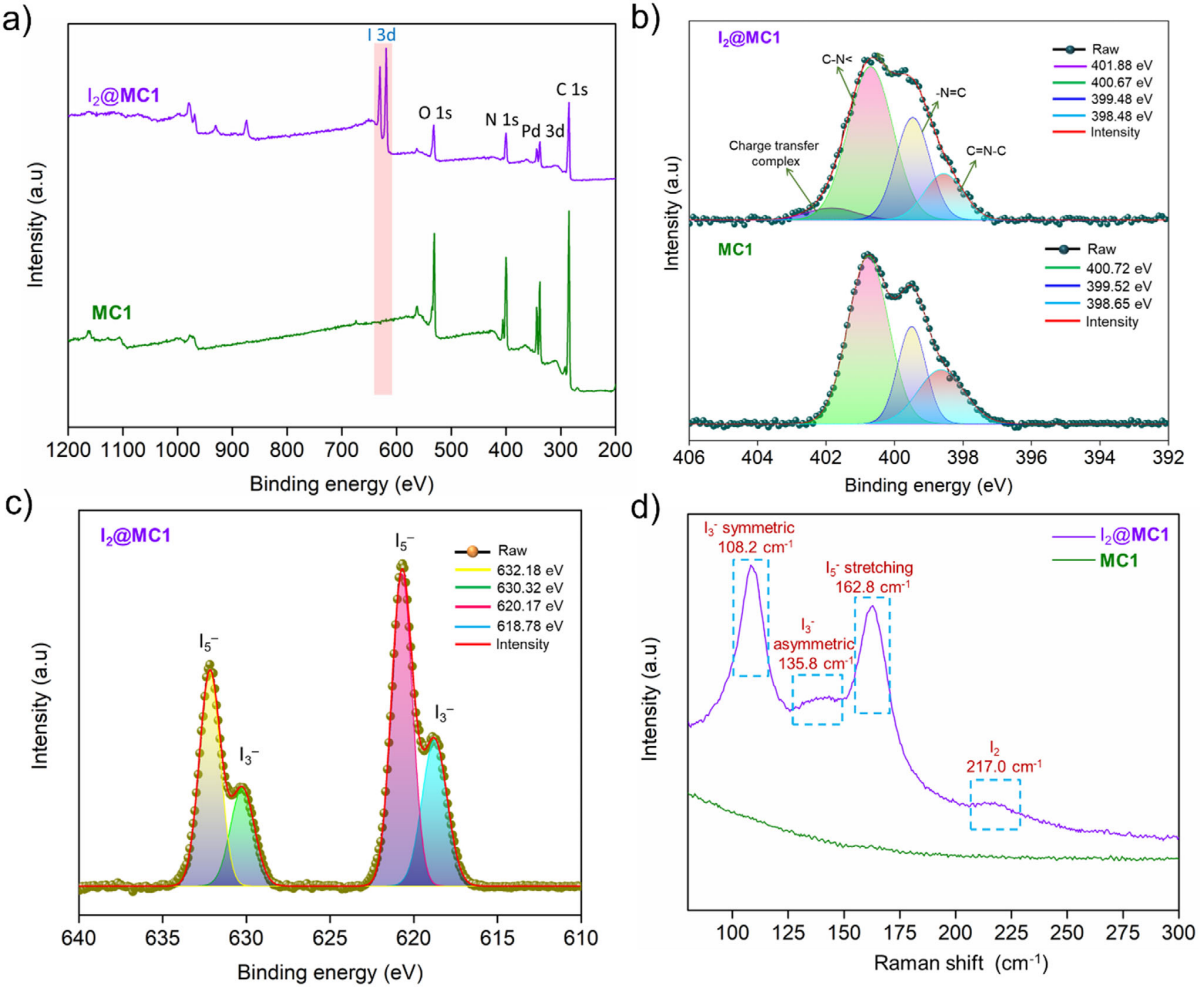
a) XPS spectra of **MC1** before and after iodine adsorption, showing the appearance of an I 3d peak in the 618–630 eV binding energy range after iodine uptake. b) Comparison of N 1s XPS spectra before and after iodine adsorption. c) High‐resolution XPS spectra of the I 3d region for **MC1** after iodine saturation. d) Raman spectra of **MC1** and I₂@**MC1**.

Raman spectroscopy of iodine‐loaded cages revealed distinct peaks characteristic of polyiodide species (I₃⁻ and I₅⁻) that were absent in the pristine cages (**Figure** **3d**; Figures S59–S61, Supporting Information). In particular, I₂@**MC1** exhibited a broad band in the 100–190 cm^−1^ region, including symmetric stretching of I₃⁻ at 108.2 cm^−1^, antisymmetric stretching of I₃⁻ at 135.8 cm^−1^, and the stretching mode of I₅⁻ at 162.8 cm^−1^. A weak and broad peak at 215–220 cm^−1^ was also detected, corresponding to molecular I₂ (Figure 3d).^[^ ^29^, ^63^ ^]^ These results indicate that the adsorbed iodine undergoes substantial conversion to polyiodide, facilitated by the electron‐rich nitrogen sites within the triazole and pyridyl moieties of the ligand core. The nitrogen atoms, with their lone electron pairs, likely engage in Lewis acid‐base interactions with iodine, donating electron density into the antibonding σ* orbital of iodine. This charge transfer promotes further iodine adsorption and the formation of I₃⁻ and I₅⁻ through halogen bonding, suggesting that chemisorption is the primary mechanism driving iodine uptake in the cages.^[^ ^11^, ^64^ ^]^ EPR spectroscopy provided additional support for the proposed electron transfer mechanism. While the pristine cages showed no paramagnetic signals, the iodine‐saturated composites displayed distinct paramagnetic signals (Figure S58, Supporting Information). This suggests the formation of radical species in I₂@**MC**, likely arising from electron exchange between the electron‐rich cage framework and the electron‐deficient iodine species.^[^ ^9^ ^]^ FTIR analysis revealed noticeable spectral changes upon I_2_ adsorption, indicating interactions site of cage and iodine species. The intensity of the C = C stretching vibration at 1506‐1467 cm^−1^ associated with the aromatic rings decreased, suggesting the formation of iodine‐π complexes. Additionally, the C = N and C−N stretching vibration peaks shifted from 1605 to 1609 cm^−1^ and from 1325 to 1320 cm^−1^ in **MC1**, from 1635 to 1632 cm^−1^ and from 1334 to 1331 cm^−1^ in **MC2**, from 1633 to 1624 cm^−1^ and from 1327 to 1342 cm^−1^ in **MC3**, and from 1615 to 1608 cm^−1^ and from 1325 to 1310 cm^−1^ in **MC4**, respectively, pointing to the formation of Lewis acid‐base adducts between iodine and the nitrogen atoms in the C = N and C‐N groups (Figure S55, Supporting Information). These spectral shifts and intensity changes provide strong evidence that both the aromatic rings and nitrogen‐containing groups serve as key adsorption sites for iodine within the cage structure.

XPS was employed to investigate the nature of trapped iodine and its interaction with the cage framework. Compared to the pristine cages, the XPS spectra of the iodine‐loaded cages exhibited two distinct peaks in the iodine region, indicating the presence of two different iodine oxidation states (Figure 3a; Figures S67–S69, Supporting Information). The I 3d spectra showed peaks at 632.18 and 620.17 eV for I₂@**MC1**, 631.91 and 620.47 eV for I₂@**MC2**, 631.82 and 620.38 eV for I₂@**MC3**, and 632.06 and 620.58 eV for I₂@**MC4**, which correspond to the I 3d₃_/_₂ orbitals of molecular iodine. Additionally, peaks associated with the I 3d₅_/_₂ orbitals were observed at 630.32 eV and 618.78 eV for I₂@**MC1**, 630.01 eV and 618.54 eV for I₂@**MC2**, 629.80 eV and 618.36 eV for I₂@**MC3**, and 630.04 eV and 618.54 eV for I₂@**MC4** (Figure 3c; Figures S70–S72, Supporting Information).^[^ ^65^, ^66^ ^]^ A quantitative analysis of the XPS peak areas revealed the relative abundance of I₃⁻ and I₅⁻ species within the cages: 35.2% I₃⁻ and 64.8% I₅⁻ for I₂@**MC1**, 69% I₃⁻ and 31% I₅⁻ for I₂@**MC2**, 63% I₃⁻ and 37% I₅⁻ for I₂@**MC3**, and 37.3% I₃⁻ and 62.7% I₅⁻ for I₂@**MC4**. Notably, we observe that cages such as **MC1** and **MC4**, which contain a higher density of electron‐rich nitrogen sites, exhibit a greater proportion of I₅⁻ relative to I₃⁻. In contrast, **MC2** and **MC3**—lacking these nitrogen‐rich functionalities—display a predominance of I₃⁻ over I₅⁻. This trend suggests that stronger electron donation from nitrogen atoms favors extended polyiodide formation, as the delocalized electron density encourages the transformation of I₃⁻ into higher polyiodide species like I₅⁻. The pristine cages (**MC1**, **MC2**, **MC3**, and **MC4**) exhibited N 1s peaks at 400.72, 401.17, 400.71, and 400.35 eV, respectively, which are characteristic of C‐N bonds within the ligand framework. After iodine adsorption, a slight shift toward lower binding energies was observed at 400.67 eV for I₂@**MC1**, 401.12 eV for I₂@**MC2**, 400.67 eV for I₂@**MC3**, and 400.33 eV for I₂@**MC4**. Notably, new peaks emerged at higher binding energies, specifically at 401.88 eV for I₂@**MC1** and 401.08 eV for I₂@**MC4** (Figure 3b; Figure S70–S72, Supporting Information).^[^ ^44^ ^]^ These new peaks are attributed to the formation of charge transfer complexes between the cage and iodine, indicating significant electron density transfer from the sp^2^ nitrogen units within the cage to the adsorbed iodine species. Shifts in the C 1s XPS peaks were also detected before and after iodine adsorption (Figure S73, Supporting Information), suggesting that the adsorbed iodine interacts electronically with the aromatic C = C bonds within the ligand framework. The presence of multiple polyiodide species (I₂, I₃⁻, and I₅⁻) within the cages indicates that both physisorption and chemisorption contribute to the adsorption process.

Based on these observations, a two‐step adsorption mechanism can be proposed. Initially, molecular I₂ undergoes physisorption within the internal cavities of the cage framework under vapor‐phase conditions. This is followed by strong electronic interactions between the iodine and the electron‐rich nitrogen sites of the cage, promoting electron transfer and the subsequent formation of I₃⁻ and I₅⁻ through halogen bonding.^[^ ^15^ ^]^ This complex interplay between physisorption and chemisorption, mediated by the heteroatoms (nitrogen) and aromatic moieties (phenyl and naphthalene groups) within the cage, accounts for the high iodine uptake capacity observed for these materials.

### 2.4 Static Iodine Adsorption in Aqueous Solution

**Figure 4:**
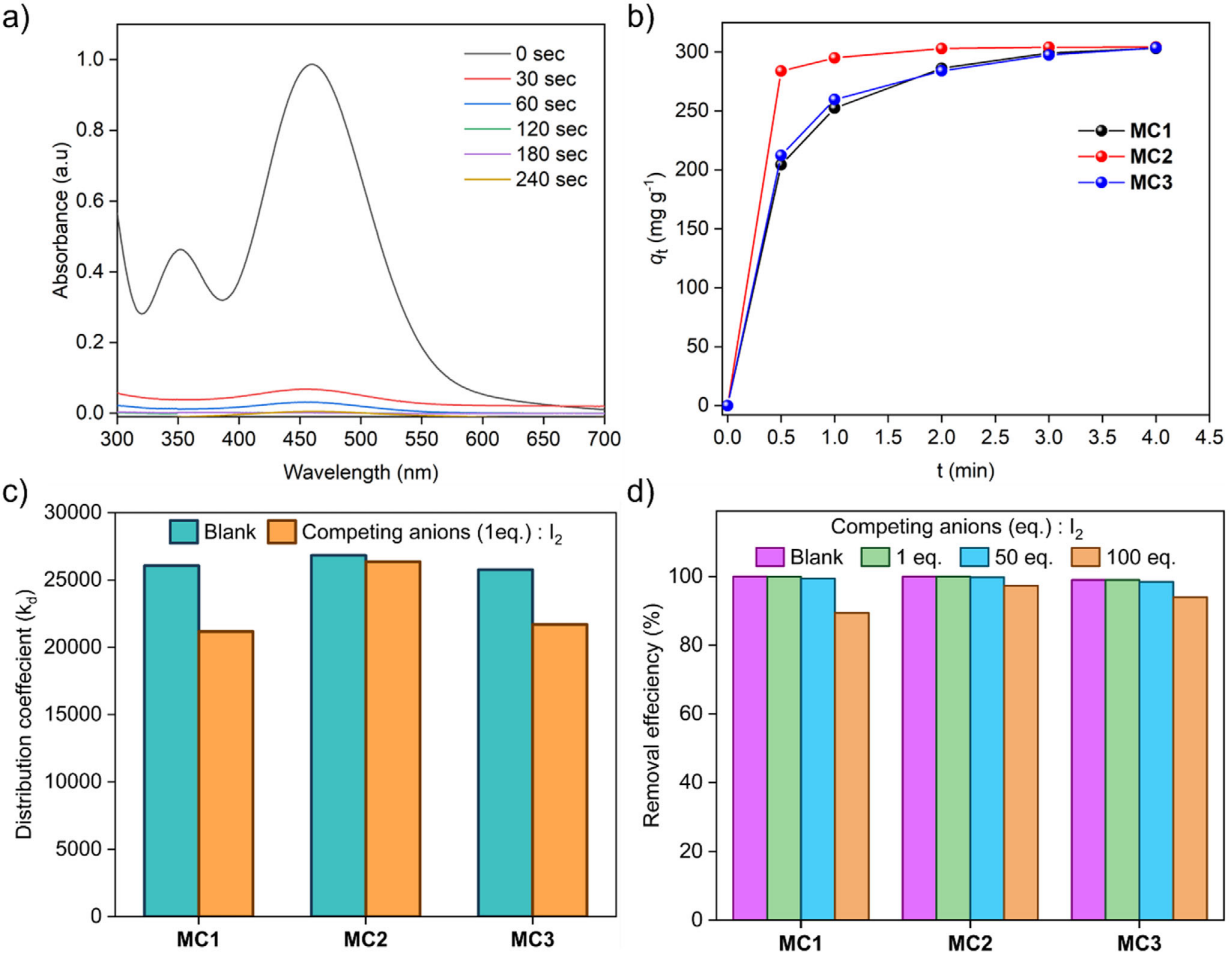
a) Time‐dependent UV–vis spectra showing a rapid decline in iodine concentration in water after introducing **MC2**. b) Adsorption kinetics of iodine from aqueous solution by cage compounds **MC1**, **MC2**, and **MC3**. c) Distribution coefficients (K_d_) of the cages in the presence of a 1‐equivalent competitive anion mixture. d) Iodine adsorption efficiency of the cages in aqueous solutions containing a 100‐fold excess of competing anions (F⁻, Cl⁻, Br⁻, NO₃⁻, and SO₄^2^⁻) (Blank data represents the system without any added competing anions).

Most research on iodine capture has focused on vapor‐phase adsorption, primarily evaluating the iodine uptake capacity under controlled gas‐phase conditions. While significant progress has been made in this area, the adsorption of iodine from aqueous solutions has also received special attention. However, translating these findings into practical applications for the treatment of iodine‐contaminated wastewater remains challenging, especially since such wastewater often contains both molecular iodine (I₂) and polyiodide (I₃⁻) in the presence of iodide (I⁻). To test the adsorption capacity of the cages in aqueous media, static iodine adsorption experiments were performed. For each experiment, 15 mg of water‐insoluble cage (**MC1**, **MC2**, and **MC3**) was divided into five vials, with 3 mg of cage material in each vial. A 3 mL solution of 1.2 mM aqueous iodine was added to each vial. The solution rapidly changed from brown to colorless within a minute, indicating quick iodine uptake (Figure S79, Supporting Information). After centrifugation, the residual iodine concentration was measured using UV–vis spectroscopy (**Figure** **4**a; Figures S75 and S76, Supporting Information). The adsorption peak at 450 nm decreased significantly over time (Figure S77, Supporting Information). Within 60 sec of cage addition, the removal efficiencies of **MC1**, **MC2**, and **MC3** were determined to be 82.76%, 96.83%, and 85.16%, respectively, indicating rapid adsorption kinetics. Among the three, **MC2** demonstrated the fastest adsorption, reaching equilibrium within 120 s, whereas **MC1** and **MC3** required ≈180 s (Figure 4b).

**Figure 5:**
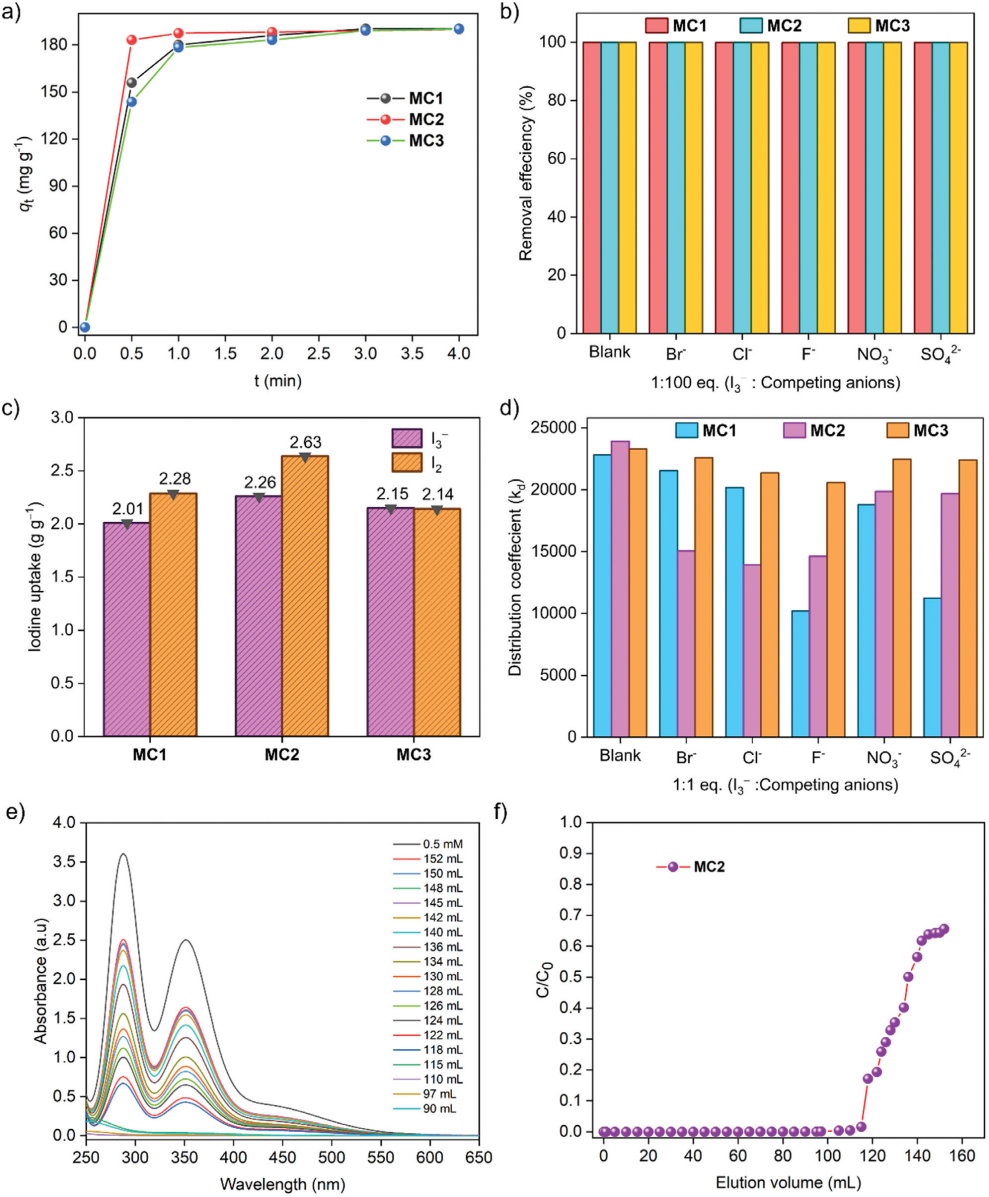
a) Pseudo‐second‐order kinetic modelling of I₃⁻ adsorption by cage compounds **MC1**, **MC2**, and **MC3**. (b) Removal efficiencies of I₃⁻ from aqueous solutions in the presence of a 100‐fold excess of competing anions (F⁻, Cl⁻, Br⁻, NO₃⁻, and SO₄^2^⁻). c) I₂ and I₃⁻ uptake capacities of **MC1**, **MC2**, and **MC3** from aqueous solution. d) Relative distribution coefficients (K_d_) for I₃⁻ uptake by **MC1**, **MC2**, and **MC3** in the presence of 1 equivalent of individual competing anions (Blank refers to conditions in the absence of competing anions). e) UV–vis absorption spectra of the eluent from dynamic flow‐through adsorption experiments using 0.5 mM I₃⁻ solutions and **MC2** as the adsorbent. f) Corresponding breakthrough curves from dynamic flow‐through adsorption experiment.

To determine the maximum iodine uptake capacity, 15 mg of each cage was added to 200 mL of a 1.2 mM iodine solution and stirred for 48 h. UV–vis spectroscopy analysis revealed that the uptake capacities for **MC1**, **MC2**, and **MC3** were 2.14, 2.63, and 2.28 g g^−1^, respectively (Figure 5c; Figure S80, Supporting Information). These values are comparable to the best‐performing materials reported for iodine adsorption from aqueous solutions (Table S15, Supporting Information). The adsorption kinetics were consistent with a pseudo‐second‐order model, with correlation coefficients (R^2^) greater than 0.99, indicating a chemical interaction between the cage and iodine molecules (Figure S78, Supporting Information). The calculated rate constants for **MC1**, **MC2**, and **MC3** were 0.02 198, 0.1463, and 0.02 262 g mg^−1^ min^−1^, respectively (Table S5, Supporting Information). **MC2**’s superior adsorption rate aligns with its high iodine removal efficiency. To investigate the adsorption mechanism, the data were fitted to different isotherm models. The Freundlich model provided a better fit (R^2^ = 0.95–0.99) than the Langmuir model (R^2^ = 0.91–0.96), suggesting that iodine adsorption primarily follows a multilayer chemical adsorption mechanism (Figures S83–S85 and Table S6, Supporting Information).

Selective iodine adsorption was further evaluated through competitive adsorption experiments using simulated wastewater containing fluoride (F^−^), chloride (Cl⁻), bromide (Br⁻), nitrate (NO₃⁻), and sulfate (SO₄^2^⁻). The cages were exposed to 1.2 mM iodine solution in the presence of varying concentrations (1 to 100 equivalents) of competing anions.^[^ ^60^ ^]^ UV–vis spectroscopy showed no significant reduction in iodine uptake in the presence of these anions. Even at 100‐fold excess of competing anions, the iodine removal efficiency remained above 95% for all cages compared to anion‐free controls (Figure 4d). This exceptional selectivity is attributed to the strong interactions within the hydrophobic cavity of the cages, which effectively prevents interference from other anions. The distribution coefficient (K_d_), which reflects the binding affinity between the adsorbent and the target molecule, was calculated to evaluate the strength of iodine binding. The K_d_ value for the cages was ≈10⁴ mL g⁻¹ in the presence of one equivalent of competing anions, confirming the high affinity of the cage structure for iodine molecules (Figure 4c; Figure S82, Supporting Information).^[^ ^67^ ^]^ These findings highlight the strong potential of these cage‐based materials for practical iodine removal from contaminated aqueous environments.

In addition to molecular iodine (I₂), iodide (I⁻) and triiodide (I₃⁻) are key iodine species in aqueous environments. In iodine‐contaminated wastewater, I₂ readily reacts with I⁻ to form I₃⁻ via the following equilibrium: I_2_ + I^−^ ⇌ I_3_^−^. Effective removal of I₃⁻ is therefore crucial for comprehensive iodine remediation. The iodide and triiodide adsorption capabilities of the synthesized cages (**MC1**, **MC2**, and **MC3**) were investigated using time‐resolved UV‐Vis spectroscopy. In the experimental setup, 3 mg of each cage was added to 3 mL of an aqueous solution containing 0.5 mM I₃⁻. The characteristic absorption peaks of I₃⁻ at 290 nm and 355 nm gradually diminished over time, with a corresponding color change from yellow to colorless (Figures S87–S89, Supporting Information). These observations indicated rapid I₃⁻ removal by all cages (**Figure** **5**a). Notably, within one min of exposure to **MC2**, the iodide concentration in the solution dropped to 3 ppm, while concentrations declined to 5.4 and 4.2 ppm after 4 min of exposure to **MC1** and **MC3**, respectively (Figure S91, Supporting Information). Among the tested materials, **MC2** demonstrated the fastest iodine removal kinetics, likely due to its larger pore size, which facilitates enhanced diffusion and adsorption of iodine species within the cage structure.

Kinetic studies showed that the pseudo‐second‐order model accurately described the I₃⁻ adsorption process, indicating a chemisorption mechanism (Figure S92, Supporting Information). The calculated rate constants for **MC1**, **MC2**, and **MC3** were 0.0839, 0.4251, and 0.0597 g mg^−1^ min^−1^, respectively (Table S7, Supporting Information). Notably, **MC2** exhibited the highest rate constant among reported adsorbents (Table S17, Supporting Information), surpassing polymeric, COF, and MOF‐based materials.

Equilibrium adsorption studies revealed that the Freundlich model (R^2^ = 0.91–0.96) provided a better fit than the Langmuir model (R^2^ = 0.81–0.89), suggesting a multilayer adsorption process for I₃⁻ (Figures S96–S98 and Table S8, Supporting Information). Maximum I₃⁻ uptake capacities were determined by immersing 6 mg of each cage in a KI₃ solution (prepared by mixing 3 g KI and 1.5 g I₂ in 6 mL of water) and stirred for 48 h. Titration of the filtrates with sodium thiosulfate revealed maximum uptake capacities of 2.15, 2.26, and 2.01 g g^−1^ for **MC1**, **MC2**, and **MC3**, respectively (Figure 5c). Selective adsorption studies were conducted to evaluate the effect of competing anions (F⁻, Cl⁻, Br⁻, NO₃⁻, and SO₄^2^⁻) at concentrations ranging from 1 to 100 equivalents. Remarkably, all cages maintained ≈97% I₃⁻ removal efficiency even in the presence of high concentrations of competing anions (Figure 5b; Figures S93–S95, Supporting Information). The calculated distribution coefficients (K_d_) were on the order of 10⁴ mL g^−1^, comparable to the values obtained in anion‐free conditions, further confirming the strong binding affinity of the cages for I₃⁻ in complex environments (Figure 5d; Figures S93–S95, Supporting Information). Further kinetic analysis revealed enhanced iodine removal rates when potassium iodide was added to the system. For instance, without KI, the observed iodine adsorption rate constant for **MC2** was 0.1463 g mg^−1^ min^−1^. In contrast, the presence of 20 equivalents of KI increased the rate constant to 0.7418 g mg^−1^ min^−1^, while **MC2**’s iodine removal efficiency improved from 96.83% to 99.8% within 60 sec (Figures S99–S101 and Table S9, Supporting Information). These results suggest a dominant co‐adsorption mechanism involving both I₂ and I⁻ ions. The proposed adsorption mechanism involves halogen bonding interactions between I₃⁻ and specific ligand moieties in the cage framework. This interaction appears to shift the equilibrium between I₂ and I₃⁻, favouring I₃⁻ adsorption and enhancing overall iodine removal efficiency. Despite exhibiting comparable overall adsorption capacities, **MC2** demonstrated significantly faster kinetics than **MC1** and **MC3**. This superior performance is attributed to **MC2**’s naphthalene‐based core ligand, which imparts greater hydrophobicity to the cage structure compared to the pyridine‐based core in **MC1** and the benzene‐based core in **MC3**. Additionally, **MC1** and **MC2** possess a higher cationic charge (+6) than **MC3** (+4), which enhances electrostatic interactions with anionic iodine species, further promoting rapid adsorption. To confirm the structural stability, we recorded ^1^H NMR of the molecular cages before and after iodine exposure. The absence of significant peak shifts indicated that the cage frameworks remained intact throughout the adsorption process (Figures S125–S128, Supporting Information). These results highlight the strong potential of these molecular cages as highly efficient sorbents for iodine removal and water purification in real‐world applications.

### 2.5 Dynamic Adsorption of Iodine in Aqueous Solution

Building on the successful static adsorption of iodine and tri‐iodide by **MC1**, **MC2**, and **MC3** in aqueous media, their performance in dynamic flow‐through systems was evaluated to explore their potential for real‐world iodine remediation.^[^ ^15^, ^43^ ^]^ This step is critical for assessing the industrial applicability of these materials in radioactive iodine removal from contaminated water streams. Dynamic adsorption experiments were conducted using columns packed with 15 mg of dried cage material, secured between dried cotton layers at both ends. Aqueous solutions of I₃⁻ (0.5 mM) were passed through the columns at a flow rate of 1 mL min⁻¹. The immediate decolorization of the iodine solution upon passage through the columns indicated effective adsorption (Figure S104, Supporting Information). UV–vis spectroscopy confirmed high removal efficiencies under dynamic conditions. In the absence of competing anions, I₃⁻ removal efficiencies reached 98.2% for **MC1**, 99.7% for **MC2**, and 98.8% for **MC3** (Figure 5e; Figures S102 and S103, Supporting Information). Even in the presence of 50 equivalents of competing anions, the efficiencies remained high at 95.6%, 96.5%, and 94.9% for **MC1**, **MC2**, and **MC3**, respectively. The breakthrough volumes, determined through UV‐Vis analysis of the eluent, were measured as 3.53 L g^−1^ for **MC1**, 7.8 L g^−1^ for **MC2**, and 3.0 L g^−1^ for **MC3** at a concentration of 0.5 mM (Figure 5f; Figures S102 and S103, Supporting Information). The consistently high iodine removal efficiency (≈98%) before reaching the breakthrough point highlights the substantial iodine uptake capacity of these cages under dynamic conditions. The higher breakthrough volume for **MC2** reflects its faster adsorption kinetics and greater iodine binding capacity compared to **MC1** and **MC3**. These findings demonstrate that the cages possess both high adsorption efficiency and selectivity, making them strong candidates for iodine capture in practical settings.

A key challenge in water treatment is the removal of low‐concentration iodine from contaminated water sources (Section S11, Supporting Information). To evaluate the cages' performance under realistic conditions, dynamic adsorption was tested using I₃⁻ solutions at 5 ppm in both simulated seawater (representing industrially contaminated water) and distilled water (representing groundwater). The solutions were passed through a column packed with 10 mg of the adsorbent. The concentration of residual iodine in the treated water was quantified using the leuco crystal violet method (Scheme S7, Supporting Information).^[^ ^16^, ^68^ ^]^ The cages achieved 99.99% removal of I₃⁻ from both seawater and distilled water. Final iodine concentrations in the treated water were reduced to 28 ppb, 32 ppb, and 29 ppb in seawater and 13 ppb, 11 ppb, and 14 ppb in distilled water for **MC1**, **MC2**, and **MC3**, respectively. These residual levels are comparable to the natural background iodine concentrations in water and are well below the threshold considered hazardous to human health. These results highlight the strong adsorption capacity, rapid kinetics, and exceptional selectivity of the cage materials, positioning them as promising candidates for iodine removal in both industrial and domestic water purification systems.

### 2.6 Density Functional Theory Calculations

To elucidate the adsorption mechanisms of molecular iodine and triiodide on metal–organic cages, Density functional theory (DFT) calculations were performed (Section S11, Supporting Information). These computational studies support the experimental findings and provide molecular‐level insight into the nature of interactions involved in iodine capture. Geometry optimizations of energy‐minimized structures revealed favourable binding sites for both I₂ and I₃⁻ within the cages (Figures S147–S154, Supporting Information).^[^ ^69^ ^]^ Electrostatic potential (ESP) maps highlighted distinct regions of positive potential near the Pd(II) centres and between the aromatic ligand frameworks—key sites likely to promote electrostatic attraction with iodine species. The calculations demonstrated that both I₂ and I₃⁻ exhibit significantly stronger binding energies when encapsulated within the cage compared to external binding, emphasizing the critical role of the confined internal environment in enhancing adsorption affinity.^[^ ^70^, ^71^, ^72^ ^]^ Notably, the binding energy for I₂, ranging between 7–24 kcal mol^−1^ was consistently lower than that for I₃⁻ (Figures S147–S154, Supporting Information), showing the greater affinity of the cages for triiodide. This enhanced binding is primarily attributed to strong electrostatic interactions between the anionic I₃⁻ and the cationic framework. Additionally, supramolecular forces—including C–H⋅⋅⋅I, metal–halogen interactions, π⋅⋅⋅I contacts, and van der Waals interactions—collectively contribute to the stabilization of the iodine species within the cage.^[^ ^8^ ^]^ In summary, DFT results confirm that the cationic metal–organic cages serve as efficient hosts for I₂ and I₃⁻, forming stable complexes through a combination of electrostatic and non‐covalent interactions.

### 2.7 Iodine Removal from Organic Solvent

**Figure 6:**
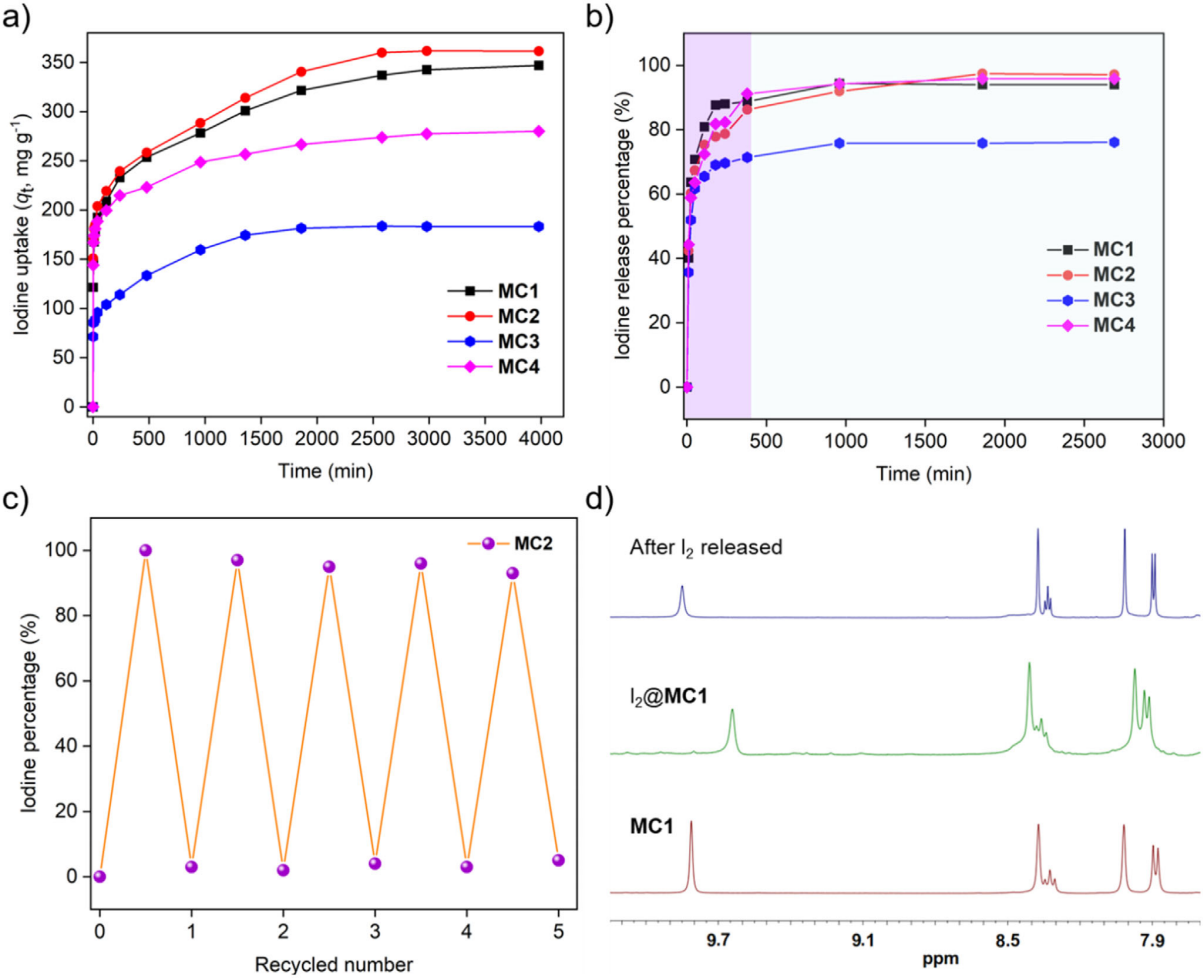
a) Time‐dependent iodine adsorption isotherms of **MC1**, **MC2**, **MC3**, and **MC4** from *n*‐hexane solution b) Thermal desorption of iodine from I_2_‐loaded cages at 110 °C under ambient pressure. c) Iodine adsorption efficiency of the cages after multiple regeneration cycles. d) Partial ¹H NMR spectra (DMSO‐*d*₆) of **MC1**: pristine cage (bottom), after I₂ adsorption from aqueous solution (middle), and after I₂ release (top).

To assess the versatility of metal‐organic cages for iodine adsorption, their performance was tested in the non‐polar solvent *n*‐hexane. A 5 mg sample of each cage (**MC1**, **MC2**, **MC3**, and **MC4**) was introduced into 6 mL of a 1.5 mM iodine solution in *n*‐hexane and stirred. The progressive decrease in the iodine absorption peak at 523 nm in the UV–vis spectra confirmed the adsorption process. The gradual decolorization of the solution further supported effective iodine removal from the organic phase (**Figure** **6**a; Figures S107–S111 and S114, Supporting Information). Equilibrium adsorption in **MC1** and **MC2** was reached within 50 h, while **MC3** and **MC4** achieved equilibrium more rapidly, within 31 h (Figures S107–S111 and S112, Supporting Information). The equilibrium adsorption capacities were calculated as 346.98 mg g^−1^ for **MC1**, 361.66 mg g^−1^ for **MC2**, 280.04 mg g^−1^ for **MC3**, and 183.27 mg g^−1^ for **MC4** (Figure 6a). Kinetic analysis showed that the adsorption process followed a pseudo‐second‐order model (Figure S113 and Table S10, Supporting Information), indicating a chemisorption‐driven mechanism. The maximum adsorption capacities derived from the pseudo‐second‐order model were 363.64 mg g^−1^ for **MC1**, 392.22 mg g^−1^ for **MC2**, 205.65 mg g^−1^ for **MC3**, and 385.43 mg g^−1^ for **MC4**, closely matching the experimentally observed iodine uptake from *n*‐hexane (Figure S113, Supporting Information).

Furthermore, the adsorption equilibrium was well‐described by the Freundlich isotherm model (Figures S115 and S116 and Table S11, Supporting Information), suggesting multilayer adsorption on heterogeneous surface sites. These results demonstrate the strong affinity of the cages for iodine in organic solvents, highlighting their potential for efficient iodine capture in both aqueous and non‐polar media.

### 2.8 Recycling and Reuse Potential of Iodine Adsorption

Thermogravimetric analysis (TGA) demonstrated that the adsorbed iodine within the cage structures desorbed between 40 to 180 °C, suggesting that iodine release can be thermally triggered. To evaluate the recyclability of the cages, they were saturated with iodine by exposing them to iodine vapor at 75 °C for 24 h under ambient pressure. Thermal desorption was then carried out by heating the iodine‐loaded cages at 110 °C. The percentage of iodine released was determined based on the weight loss of the iodine‐loaded cages. Desorption occurred rapidly, with most of the iodine released within 180 min and equilibrium established after ≈30 h. Initial iodine release efficiencies were ≈95% for I₂@**MC1**, I₂@**MC2**, and I₂@**MC4**, while I₂@**MC3** showed a slightly lower efficiency of 68% (Figure 6b). The recyclability of the cages was further tested through five consecutive adsorption‐desorption cycles. After five cycles, the iodine uptake efficiencies remained high at 83%, 88%, 62%, and 82% for **MC1**, **MC2**, **MC3**, and **MC4**, respectively, highlighting the excellent reusability of these materials (Figure 6c; Figures S129–S131, Supporting Information).

Additionally, effective iodine desorption was also achieved using methanol as a solvent. The desorption process was visibly confirmed by the color change of the methanol solution from colorless to dark brown (Figure S123, Supporting Information). UV–vis spectroscopic analysis showed that the absorbance at 290 and 389 nm increased over time, confirming the gradual release of iodine into the solvent (Figures S118–S121, Supporting Information). The desorption kinetics of iodine from the iodine‐loaded cages followed a pseudo‐second‐order kinetic model (Figure S124, Supporting Information), indicating that the rate of iodine release is governed by chemisorption processes.

The pseudo‐second‐order rate constants for desorption were calculated to be 0.4888 g mg^−1^ min^−1^ for **MC1**, 0.8545 g mg^−1^ min^−1^ for **MC2**, 1.3256 g mg^−1^ min^−1^ for **MC3**, and 0.5118 g mg^−1^ min^−1^ for **MC4** (Table S12, Supporting Information).^[^ ^73^ ^]^ This rapid desorption kinetics highlight the exceptional recyclability of the cage materials for iodine capture and release. Post‐desorption structural analysis using ¹H NMR spectroscopy confirmed that the chemical structure of the recovered cages remained unchanged compared to the pristine materials. This result demonstrates the structural integrity and stability of the cages throughout the adsorption‐desorption cycles, reinforcing their potential for long‐term, sustainable iodine capture and release applications.

### 2.9 Adsorption and Mechanistic Investigation of Organic Iodide

Following successful adsorption of iodine from vapor and aqueous phases, the adsorption behaviour of methyl iodide (CH₃I), a key compound in the nuclear industry, was explored using the cage materials.

**Figure 7:**
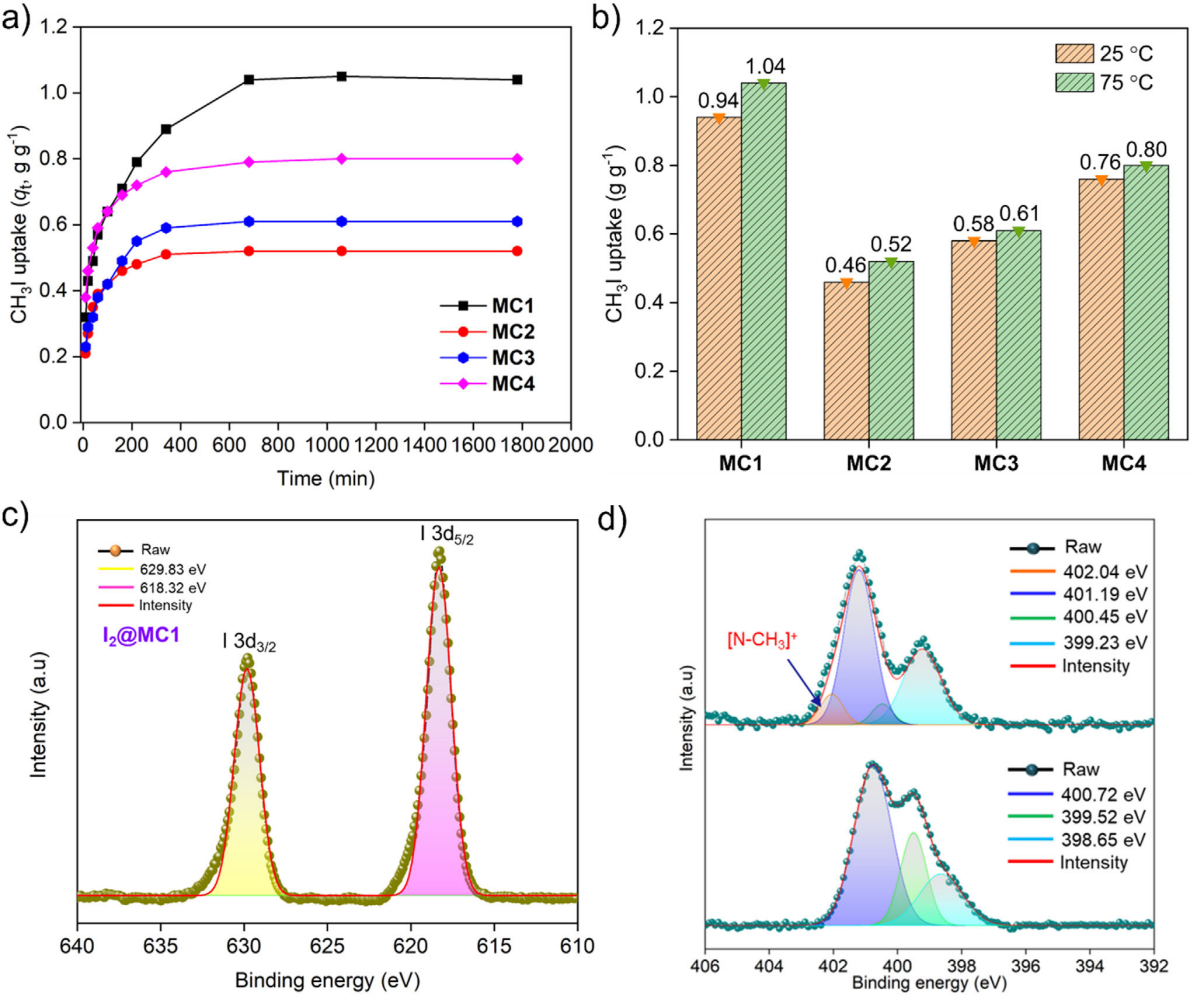
a) Time‐dependent CH₃I uptake by the cages (**MC1**, **MC2**, **MC3**, and **MC4**) at 75 °C under static conditions. b) CH₃I adsorption capacities of all four cages. c) I 3d XPS spectrum of CH₃I@**MC1**. d) N 1s XPS spectra of pristine **MC1** and after CH₃I adsorption.

Given the strong affinity of nitrogen for methyl iodide, static adsorption experiments were conducted at 75 °C under ambient pressure. Gravimetric analysis was used to measure the amount of CH₃I adsorbed by each cage (**Figure** **7**a). The adsorption kinetics were evaluated by monitoring the weight change over time. The saturation adsorption capacities of CH₃I was found to be 1.01, 0.52, 0.61, and 0.80 g g^−1^ for **MC1**, **MC2**, **MC3**, and **MC4**, respectively (Figure 7b). These values are comparable to the capacities of many established porous adsorbents. Equilibrium was reached within 6 h for **MC3** and **MC4**, whereas **MC1** and **MC2** required 12 h to reach saturation. Kinetic analysis showed that CH₃I uptake by the cage materials followed a pseudo‐second‐order kinetic model, indicating a chemisorption process. The rate constants were determined to be 1.4032 g g^−1^ h^−1^ for **MC1**, 2.54 g g^−1^ h^−1^ for **MC2**, 1.2 g g^−1^ h^−1^ for **MC3**, and 1.5117 g g^−1^ h^−1^ for **MC4** (Figure S132 and Table S13, Supporting Information). Adsorption studies at room temperature (25 °C) yielded slightly lower capacities of 0.94, 0.46, 0.58, and 0.76 g g^−1^ for **MC1** to **MC4**, respectively, with **MC1** demonstrating the highest uptake, likely due to the presence of a pyridine core ligand, which enhances binding affinity (Figure 7b). To confirm that CH₃I adsorption occurred within the cage cavities rather than solely on the surface, retention experiments were conducted. CH₃I‐loaded cages were stored under ambient conditions at room temperature for seven days. Minimal weight loss (≈5%) was observed, suggesting strong interactions between CH₃I and the internal binding sites of the cages, ruling out surface condensation as the dominant mechanism (Figure S133, Supporting Information). These results highlight the potential of the cage materials for the effective and long‐term capture of CH₃I, particularly for nuclear waste management and environmental remediation applications.

To elucidate the interaction between CH_3_I and the cage materials, a detailed characterization was performed using FTIR, elemental mapping, EDS, and XPS. EDS and elemental mapping confirmed a uniform distribution of iodine across the surface of the cage materials, providing strong evidence for the successful encapsulation of CH₃I within the cage cavities (Figures S134–S137, Supporting Information). FTIR spectroscopy revealed the appearance of new vibrational bands ≈900−1000 cm^−1^ in CH₃I@**MC1** and CH₃I@**MC4**, respectively, suggesting the formation of C–N bonds upon CH₃I interaction with nitrogen‐containing sites within the cages (Figure S138, Supporting Information).^[^ ^74^, ^75^ ^]^ This supports the formation of ionic species, specifically [–N–CH₃]⁺I⁻, indicating strong covalent interactions between CH₃I and the nitrogen moieties within the cage structures. XPS analysis further confirmed the nature of these interactions. The XPS spectra of CH₃I‐loaded cages showed distinct peaks at 618.32 and 629.83 eV for **MC1**, 618.12 and 629.81 eV for **MC2**, 618.30 and 629.79 eV for **MC3**, and 619.06 and 630.55 eV for **MC4**, corresponding to the 3d₅_/_₂ and 3d₃_/_₂ core levels of iodine (Figure 7c; Figure S140, Supporting Information). These spectral features confirm the encapsulation of CH₃I within the cage structures. Additionally, XPS analysis of the N 1s region revealed a shift in the binding energies of the pyridine nitrogen from 399.65 to 399.23 eV, 399.61 to 399.64 eV, 399.52 to 399.39 eV, and 399.35 to 399.17 eV as well as sp^3^‐N from 400.1 to 401.19 eV, 401.17 to 401.62 eV, 400.71 to 400.98 eV, and 399.58 to 400.69 eV in **MC1**, **MC2**, **MC3**, and **MC4**, respectively, after CH₃I adsorption.^[^ ^44^ ^]^ The emergence of new peaks at higher binding energies (402.04 eV for **MC1** and 401.19 eV for **MC4**) indicates the formation of N–CH₃ bonds, reinforcing the role of nitrogen sites in binding CH₃I (Figure 7d; Figure S139, Supporting Information). Based on these findings, nitrogen‐containing functional moieties such as pyridine and triazole within the cage frameworks were identified as key interaction sites for CH₃I adsorption. In contrast, the absence of such peaks in the CH₃I‐loaded materials of **MC2** and **MC3** suggests that uptake primarily occurs through encapsulation within the cage cavities, rather than via chemical modification. We have also conducted recyclability experiments for CH₃I adsorption by heating at 110 ^ο^C and found that the uptake primarily proceeds through irreversible binding. The strong binding affinity and high adsorption capacities showcasing the potential of these cage materials for the efficient capture and storage of organic iodide, positioning them as promising candidates for nuclear waste remediation and environmental clean‐up.

## 3 Conclusion

In summary, this study presents a systematic approach to designing and synthesizing self‐assembled coordination cages incorporating pyridyl, phenyl, naphthyl, and triazole rings to achieve efficient capture and storage of iodine and methyl iodide. The integration of nitrogen‐rich, electron‐donating aromatic ligands and the strategic modulation of metal‐ligand interactions enabled the formation of robust, nonporous architectures with enhanced adsorption capacity and selectivity. The detailed structural analysis, supported by X‐ray diffraction, NMR spectroscopy, and mass spectrometry, confirms the successful formation of discrete assemblies with high thermal and chemical stability.

These cages exhibited exceptional iodine uptake in the across multiple phases including in aqueous solution under static conditions—demonstrating significantly faster adsorption rates compared to previously reported COF‐ and MOF‐based materials. In dynamic flow‐through experiments, the cages maintained high adsorption efficiencies, with **MC2** achieving a remarkably high elution volume even in a 0.5 mM I₃⁻ solution. The practical utility of these materials was further confirmed through real‐world applications, where water‐insoluble cages effectively removed iodine from both seawater and drinking water, reducing initial concentrations of 5 ppm to ppb levels. Additionally, the cages exhibited outstanding adsorption of methyl iodide vapors under ambient conditions. DFT results confirm that uptake capacity of these cages stems from the combined effect of cationic framework, nitrogen‐rich sites, aromatic π‐electron systems, and confined cavities, which promote efficient host‐guest interactions and charge‐transfer interactions with volatile iodine species. In the case of CH₃I adsorption, the nucleophilic nitrogen sites played a key role in facilitating selective N‐methylation reactions, leading to the formation of stable salts and enhancing the overall adsorption efficiency. Moreover, the recyclability and structural integrity of the cages after multiple adsorption‐desorption cycles highlight their robustness and long‐term applicability.

The insights gained from this work provide a framework for the rational design of metallo‐supramolecular assemblies for targeted guest capture and storage. The findings demonstrate that N‐heteroatom functionalization is a powerful strategy for enhancing host‐guest interactions and optimizing the adsorption efficiency of coordination cages. Future studies may focus on extending this approach to other volatile and hazardous species, further broadening the scope of applications in environmental remediation and nuclear waste management.

[CCDC 2433497 and 2433498, for the cages **MC1** and **MC2**, respectively, contain the supplementary crystallographic data for this paper. These data can be obtained free of charge from The Cambridge Crystallographic Data Centre via www.ccdc.cam.ac.uk/data_request/cif.]

## Conflict of Interest

The authors declare no conflict of interest.

## Supporting information



Supporting Information

Supporting Information

## Data Availability

The data that support the findings of this study are available in the supplementary material of this article.

## References

[1] J. D. Anna , The role of nuclear energy in the global energy transition, Oxford Institute for Energy Studies, UK 2022.

[2] I. E. Agency , Nuclear Power in a Clean Energy System, IEA, Paris 2019.

[3] X. Guo , Nuclear Fission Energy – Carbon Net Zero, Sustainability and Energy Availability, IntechOpen, Rijeka, 2025.

[4] K. Jin , B. Lee , J. Park , Coord. Chem. Rev. 2021, 427, 213473.

[5] F. C. Küpper , M. C. Feiters , B. Olofsson , T. Kaiho , S. Yanagida , M. B. Zimmermann , L. J. Carpenter , G. W. Luther , Z. Lu , M. Jonsson , L. Kloo , Angew. Chem., Int. Ed. 2011, 50, 11598. 10.1002/anie.201100028 22113847

[6] A. Saiz‐Lopez , J. M. C. Plane , A. R. Baker , L. J. Carpenter , R. von Glasow , J. C. Gómez Martín , G. McFiggans , R. W. Saunders , Chem. Rev. 2011, 112, 1773. 22032347 10.1021/cr200029u

[7] W. Xie , D. Cui , S.‐R. Zhang , Y.‐H. Xu , D.‐L. Jiang , Mater. Horiz. 2019, 6, 1571.

[8] A. Sen , S. Sharma , S. Dutta , M. M. Shirolkar , G. K. Dam , S. Let , S. K. Ghosh , ACS Appl. Mater. Interfaces 2021, 13, 34188. 34279084 10.1021/acsami.1c07178

[9] L. Xie , Z. Zheng , Q. Lin , H. Zhou , X. Ji , J. L. Sessler , H. Wang , Angew. Chem., Int. Ed. 2021, 61, 202113724. 10.1002/anie.202113724 34747097

[10] L. Wang , Z. Li , Q. Wu , Z. Huang , L. Yuan , Z. Chai , W. Shi , Environ. Sci. Nano 2020, 7, 724.

[11] T. Pan , K. Yang , X. Dong , Y. Han , J. Mater. Chem. A 2023, 11, 5460.

[12] S. Bolisetty , M. Peydayesh , R. Mezzenga , Chem. Soc. Rev. 2019, 48, 463. 30603760 10.1039/c8cs00493e

[13] L. A. Shaw , J. L. Barreda , SAE Technical Paper Series, NASA 2008.

[14] H. Backer , J. Hollowell , Environ. Health Perspect. 2000, 108, 679. 10964787 10.1289/ehp.00108679 PMC1638306

[15] G. Matthys , A. Laemont , N. De Geyter , R. Morent , R. Lavendomme , P. Van Der Voort , Small 2024, 20, 2404994. 10.1002/smll.202404994 39169707

[16] M. Zhang , J. Samanta , B. A. Atterberry , R. Staples , A. J. Rossini , C. Ke , Angew. Chem., Int. Ed. 2022, 61, 202214189. 10.1002/anie.202214189 36331335

[17] T. A. Nichols , J. S. Morris , V. L. Spate , C. J. Tharp , C. K. Baskett , T. L. Horsman , M. M. Mason , T. P. Cheng , J. Radioanal. Nucl. Chem. 1998, 236, 65.

[18] H. Sepehrpour , W. Fu , Y. Sun , P. J. Stang , J. Am. Chem. Soc. 2019, 141, 14005. 31419112 10.1021/jacs.9b06222 PMC6744948

[19] J. C. Sisson , J. Freitas , I. R. McDougall , L. T. Dauer , J. R. Hurley , J. D. Brierley , C. H. Edinboro , D. Rosenthal , M. J. Thomas , J. A. Wexler , E. Asamoah , A. M. Avram , M. Milas , C. Greenlee , Thyroid 2011, 21, 335. 21417738 10.1089/thy.2010.0403

[20] B. J. Riley , J. D. Vienna , D. M. Strachan , J. S. McCloy , J. L. Jerden , J. Nucl. Mater. 2016, 470, 307.

[21] T.‐S. Chee , Z. Tian , X. Zhang , L. Lei , C. Xiao , J. Nucl. Mater. 2020, 542, 152526.

[22] X. Zhang , J. Maddock , T. M. Nenoff , M. A. Denecke , S. Yang , M. Schröder , Chem. Soc. Rev. 2022, 51, 3243. 35363235 10.1039/d0cs01192d PMC9328120

[23] S. Liu , N. Wang , Y. Zhang , Y. Li , Z. Han , P. Na , J. Hazard. Mater. 2015, 284, 171. 25463231 10.1016/j.jhazmat.2014.10.054

[24] Y. H. Abdelmoaty , T.‐D. Tessema , F. A. Choudhury , O. M. El‐Kadri , H. M. El‐Kaderi , ACS Appl. Mater. Interfaces 2018, 10, 16049. 29671571 10.1021/acsami.8b03772

[25] P. Tian , L. Tang , K. S. Teng , S. P. Lau , Mater. Today Chem. 2018, 10, 221.

[26] B. Azambre , M. Chebbi , ACS Appl. Mater. Interfaces 2017, 9, 25194. 28696664 10.1021/acsami.7b02366

[27] B. Azambre , M. Chebbi , O. Leroy , L. Cantrel , Ind. Eng. Chem. Res. 2018, 57, 1468.

[28] T. Liu , Y. Zhao , M. Song , X. Pang , X. Shi , J. Jia , L. Chi , G. Lu , J. Am. Chem. Soc. 2023, 145, 2544. 36661080 10.1021/jacs.2c12284

[29] Y. Xie , T. Pan , Q. Lei , C. Chen , X. Dong , Y. Yuan , W. A. Maksoud , L. Zhao , L. Cavallo , I. Pinnau , Y. Han , Nat. Commun. 2022, 13, 2878. 35610232 10.1038/s41467-022-30663-3 PMC9130143

[30] S. Song , Y. Shi , N. Liu , F. Liu , ACS Appl. Mater. Interfaces 2021, 13, 10513. 33599122 10.1021/acsami.0c17748

[31] T. He , X. Xu , B. Ni , H. Lin , C. Li , W. Hu , X. Wang , Angew. Chem., Int. Ed. 2018, 57, 10148. 10.1002/anie.201804792 29957830

[32] A.‐N. Au‐Duong , C.‐K. Lee , Cryst. Growth Des. 2017, 18, 356.

[33] H.‐C. Kim , S. Huh , J. Y. Kim , H. R. Moon , D. N. Lee , Y. Kim , CrystEngComm 2017, 19, 99.

[34] X. Qian , B. Wang , Z.‐Q. Zhu , H.‐X. Sun , F. Ren , P. Mu , C. Ma , W.‐D. Liang , A. Li , J. Hazard. Mater. 2017, 338, 224. 28570876 10.1016/j.jhazmat.2017.05.041

[35] H. Wang , N. Qiu , X. Kong , Z. Hu , F. Zhong , Y. Li , H. Tan , ACS Appl. Mater. Interfaces 2023, 15, 14846. 36881562 10.1021/acsami.3c00918

[36] M. Xu , T. Wang , L. Zhou , D. Hua , J. Mater. Chem. A 2020, 8, 1966.

[37] T. Hasell , M. Schmidtmann , A. I. Cooper , J. Am. Chem. Soc. 2011, 133, 14920. 21863835 10.1021/ja205969q

[38] X. Liu , Z. Zhang , F. Shui , S. Zhang , L. Li , J. Wang , M. Yi , Z. You , S. Yang , R. Yang , S. Wang , Y. Liu , Q. Zhao , B. Li , X. H. Bu , S. Ma , Angew. Chem., Int. Ed. 2024, 63, 202411342. 10.1002/anie.202411342 39078740

[39] Q. Mao , S. Yang , J. Zhang , Y. Liu , M. Liu , Adv. Sci. 2024, 11, 2408494. 10.1002/advs.202408494 PMC11615766 39401421

[40] M. Dalapati , A. Das , P. Maity , R. Singha , S. Ghosh , D. Samanta , Inorg. Chem. 2024, 63, 15973. 39140114 10.1021/acs.inorgchem.4c02343

[41] W.‐Y. Pei , J. Yang , H. Wu , W. Zhou , Y.‐W. Yang , J.‐F. Ma , Chem. Commun. 2020, 56, 2491. 10.1039/d0cc00157k PMC11278981 32003396

[42] L. Zhang , Y. Jin , G. H. Tao , Y. Gong , Y. Hu , L. He , W. Zhang , Angew. Chem., Int. Ed. 2020, 59, 20846. 10.1002/anie.202007454 32770618

[43] W. Zhou , A. Li , M. Zhou , Y. Xu , Y. Zhang , Q. He , Nat. Commun. 2023, 14, 5388. 37666841 10.1038/s41467-023-41056-5 PMC10477329

[44] L. He , L. Chen , X. Dong , S. Zhang , M. Zhang , X. Dai , X. Liu , P. Lin , K. Li , C. Chen , T. Pan , F. Ma , J. Chen , M. Yuan , Y. Zhang , L. Chen , R. Zhou , Y. Han , Z. Chai , S. Wang , Chem 2021, 7, 699.

[45] Q. Zhang , T.‐J. Yue , S.‐J. Jiang , H.‐M. Guo , ACS Appl. Polym. Mater. 2024, 6, 5507.

[46] Q. Zhang , N. Li , J. Li , Z. G. Hu , T.‐J. Yue , H.‐M. Guo , Polym. Chem. 2023, 14, 4109.

[47] S. Sharma , M. Sarkar , D. K. Chand , Chem. Commun. 2023, 59, 535. 10.1039/d2cc04828k 36546562

[48] J. E. M. Lewis , E. L. Gavey , S. A. Cameron , J. D. Crowley , Chem. Sci. 2012, 3, 778.

[49] P. Mal , B. Breiner , K. Rissanen , J. R. Nitschke , Science 2009, 324, 1697. 19556504 10.1126/science.1175313

[50] M. Ray , S. Krishnaswamy , A. K. Pradhan , D. K. Chand , Chem. Mater. 2023, 35, 6702.

[51] A. B. Sainaba , M. Venkateswarulu , P. Bhandari , K. S. A. Arachchige , J. K. Clegg , P. S. Mukherjee , J. Am. Chem. Soc. 2022, 144, 7504. 35436087 10.1021/jacs.2c02540

[52] R. Singha , P. De , D. Samanta , Inorg. Chem. 2025, 64, 4367. 40009737 10.1021/acs.inorgchem.4c04965

[53] M. Yoshizawa , J. K. Klosterman , M. Fujita , Angew. Chem., Int. Ed. 2009, 48, 3418. 10.1002/anie.200805340 19391140

[54] D. Samanta , S. Mukherjee , Y. P. Patil , P. S. Mukherjee , Chem. ‐Eur. J. 2012, 18, 12322. 22899180 10.1002/chem.201201679

[55] J. Gemen , M. J. Białek , M. Kazes , L. J. W. Shimon , M. Feller , S. N. Semenov , Y. Diskin‐Posner , D. Oron , R. Klajn , Chem 2022, 8, 2362. 36133801 10.1016/j.chempr.2022.05.008 PMC9473544

[56] M. Yamashina , M. M. Sartin , Y. Sei , M. Akita , S. Takeuchi , T. Tahara , M. Yoshizawa , J. Am. Chem. Soc. 2015, 137, 9266. 26166243 10.1021/jacs.5b06195

[57] R. Singha , P. Maity , D. Samanta , Chem. – Eur. J. 2024, 30, 202401013. 10.1002/chem.202401013 38700019

[58] L. Yang , G. Chang , L. Luo , F. Ding , J. You , Inorg. Chim. Acta 2013, 406, 307.

[59] P. Howlader , S. Mukherjee , R. Saha , P. S. Mukherjee , Dalton Trans. 2015, 44, 20493. 26544720 10.1039/c5dt03185k

[60] Z. Zheng , Q. Lin , L. Xie , X. Chen , H. Zhou , K. Lin , D. Zhang , X. Chi , J. L. Sessler , H. Wang , J. Mater. Chem. A 2023, 11, 13399.

[61] S. Maji , R. Natarajan , Small 2023, 19, 2302902. 10.1002/smll.202302902 37394720

[62] S. Bera , K. Garg , S. K. Samanta , ACS Appl. Nano Mater 2024, 7, 1797.

[63] J.‐Y. Liu , T.‐P. Sheng , C. Li , Z. Wang , F.‐R. Dai , Z.‐N. Chen , Cryst. Growth Des. 2022, 22, 3182.

[64] Y. Ji , J. Huang , C. Liu , C. Ni , H. Yan , Y. David , Y. Qin , ACS Appl. Polym. Mater. 2024, 6, 7478.

[65] K. Cheng , H. Li , J. R. Wang , P. Z. Li , Y. Zhao , Small 2023, 19, 2301998. 10.1002/smll.202301998 37162443

[66] C. Liu , Y. Jin , Z. Yu , L. Gong , H. Wang , B. Yu , W. Zhang , J. Jiang , J. Am. Chem. Soc. 2022, 144, 12390. 35765245 10.1021/jacs.2c03959

[67] S. Fajal , W. Mandal , A. Torris , D. Majumder , S. Let , A. Sen , F. Kanheerampockil , M. M. Shirolkar , S. K. Ghosh , Nat. Commun. 2024, 15, 1278. 38341406 10.1038/s41467-024-45581-9 PMC10858966

[68] J. L. Lambert , G. L. Hatch , B. Mosier , Anal. Chem. 2002, 47, 915.

[69] Y. Xie , T. Pan , Q. Lei , C. Chen , X. Dong , Y. Yuan , J. Shen , Y. Cai , C. Zhou , I. Pinnau , Y. Han , Angew. Chem., Int. Ed. 2021, 60, 22432. 10.1002/anie.202108522 34431190

[70] Y. Ma , J. Pan , H. Rong , L. Liu , Y. Zhang , X. Cao , J. Zhang , T. Liu , N. Wang , Y. Yuan , Adv. Sci. 2025, 00697. 10.1002/advs.202500697 PMC12376611 40391680

[71] P. Chen , X. He , M. Pang , X. Dong , S. Zhao , W. Zhang , ACS Appl. Mater. Interfaces 2020, 12, 20429. 32255599 10.1021/acsami.0c02129

[72] F. Y. Chen , X. X. Lu , Y. H. Luo , J. Li , D. E. Zhang , Y. Yan , Aggregate 2025, 70051.

[73] C. Xiao , J. Tian , F. Jiang , D. Yuan , Q. Chen , M. Hong , Small 2024, 20, 2311181. 10.1002/smll.202311181 38361209

[74] S. Fajal , D. Majumder , W. Mandal , S. Let , G. K. Dam , M. M. Shirolkar , S. K. Ghosh , J. Mater. Chem. A 2023, 11, 26580.

[75] K. Jie , Y. Zhou , Q. Sun , B. Li , R. Zhao , D.‐E. Jiang , W. Guo , H. Chen , Z. Yang , F. Huang , S. Dai , Nat. Commun. 2020, 11, 1086. 32107383 10.1038/s41467-020-14892-y PMC7046611

