# Supplementary material for: Self‐Assembled Pd(II) Nano‐Adsorbents for Iodine and Methyl Iodide Capture in Vapor and Aqueous Phases
Source: Small. 2025 Jun 29;21(33):2504242. doi: 10.1002/smll.202504242 (PMC12372449; doi:10.1002/smll.202504242)
Supplement: Supplementary file 1 — Supporting Information [file SMLL-21-2504242-s002.docx]

**Self-Assembled Pd(II) Nano-Adsorbents for Iodine and Methyl Iodide Capture in Vapor and Aqueous Phases**

*Monotosh Dalapati, ^a^ Raghunath Singha,^a^ Pankaj Maity,^a^* *Debashree Manna,^b^ and Dipak Samanta*^a,c^*

^a^School of Chemical Sciences, National Institute of Science Education and Research (NISER) Bhubaneswar, an OCC of Homi Bhabha National Institute, PO Bhimpur- Padanpur, Via Jatni, District Khurda, Odisha 752050, India.

^b^Institute of Organic Chemistry and Biochemistry, Czech Academy of Sciences, v.v.i., Flemingovo nám. 2, Prague 6, Praha 16610, Czech Republic

^c^Center for Interdisciplinary Sciences (CIS), National Institute of Science Education and Research (NISER), An OCC of Homi Bhabha National Institute (HBNI), PO Bhimpur- Padanpur, Via Jatni, District Khurda, Odisha 752050, India.

E-mail: [dsamanta@niser.ac.in](mailto:dsamanta@niser.ac.in)

**Table of Content**

1. **General Method** …………….…………...…………….…………………………….**S3**
2. **Synthesis and Characterization**………………………………...…………………..**S3**
3. **X-ray Data Collection and Structure Refinement**………………………….….....**S23**
4. **Gas Sorption Isotherm** ……...……………………………………………….....….**S32**
5. **FE-SEM Image** ………………………………...……………………………….….**S33**
6. **EDAX Analysis** ………………………...…………………………………...….…..**S34**
7. **Iodine Vapour Adsorption Experiment** ……………………………………...…..**S37**
8. **Absorption Mechanism Investigation** ………………………………………….....**S41**
9. **Iodine Uptake Investigation in Aqueous Phase** ………...………………………..**S55**
10. **Dynamic I_3_^−^ Uptake in Aqueous Solution** …………………………..….………...**S73**
11. **Low Concentration Iodine Uptake from Aqueous Solution** ………….….……...**S75**
12. **Iodine Adsorption from *n*-Hexane Solution** ………………………..….………....**S76**
13. **Iodine Release and Recyclability Study** ………………………………………..…**S83**
14. **Static Vapor-Phase Capture of Methyl Iodide** ……………….……………….…**S92**
15. **DFT Calculations** ……………………………………………………………........**S101**
16. **Comparative Study of Adsorbents for Vapor Phase Iodine Adsorption** …......**S109**
17. **Reference for Iodine Uptake in Aqueous Media** ……………………..…...…....**S110**
18. **Comparative Analysis of Adsorbent Recyclability for Vapor-Phase Iodine** …**S111**
19. **Comparison of Rate Constant for I_3_^−^ Adsorption from Water** ………………..**S112**
20. **References** ……………………………………………………………..…….........**S112**

**1**. **General Methods**

**Materials and General Procedure**: Commercially available reagents and solvents were used as received without further purifications. The compounds were purified by column chromatography on silica gel (mesh 230–400). NMR spectra were recorded on Bruker Avance III 400 MHz or 700 MHz instruments at 298 K. Chemical shifts (*δ*) are given in ppm relative to residual protio solvent resonances (7.26 ppm for CDCl_3_, 3.31 ppm for CD_3_OD and 2.50 for DMSO-d_6_). The diffusion coefficient of the solvent was used as a calibration standard. Unless otherwise specified, all measurements were conducted at 298 K. High-resolution mass spectra (HR-MS) were recorded on an ESI-TOF (time of flight) mass spectrometer. FT-IR spectra were obtained using a Perkin Elmer spectrophotometer with ATR Gold. UV−Vis experiments were conducted on a Perkin Elmer Lamda 365+ UV−Vis spectrophotometer with a 1 nm slit width. XPS was performed using a PHI5000 Versaprobe III XPS. The Raman spectra were acquired on a Lab RAM HR Evolution, Horiba Scientific, Raman Spectrometer, equipped with a 532 nm laser ULF. Thermal stability was assessed using the Discovery TGA-1 TA analyser in N_2_ atmosphere at a heating rate of 10 ^ο^C min^–1^. EPR spectra were obtained with a Bruker System EMX-microX at 298K and 9.4335 GHz. Typical EPR spectrometer parameters are shown as follows, scan range: 100 G; centre field set: 3480.00 G; time constant: 0.16 ms; scan time: 128.22 s; modulation amplitude: 20.0 G; modulation frequency: 100 kHz; receiver gain: 2.00 × 102; microwave power: 7.14e^−001^ mW; g = 2.007092. Powder X-ray diffraction (PXRD) patterns were collected at room temperature on a Bruker D8 Advance X-ray powder diffractometer with Cu Kα radiation (λ = 1.5418 Å) as the X-ray source. Scanning electron microscopy (SEM) measurements were conducted with a Merlin Compact field effect SEM (FESEM) with a GEMINI-I electron column, Zeiss Pvt. Ltd., Germany. N_2_ sorption analyses were performed at 77 K on a Quantachrome Instruments Autosorb iQ MP automatic volumetric instrument.

**2**. **Synthesis and Characterization**

2,6-di(1*H*-imidazol-1-yl) pyridine (**L1**).^[1]^

**Scheme S1**. Synthesis of the **L1**.

A mixture of CuI (81.39 mg, 0.428 mmol), Cs_2_CO_3_ (2228.12 mg, 6.83 mmol), 1H-imidazole (349.15 mg, 5.13 mmol), and 2,6-dibromopyridine (405 mg, 1.70 mmol) was dissolved in dry DMF (9 mL) and stirred at room temperature for 30 min in a sealed tube. The reaction mixture was then heated to 130 ^ο^C and stirred for 24 h. After completion, the reaction was cooled to room temperature, and DCM (40 mL) was added. The organic layer was extracted using a standard solvent extraction procedure with water. The collected DCM layer was concentrated under reduced pressure. The crude product was then purified by column chromatography on silica gel using 5% MeOH in CHCl_3_ as the eluent, yielding the desired product. Yield = 358 mg, ⁓68%.

^1^H NMR (700 MHz, CD_3_OD): *δ* (ppm) *=* 8.63 (s, 2H), 8.09 (t, *J* = 8.0 Hz, 1H), 7.95 (s, 2H), 7.62 (d, *J* = 8.0 Hz, 2H), 7.16 (s, 2H). ^13^C NMR (176 MHz, CD_3_OD): *δ* (ppm) = 148.00, 142.73, 135.28, 129.28, 116.56, 110.21. HRMS (ESI): C_11_H_9_N_5_, [M + H] ^+^ = 212.09 (calculated) found: 212.0936.


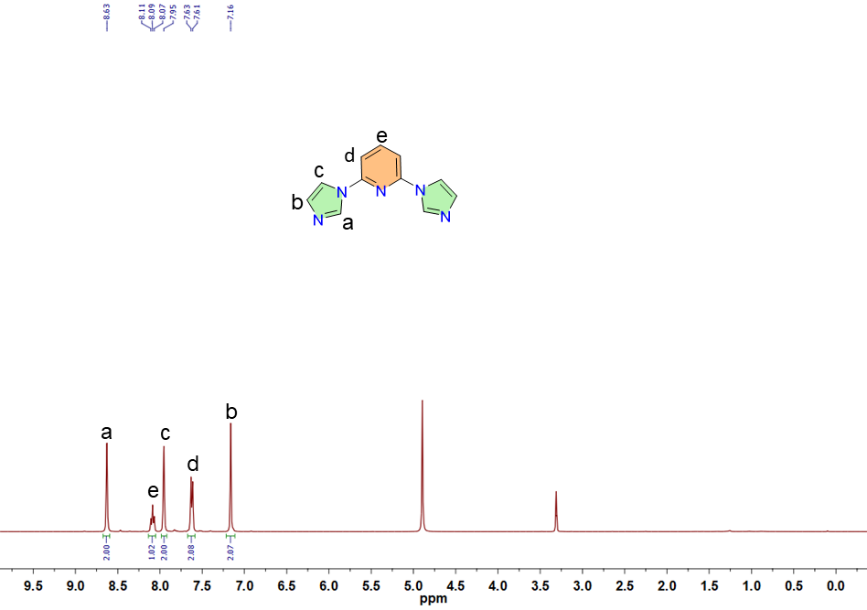


**Figure S1**. ^1^H NMR spectrum of **L1** in CD_3_OD (700 MHz, 298 K).


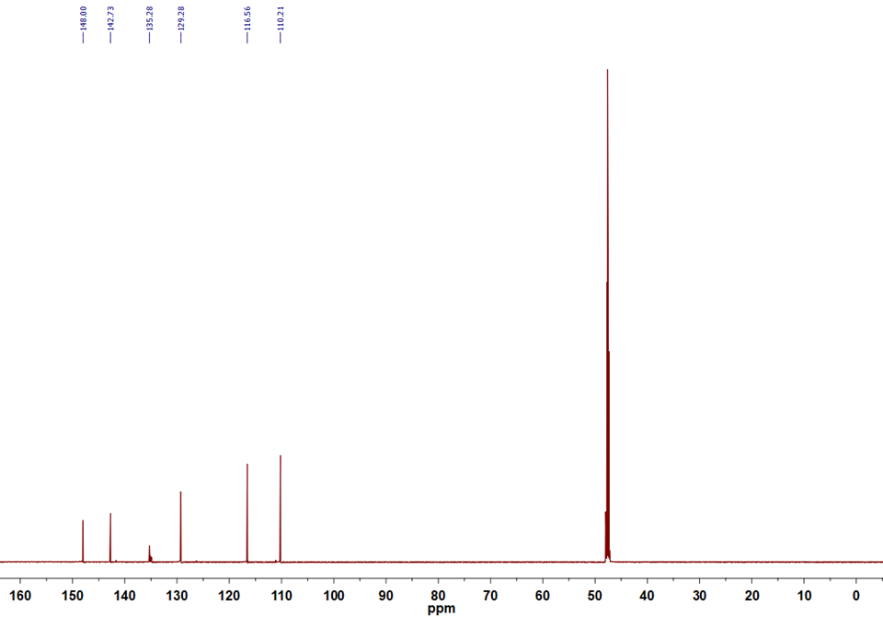


**Figure S2**. ^13^C NMR spectrum of **L1** in CD_3_OD (176 MHz, 298 K).

2,7-di(1*H*-imidazol-1-yl) naphthalene (**L2**).

**Scheme S2**. Synthesis of the ditopic ligand **L2**.

The ligand **L2** was synthesized using a modified version of a previously published procedure.^[2]^ A mixture of CuSO_4_.5H_2_O (56.51 mg, 0.226 mmol), K_2_CO_3_ (2083 mg, 15.07 mmol), 2,7-dibromonaphthalene (412 mg, 1.44 mmol), and 1H-imidazole (918 mg, 13.48 mmol) was thoroughly mixed using a mortar and pestle. The mixture was then transferred to a Schlenk flask under a nitrogen atmosphere. The reaction was heated to 180 °C and stirred for 48 h. After completion, the mixture was cooled to ambient temperature and washed three times with water. The resulting solid residue was extracted with methanol (30 mL), and the solvent was concentrated under vacuum to obtain the pure product as a light-yellow solid. Yield: 263 mg (∼70%).

^1^H NMR (700 MHz, CD_3_OD): *δ* (ppm) *=* 8.25 (s, 1H), 8.01 (d, *J* = 9.1 Hz, 2H), 7.68 (d, *J* = 6.5 Hz, 2H), 7.19 (s, 1H). ^13^C NMR (176 MHz, CD_3_OD): *δ* (ppm) = 135.56, 133.98, 130.87, 129.84, 129.12, 119.86, 118.24, 118.06, 118.03. HRMS (ESI): C_16_H_12_N_4_, [M + H] ^+^ = 261.11 (calculated) found: 239.1043.


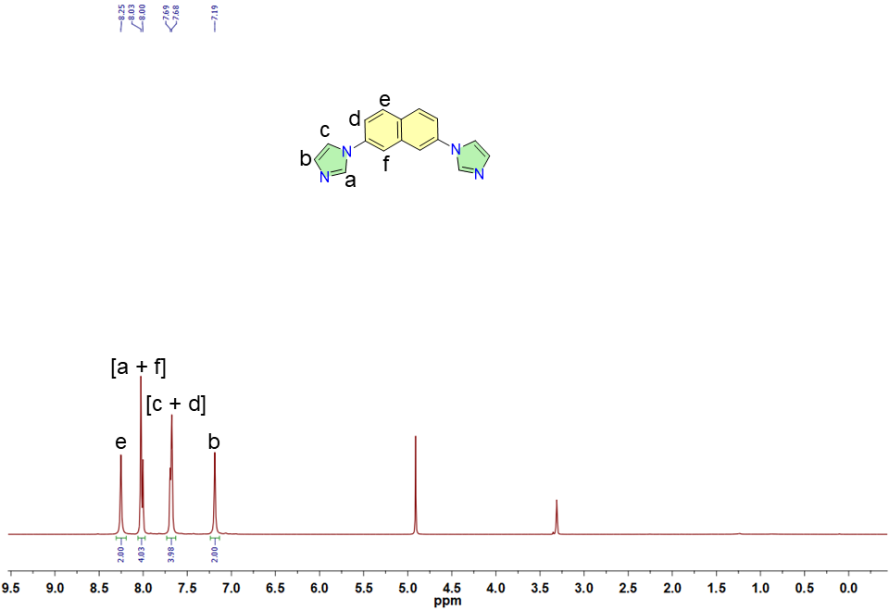


**Figure S3**. ^1^H NMR spectrum of **L2** in CD_3_OD (700 MHz, 298 K).


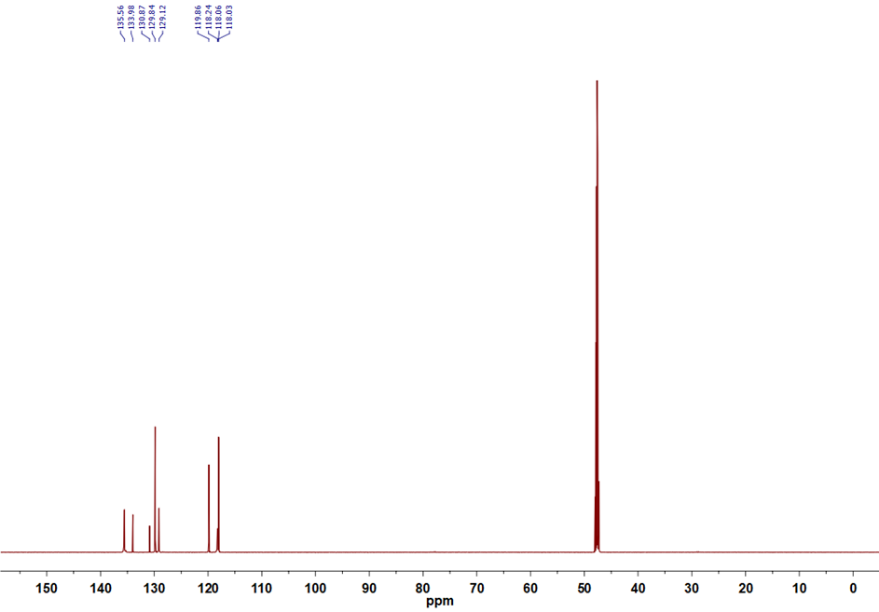


**Figure S4**. ^13^C NMR spectrum of **L2** in CD_3_OD (176 MHz, 298 K).

1,3-bis((1H-imidazol-1-yl) methyl) benzene (**L3**)^[3]^.

**Scheme S3**. Synthesis of the ligand **L3**.

Imidazole (216 mg, 3.18 mmol) and potassium hydroxide (340 mg, 6.06 mmol, crushed using a mortar and pestle) were dissolved in acetonitrile (30 mL) and stirred at room temperature for 2 h. α,α′-m-Dibromo xylene (400 mg, 1.89 mmol) was then added to the reaction mixture, and stir for an additional 2 h. Upon completion, the reaction mixture was filtered using a hirsch funnel to remove insoluble salts. The filtrate was concentrated under reduced pressure. The resulting residue was dissolved in chloroform (40 mL) and washed three times with water until the aqueous phase reached neutrality, as confirmed by pH paper. The organic phase was dried over anhydrous sodium sulfate and concentrated under reduced pressure, yielding a white solid. Yield = 188 mg, ⁓41%.

^1^H NMR (700 MHz, DMSO-*d*_6_): *δ* (ppm) *=* 7.74 (s, 2H), 7.33 (t, *J* = 7.5 Hz, 1H), 7.20 (s, 1H), 7.16 (s, 3H), 7.14 (s, 1H), 6.90 (s, 2H), 5.17 (s, 4H). ^13^C NMR (176 MHz, DMSO-*d*_6_): *δ* (ppm) = 138.74, 137.86, 129.59, 129.16, 127.20, 120.01, 49.74. HRMS (ESI): C_14_H_14_N_4_, [M + H] ^+^ = 239.1200 (calculated) found: 239.1191.


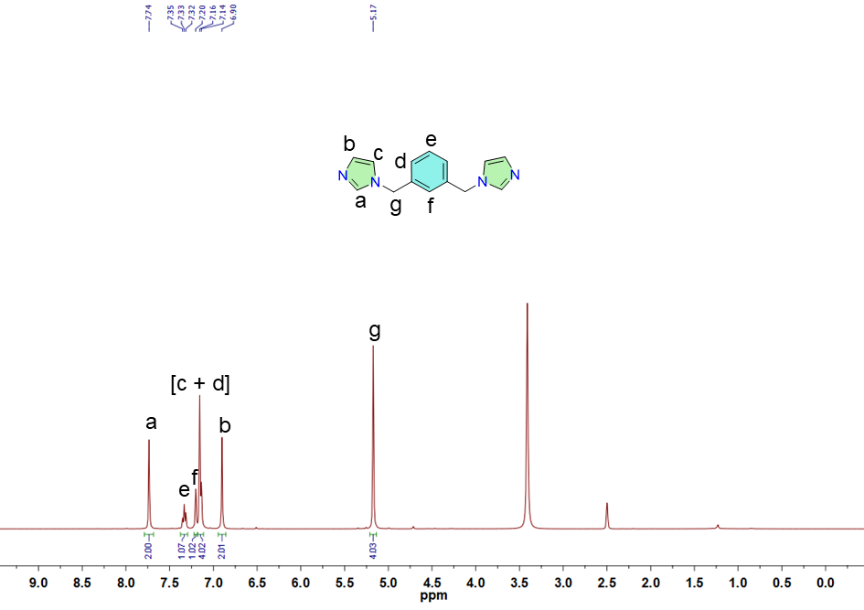


**Figure S5**. ^1^H NMR spectrum of **L3** in DMSO-*d*_6_ (700 MHz, 298 K).


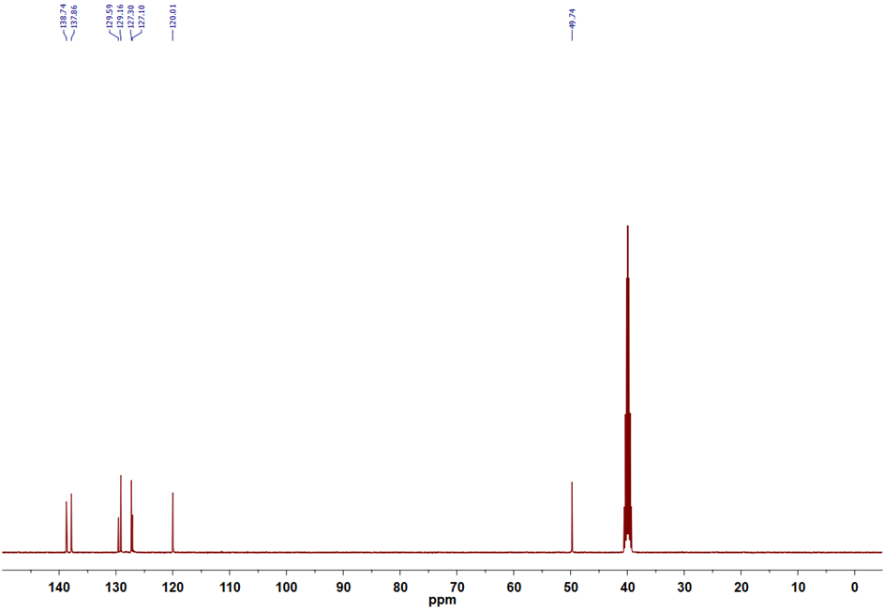


**Figure S6**. ^13^C NMR spectrum of **L3** in DMSO-*d*_6_ (176 MHz, 298 K).

3,3'-(1*H*-pyrazole-3,5-diyl) dipyridine (**L4**).

**Scheme S4**. Synthesis of the ligand **L4**.

Ligand **L4** was synthesized following a previously reported procedure.^[4]^ 3-Cyanopyridine (2.080 g, 20 mmol) and hydroxylamine hydrochloride (1.288 g, 20 mmol) were dissolved in 20 mL of water and transferred to a 50 mL flame-dried, sealed tube. The reaction mixture was then heated at 120 °C for 72 h. Upon completion of reaction, the resulting light-yellow precipitate was collected by filtration, washed thoroughly with cold water, and dried under vacuum to yield the pure product. Yield = 920 mg, ⁓41%.

^1^H NMR (700 MHz, DMSO-*d*_6_): *δ* (ppm) *=* 9.34 (d, *J* = 1.7 Hz, 1H), 9.25 (d, *J* = 1.6 Hz, 1H), 8.90 (dd, *J* = 4.8, 1.3 Hz, 1H), 8.81 (dd, *J* = 4.8, 1.4 Hz, 1H), 8.55 (d, *J* = 8.0 Hz, 1H), 8.44 (d, *J* = 8.0 Hz, 1H), 7.71 (dd, *J* = 7.9, 4.9 Hz, 1H), 7.65 (dd, *J* = 7.8, 4.9 Hz, 1H). ^13^C NMR (176 MHz, DMSO-*d*_6_): *δ* (ppm) = 174.63, 167.05, 154.28, 152.99, 149.00, 148.26, 136.13, 135.29, 125.00, 124.90, 122.70, 120.30. HRMS (ESI): C_12_H_9_N_5_, [M + H] ^+^ = 223.2400 (calculated) found: 223.0913.

**
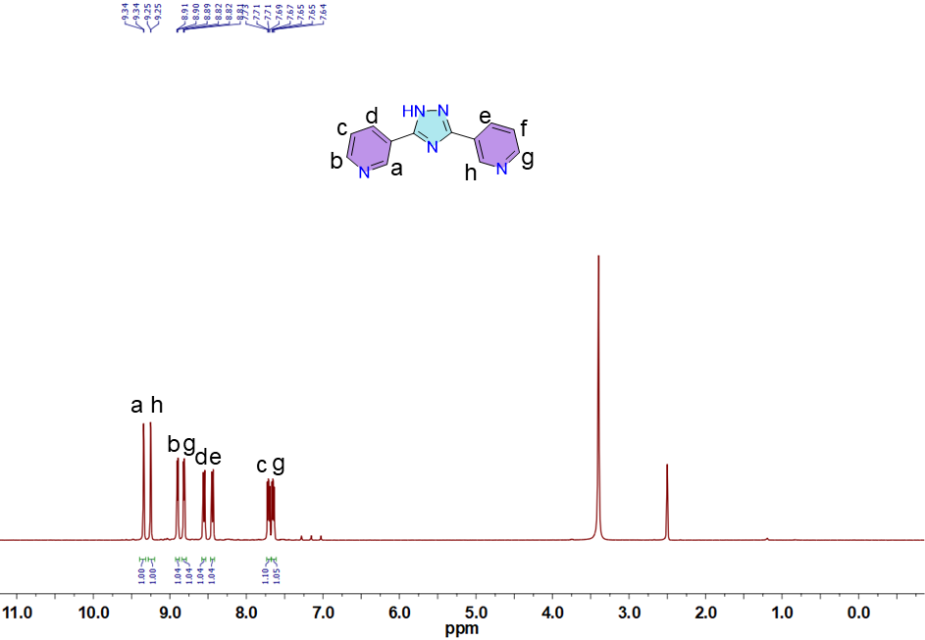
**

**Figure S7**. ^1^H NMR spectrum of **L4** in DMSO-*d*_6_ (700 MHz, 298 K).


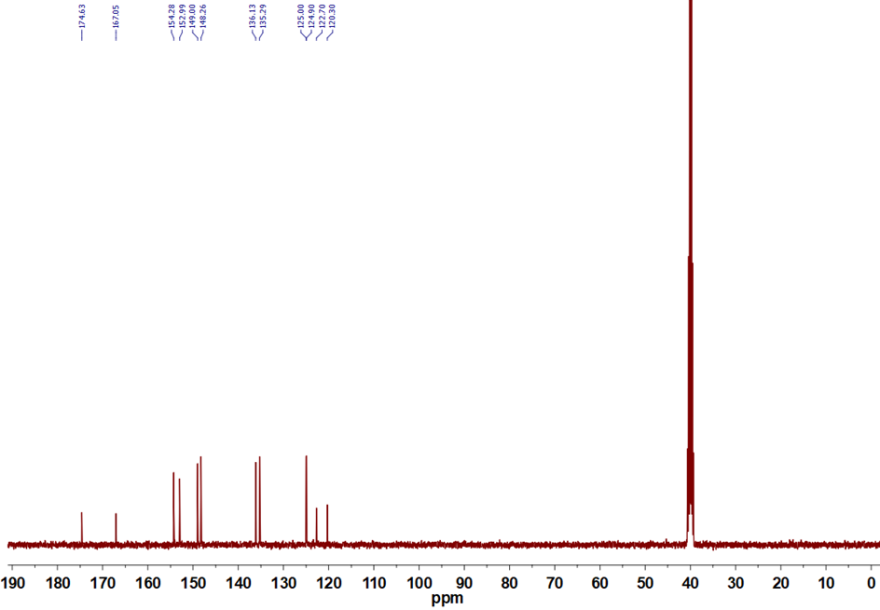


**Figure S8**. ^13^C NMR spectrum of **L4** in DMSO-*d*_6_ (176 MHz, 298 K).

**1,3-di(1H-imidazol-1-yl) benzene (L5)**.

**Scheme S5**. Synthesis of the ligand **L5**.

A mixture of 1,3-diiodobenzene (1005 mg, 3.04 mmol), imidazole (622.5 mg, 9.14 mmol), 1,10-phenanthroline (131.8 mg, 0.73 mmol), potassium carbonate (2105.2 mg, 15.23 mmol), and CuI (104.5 mg, 0.55 mmol) was prepared in a 100 mL double-neck round-bottom flask. Anhydrous DMF (50 mL) was added, and the reaction mixture was refluxed under a nitrogen atmosphere for 36 hours. After cooling to room temperature, the mixture was extracted with DCM for three times. The combined organic layers were dried over anhydrous sodium sulfate, filtered, and evaporated to dryness under reduced pressure. The crude product was purified by flash chromatography using a DCM: MeOH (1:5) eluent to yield the product as a light brown powder. Yield = 361 mg, ⁓56%.

^1^H NMR (400 MHz, CD_3_OD): *δ* (ppm) *=* 8.28 (s, 2H), 7.84 (s, H), 7.69 (s, 2H), 7.64 (dd, *J* = 6.4, 2.7 Hz, 2H), 7.60 (s, 2H), 7.17 (s, 2H). ^13^C NMR (176 MHz, CD_3_OD): *δ* (ppm) = 138.42, 135.79, 131.35, 129.16, 119.35, 118.34, 113.37. HRMS (ESI): C_12_H_11_N_4_, [M + H] ^+^ = 211.0978 (calculated) found: 211.0984.


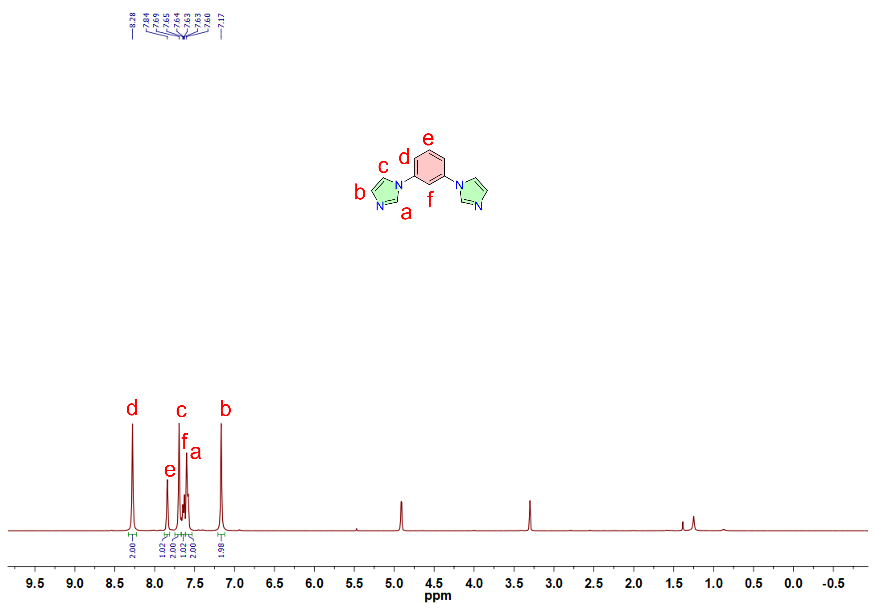


**Figure S9**. ^1^H NMR spectrum of **L5** in CD_3_OD (400 MHz, 298 K).


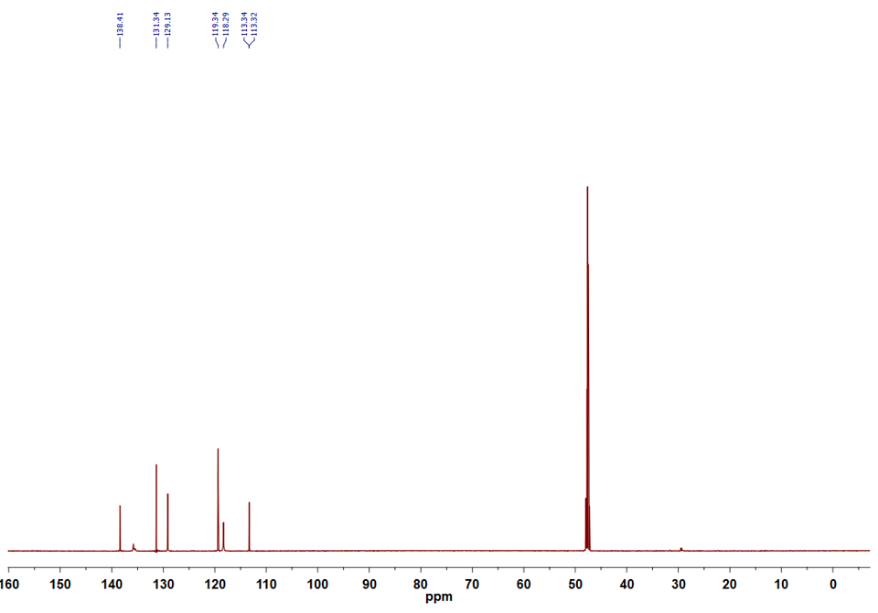


**Figure S10**. ^13^C NMR spectrum of **L5** in CD_3_OD (176 MHz, 298 K).

**General Procedures for the Synthesis of Cages MC1, MC2, MC3 MC4 and MC5**:

A 2 mL clear DMSO solution of Pd(NO_3_)_2_ was added separately to each ligand (**L1**, **L2**, **L3**, **L4** or **L5**), and the mixtures were stirred at 70 °C for 12 hours. After completion, the resulting brownish solution was centrifuged to obtain a clear supernatant. Excess ethyl acetate was then added, leading to the formation of a white precipitate. The precipitate was collected and washed three times with diethyl ether.

**MC1**: Solid **L1** (28.54 mg, 0.135 mmol) was treated with DMSO solution of Pd(NO_3_)_2_ (18.0 mg, 0.0675 mmol). Yield = 40.2 mg, ⁓91%.

^1^H NMR (400 MHz, DMSO-*d*_6_): *δ* (ppm) *=* 9.81 (s, 12H), 8.37 (s, 12H), 8.33 (t, *J* = 8.1 Hz, 6H), 8.02 (s, 12H), 7.89 (d, *J* = 8.1 Hz, 12H). ^13^C NMR (176 MHz, DMSO-*d*_6_): *δ* (ppm) = 146.46, 144.77, 136.80, 130.22, 119.67, 112.94. ESI-MS (DMSO+CH_3_CN): m/z = 590.0657 for [**MC1**+3(NO_3_)]^3+^ (calc. 590.89) and 329.6454 for [**MC1**+1(NO_3_)]^5+^ (calc. 329.734).

**MC2**: A DMSO solution of Pd(NO_3_)_2_ (18.0 mg, 0.0676 mmol) was added to the solid **L2** (35.17 mg, 0.135 mmol). Yield = 48 mg, ⁓95%.

^1^H NMR (400 MHz, DMSO-*d*_6_): *δ* (ppm) *=* 9.46 (s, 12H), 8.73 (s, 12H), 8.20 (d, *J* = 4.6 Hz, 24H), 7.89 (d, *J* = 8.5 Hz, 12H), 7.77 (s, 12H). ^13^C NMR (176 MHz, DMSO-*d*_6_): *δ* (ppm) = 137.51, 134.71, 133.39, 131.54, 130.59, 129.55, 120.57, 120.22, 119.66. ESI-MS (DMSO+CH_3_CN): m/z = 1064.1582 for [**MC2**+4(NO_3_)]^2+^ (calc. 1064.545), 688.1104 for [**MC2**+3(NO_3_)]^3+^ (calc. 689.09) and 501.0912 for [**MC2**+2(NO_3_)]^4+^ (calc. 501.2725).

**MC3**:^[5]^ Solid **L3** (35.10 mg, 0.157 mmol) was treated with DMSO solution of Pd(NO_3_)_2_ (18.0 mg, 0.0675 mmol). Yield = 28.8 mg, ⁓90%.

^1^H NMR (400 MHz, DMSO-*d*_6_): *δ* (ppm) *=* 7.91 (s, 8H), 7.48 (s, 8H), 7.45 (d, *J* = 7.9 Hz, 4H), 7.37 (d, *J* = 7.7 Hz, 8H), 7.04 (s, 8H), 6.97 (s, 4H), 5.20 (s, 16H). ^13^C NMR (176 MHz, DMSO-*d*_6_): *δ* (ppm) = 138.99, 137.07, 129.88, 128.96, 128.52, 127.00, 122.42, 51.44. ESI-MS (DMSO+CH_3_CN): m/z = 1352.2754 for [**MC3**+1(NO_3_)]^+^ (calc. 1352.02), 645.1387 for [**MC3**+2(NO_3_)]^2+^ (calc. 645.01), 409.4388 for [**MC3**+3(NO_3_)]^3+^ (calc. 409.34) and 291.0824 for [**MC3**]^4+^ (calc. 291.505).

**MC4**:^[4]^ Solid **L4** (32.20 mg, 0.135 mmol) was mixed with a DMSO solution containing Pd(NO_3_)_2_ (20.96 mg, 0.0786 mmol). Yield = 42 mg, ⁓87%.

^1^H NMR (700 MHz, DMSO-*d*_6_): *δ* (ppm) = 10.42 (dd, *J* = 58.2, 16.1 Hz, 24H), 9.80 (d, *J* = 6.6 Hz, 12H), 9.68 (s, 12H), 8.71 (s, 12H), 8.59 (s, 1H), 8.03 (d, *J* = 4.0 Hz, 24H). ^13^C NMR (176 MHz, DMSO-*d*_6_): *δ* (ppm) = 173.39, 165.84, 155.07, 153.95, 150.90, 149.66, 140.22, 139.66, 128.67, 128.71, 125.71, 123.30, 123.21. ESI-MS (DMSO+CH_3_CN): 616.0404 for [**MC4** + 6(NO_3_)]^6+^ (calc. 914.91)

**MC5**:^[6]^ Solid **L5** (31.56 mg, 0.150 mmol) was treated with DMSO solution of Pd(NO_3_)_2_ (20.0 mg, 0.0750 mmol). Yield = 43.8 mg, ⁓89%.

^1^H NMR (700 MHz, DMSO-*d*_6_): *δ* (ppm) *=* 9.29 (s, 12H), 8.39 (s, 6H), 8.15 (s, 12H), 7.77 (d, *J* = 8.3 Hz, 12H), 7.74 (s, 18H). ^13^C NMR (176 MHz, DMSO-*d*_6_): *δ* (ppm) = 137.16 (s), 136.75 (s), 132.17 (s), 129.69 (s), 120.73 (s), 120.24 (s), 114.92 (s). ESI-MS (DMSO+CH_3_CN): m/z = 914.0594 for [**MC5**+4(NO_3_)]^2+^ (calc. 914.10572), 588.0367 for [**MC5**+3(NO_3_)]^3+^ (calc. 588.74120).


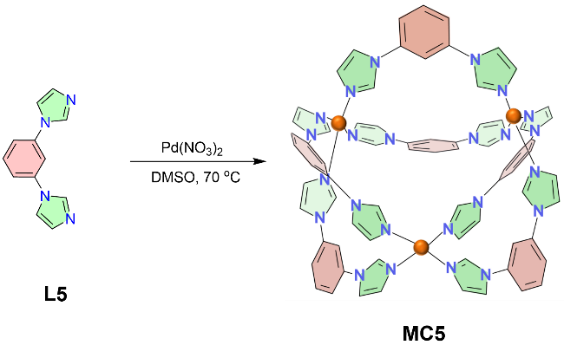


**Scheme S6**. Synthesis of the self-assembly of the cage **MC5**.


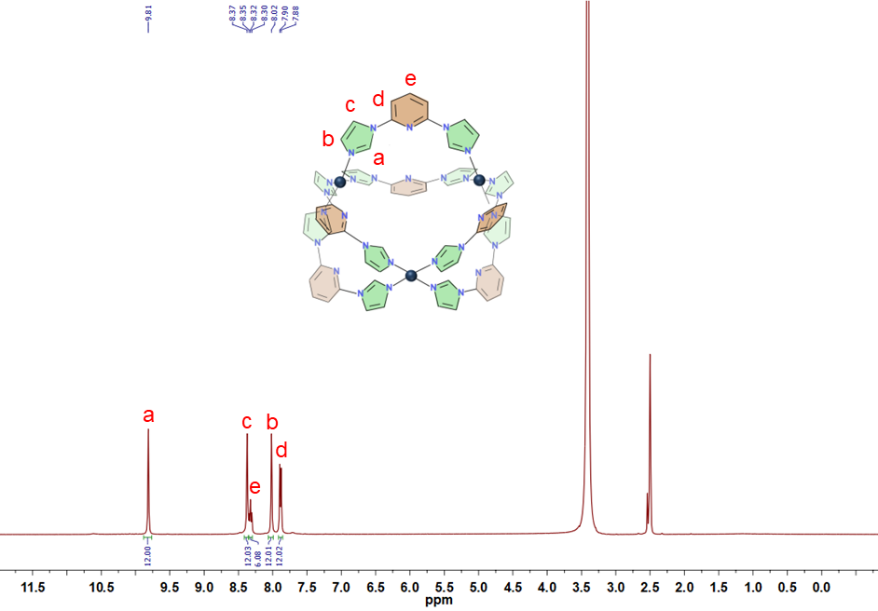


**Figure S11**. ^1^H NMR spectrum of **MC1** in DMSO-*d*_6_ (400 MHz, 298 K).


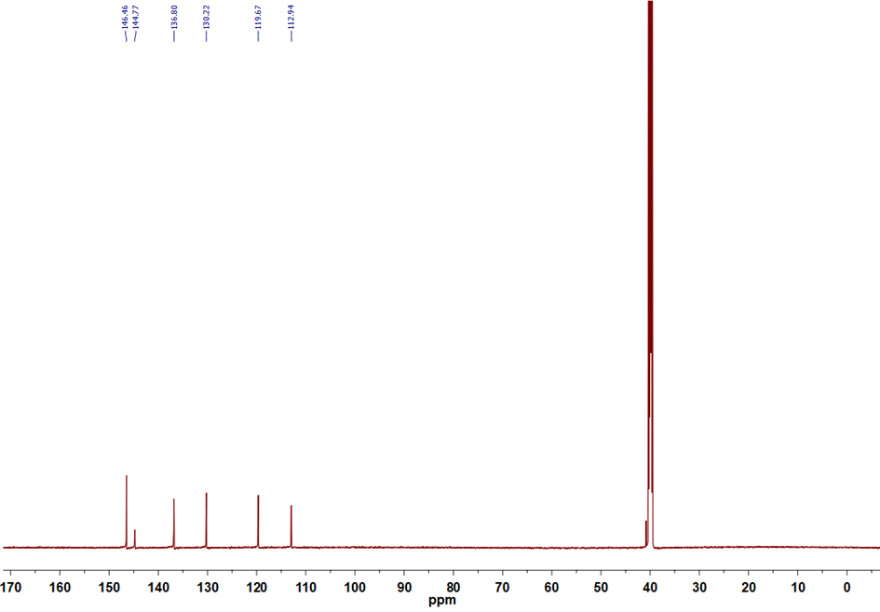


**Figure S12**. ^13^C NMR spectrum of **MC1** in DMSO-*d*_6_ (176 MHz, 298 K).


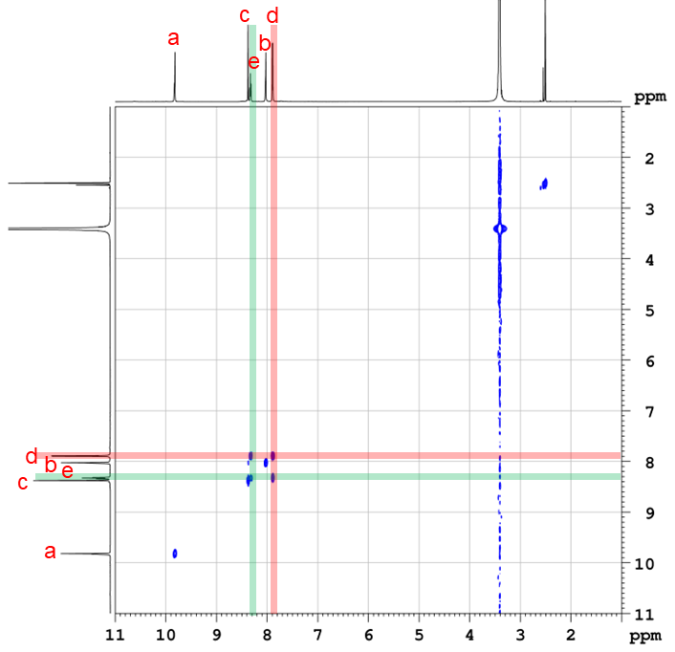


**Figure S13**. ^1^H-^1^H COSY spectrum of cage **MC1** (700 MHz, 298K, DMSO-*d*_6_).


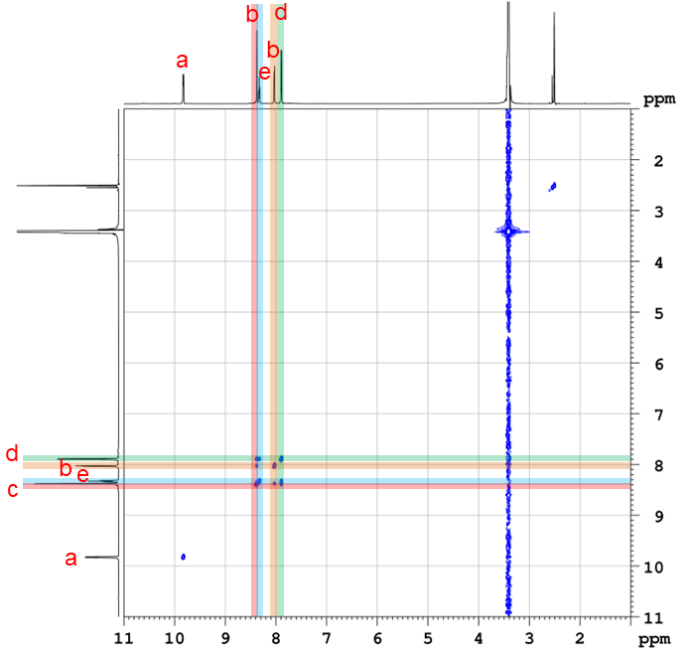


**Figure S14**. ^1^H-^1^H NOESY spectrum of **MC1** in DMSO-*d*_6_ (700 MHz, 298 K).


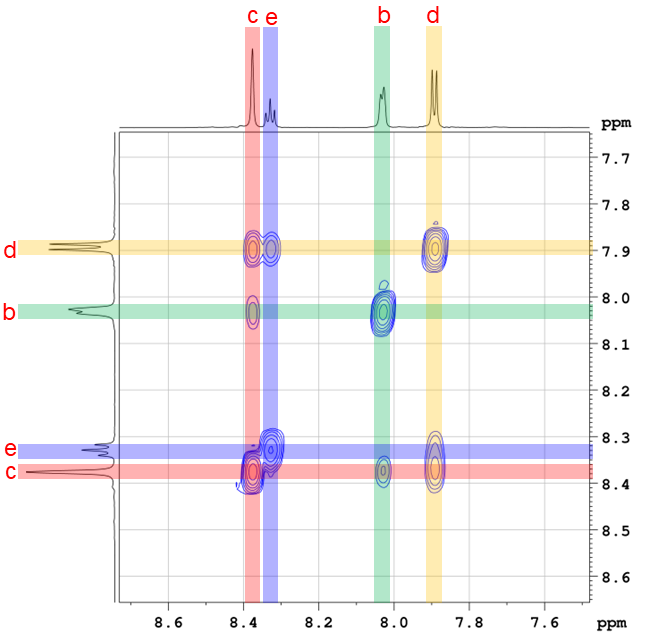


**Figure S15**. Partial ^1^H-^1^H NOESY spectrum of **MC1** in DMSO-*d*_6_ (700 MHz, 298 K).

**
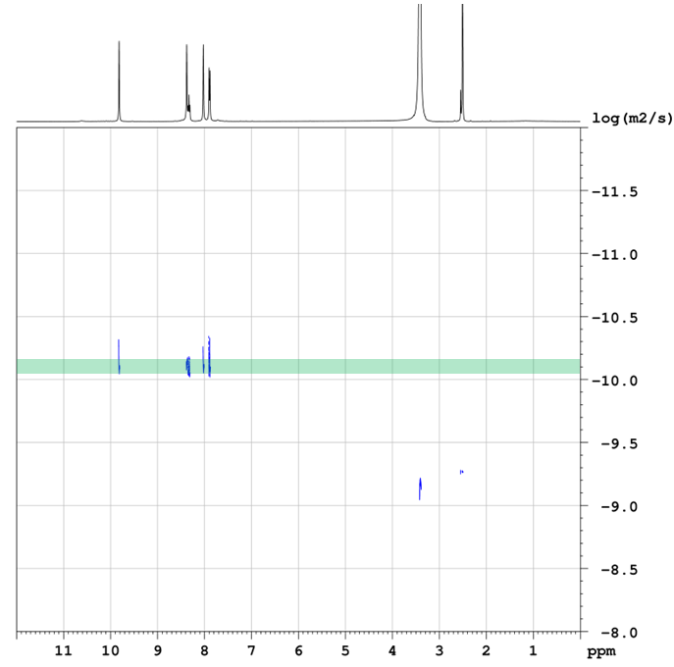
**

**Figure S16**. ^1^H DOSY spectrum of **MC1** in DMSO-*d*_6_ (400 MHz, 298 K).


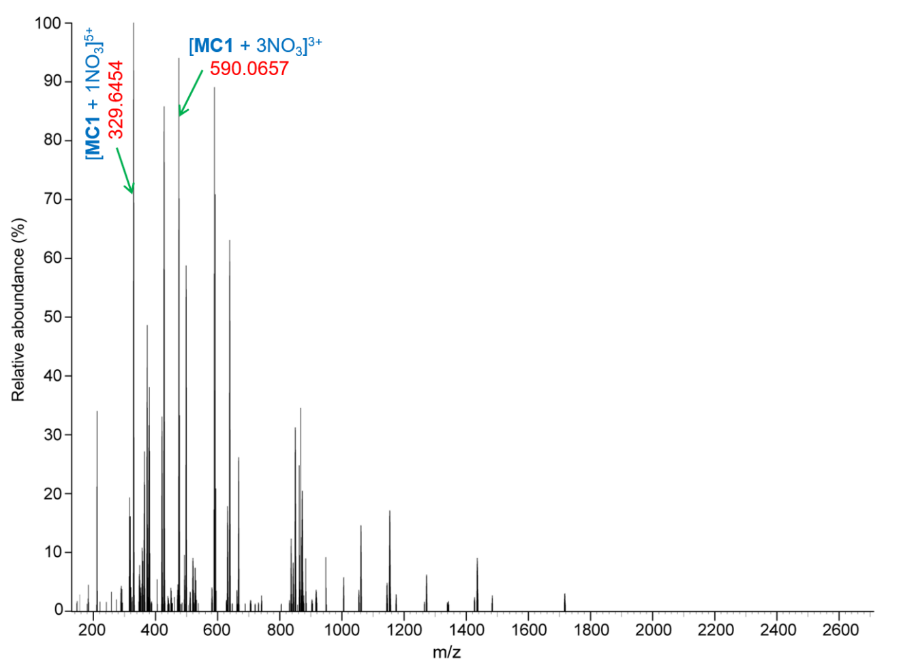


**Figure S17**. ESI-MS spectrum of **MC1** in DMSO and CH_3_CN mixture.


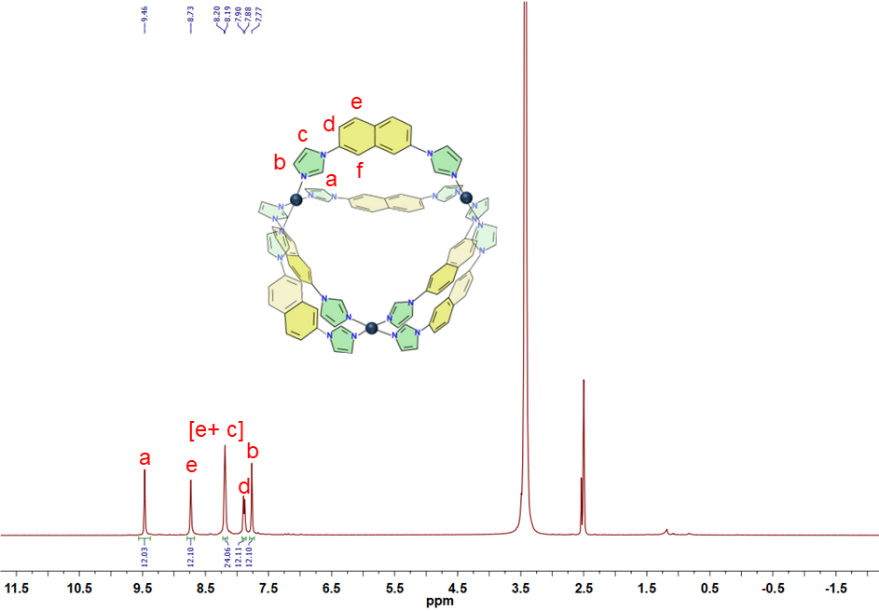


**Figure S18**. ^1^H NMR spectrum of **MC2** in DMSO-*d*_6_ (700 MHz, 298 K).


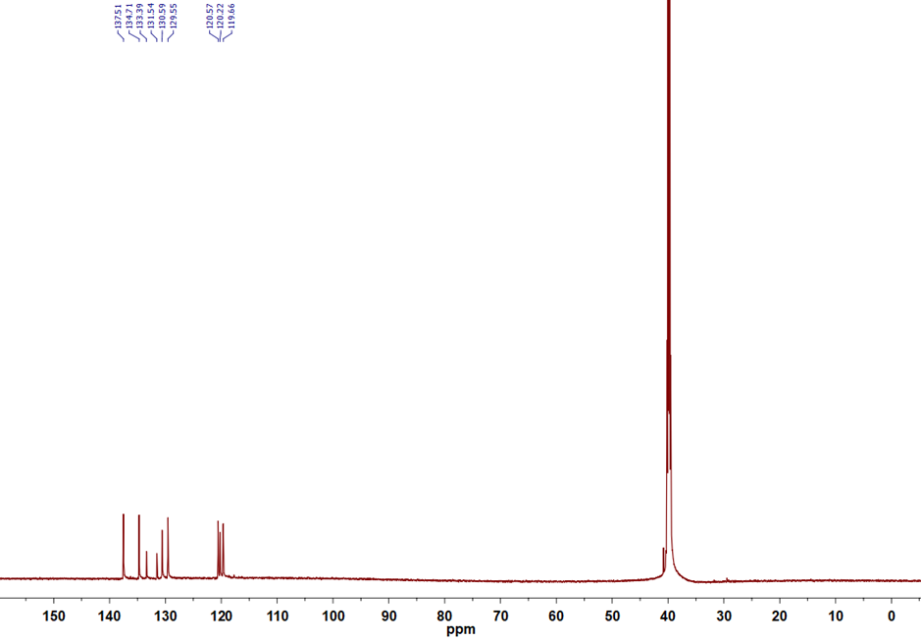


**Figure S19**. ^13^C NMR spectrum of **MC2** in DMSO-*d*_6_ (176 MHz, 298 K).


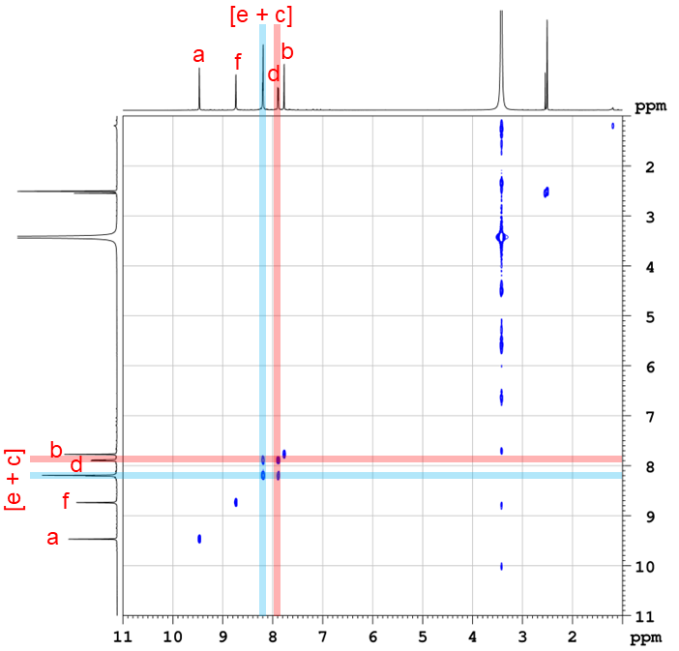


**Figure S20**. ^1^H-^1^H COSY spectrum of **MC2** in DMSO-*d*_6_ (700 MHz, 298 K).

**
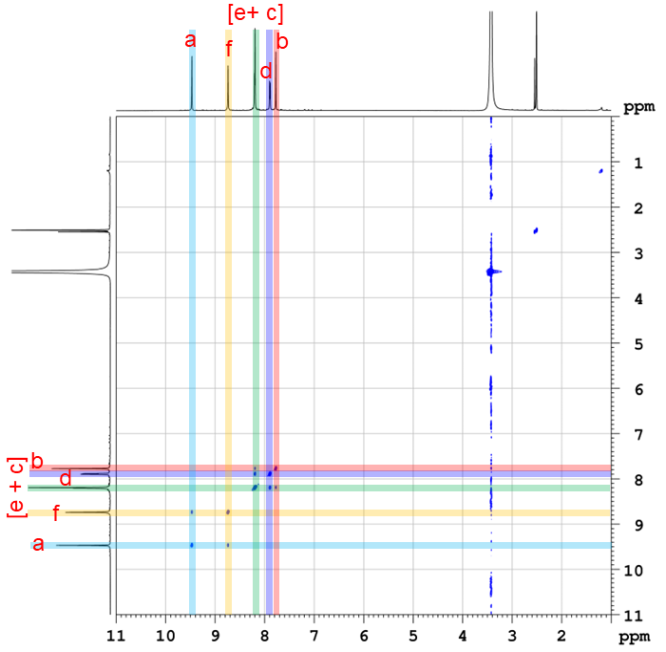
**

**Figure S21**. ^1^H-^1^H NOESY spectrum of **MC2** in DMSO-*d*_6_ (700 MHz, 298 K).

**
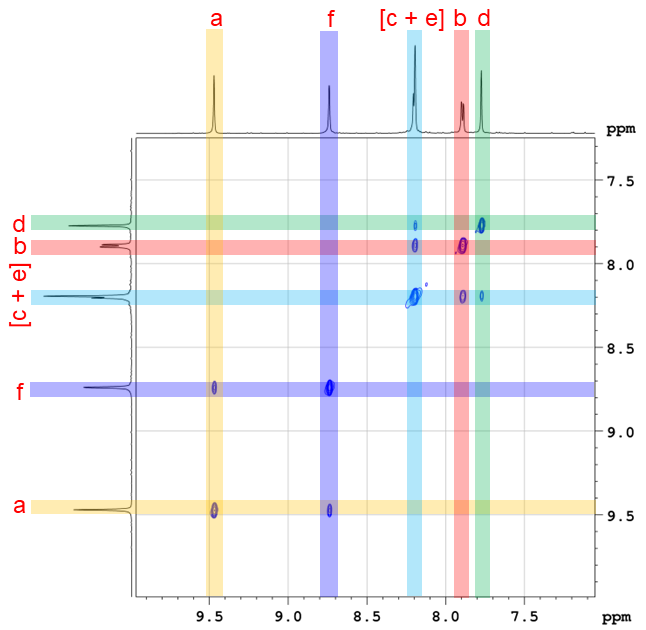
**

**Figure S22**. Partial ^1^H-^1^H NOESY spectrum of **MC2** in DMSO-*d*_6_ (700 MHz, 298 K).


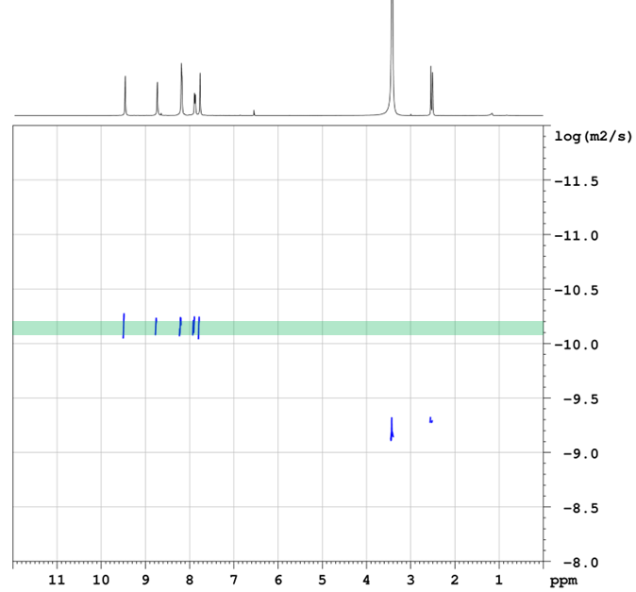


**Figure S23**. ^1^H DOSY NMR spectrum of **MC2** in DMSO-*d*_6_ (400 MHz, 298 K).


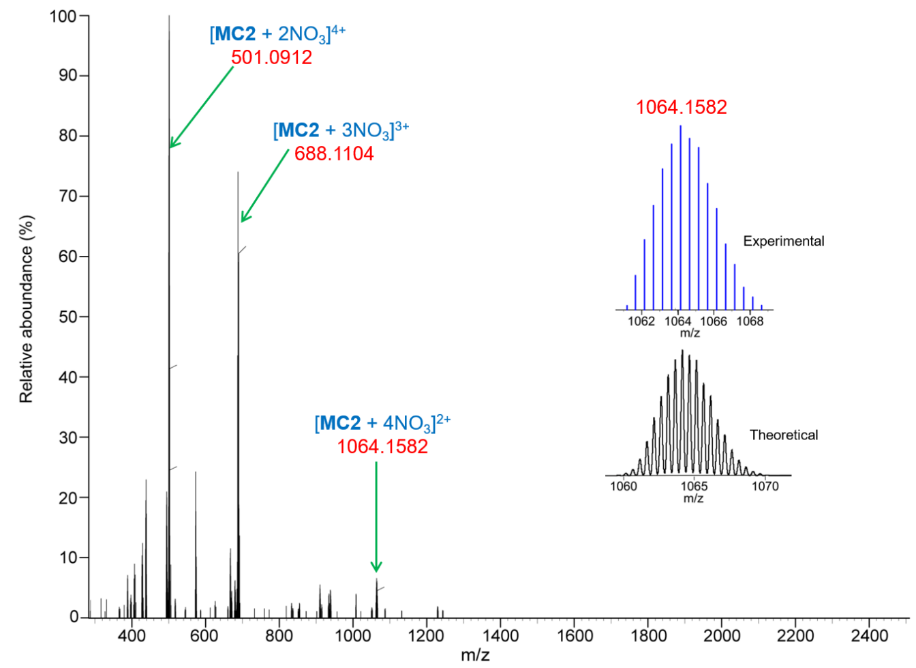


**Figure S24**. ESI-MS spectrum of **MC2** in DMSO and CH_3_CN mixture.


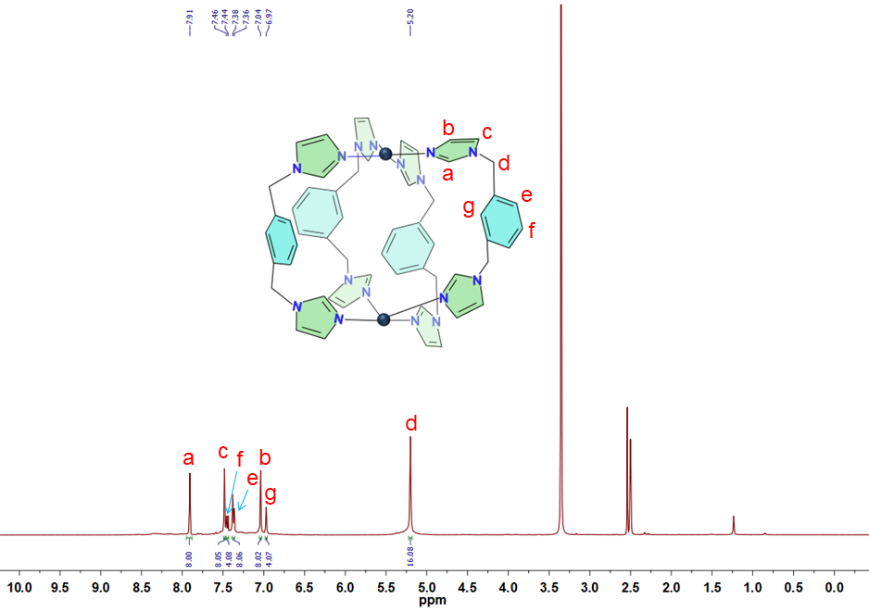


**Figure S25**. ^1^H NMR spectrum of **MC3** in DMSO-*d*_6_ (400 MHz, 298 K).


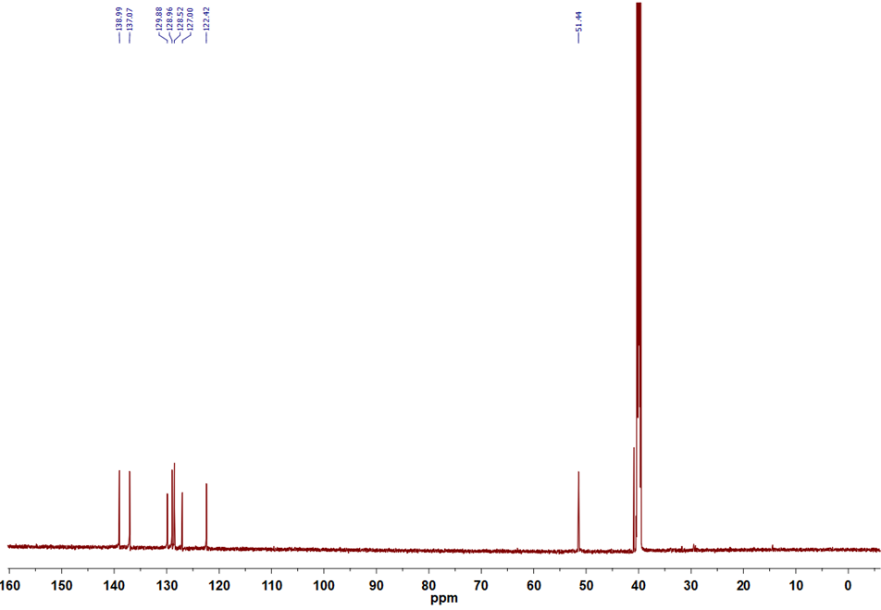


**Figure S26**. ^13^C NMR spectrum of **MC3** in DMSO-*d*_6_ (176 MHz, 298 K).


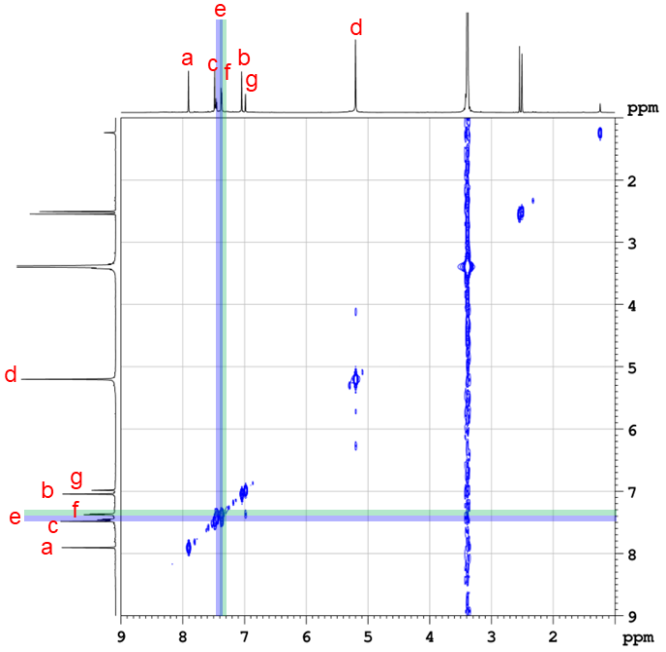


**Figure S27**. ^1^H-^1^H COSY spectrum of **MC3** in DMSO-*d*_6_ (700 MHz, 298 K).


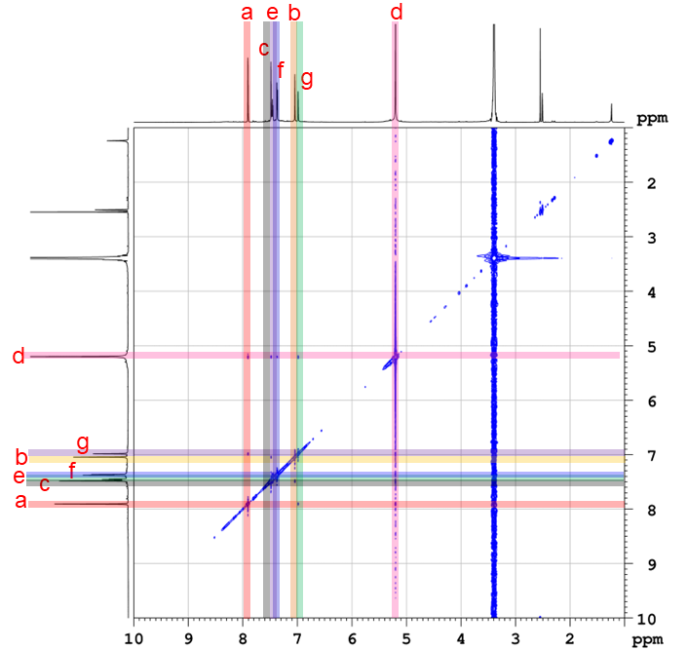


**Figure S28**. ^1^H-^1^H NOESY spectrum of **MC3** in DMSO-*d*_6_ (700 MHz, 298 K).


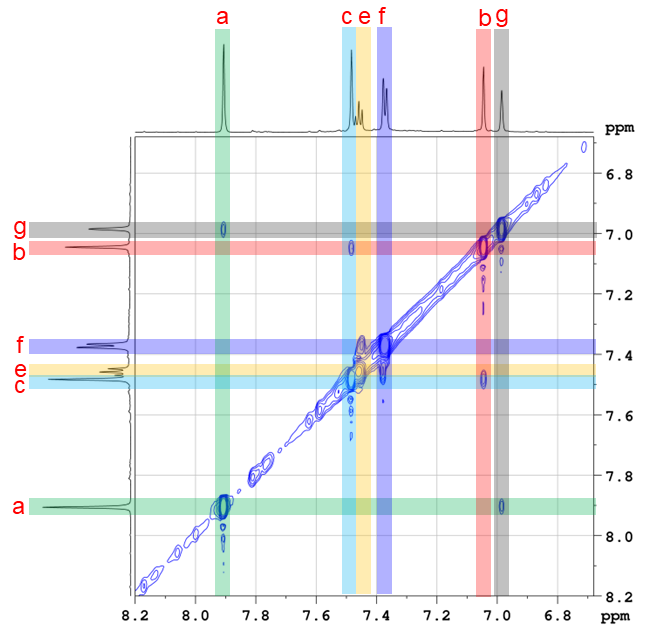


**Figure S29**. Partial ^1^H-^1^H NOESY spectrum of **MC3** in DMSO-*d*_6_ (700 MHz, 298 K).


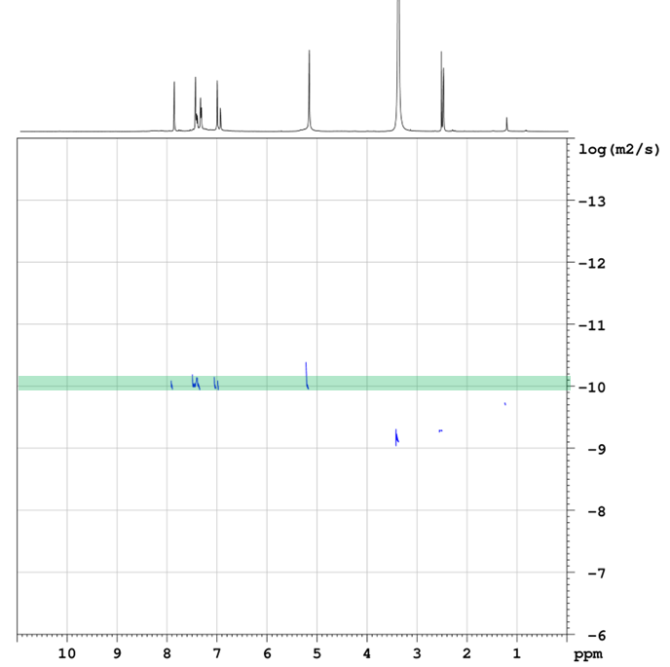


**Figure S30**. ^1^H DOSY NMR spectrum of **MC3** in DMSO-*d*_6_ (400 MHz, 298 K).


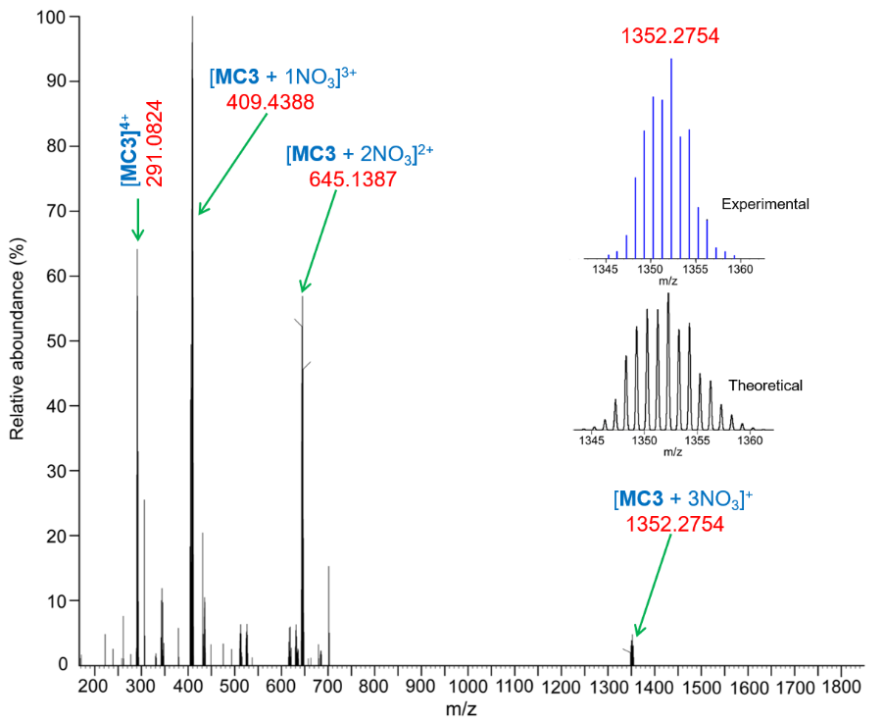


**Figure S31**. ESI-MS spectrum of **MC3** in DMSO and CH_3_CN mixture.


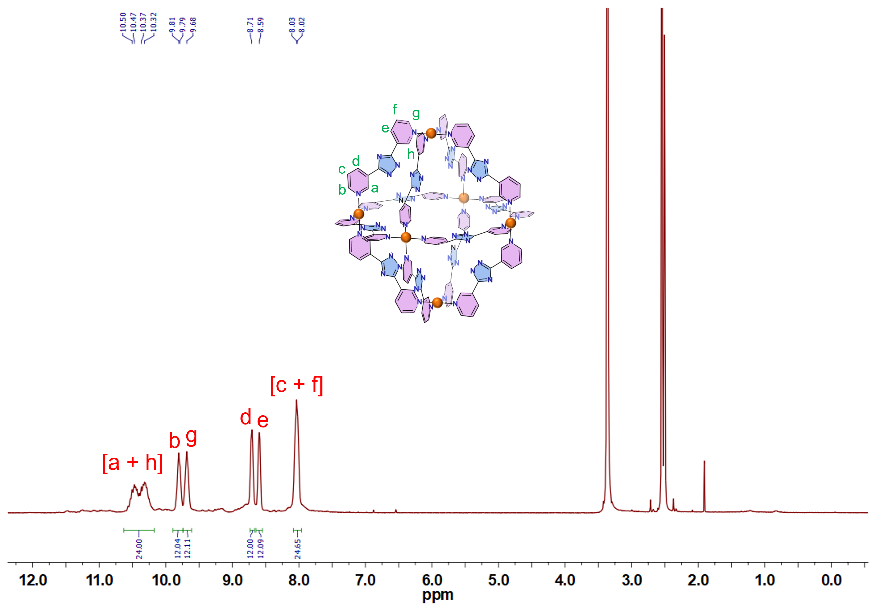
**Figure S32**. ^1^H NMR spectrum of **MC4** in DMSO-*d*_6_ (700 MHz, 298 K).


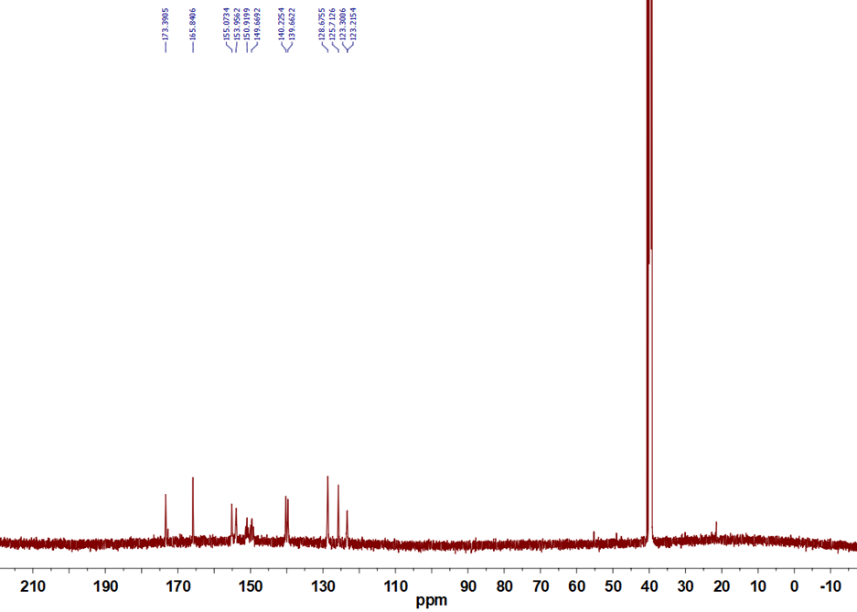


**Figure S33**. ^13^C NMR spectrum of **MC4** in DMSO-*d*_6_ (176 MHz, 298 K).


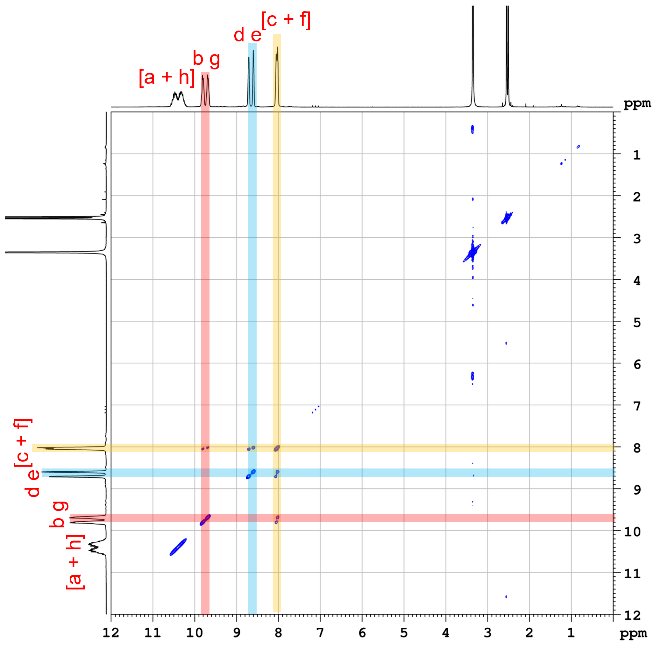


**Figure S34**. ^1^H-^1^H COSY spectrum of **MC4** in DMSO-*d*_6_ (700 MHz, 298 K).


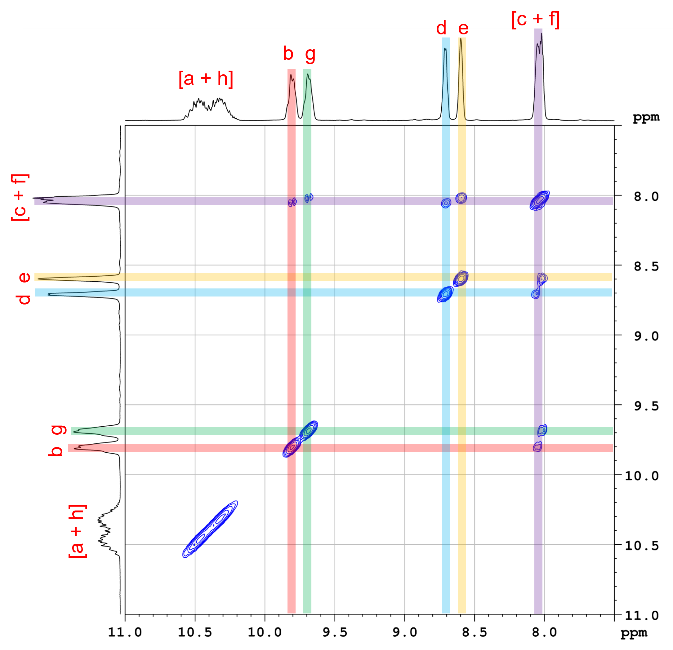


**Figure S35**. Partial ^1^H-^1^H COSY spectrum of **MC4** in DMSO-*d*_6_ (700 MHz, 298 K).


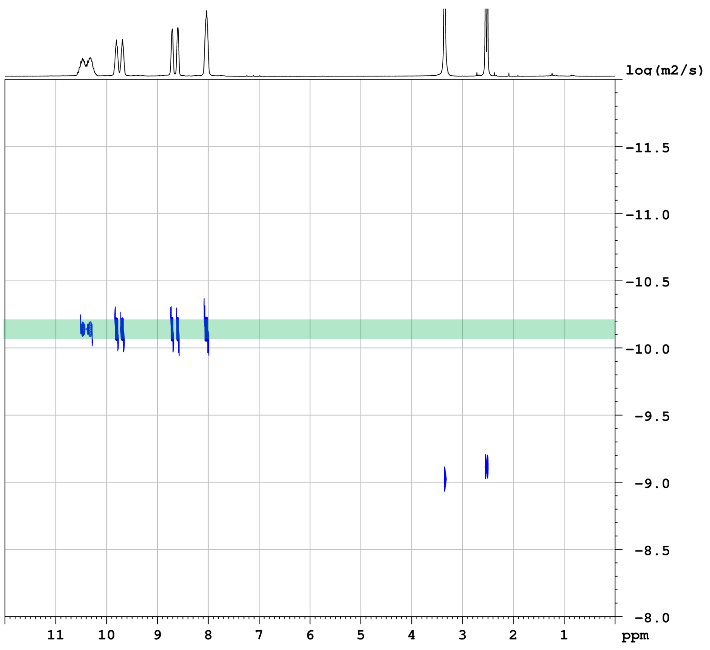


**Figure S36**. ^1^H DOSY NMR spectrum of **MC4** in DMSO-*d*_6_ (400 MHz, 298 K).


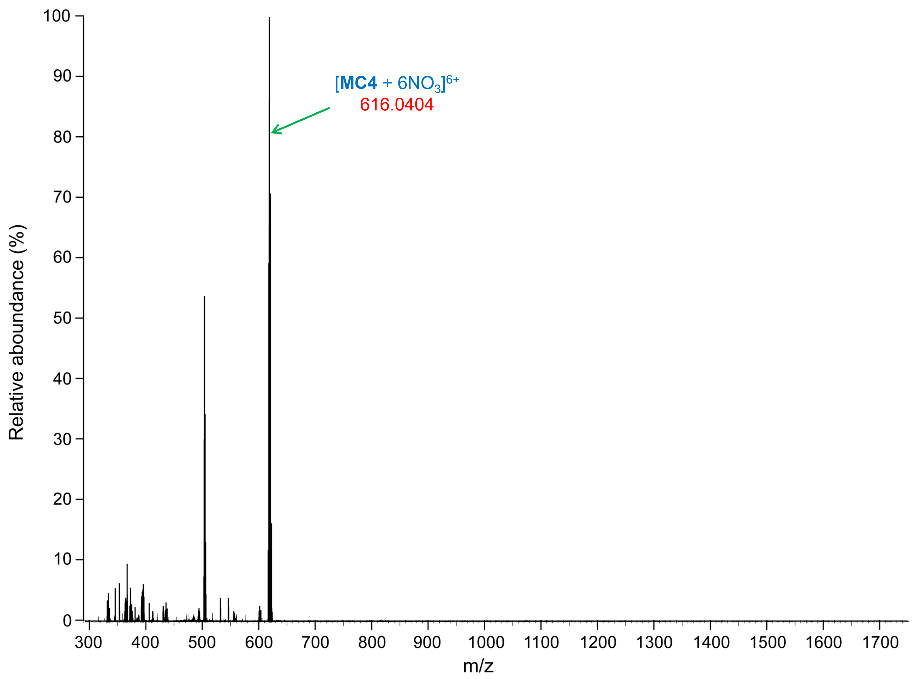


**Figure S37**. ESI-MS spectrum of **MC4** in DMSO and CH_3_CN mixture.


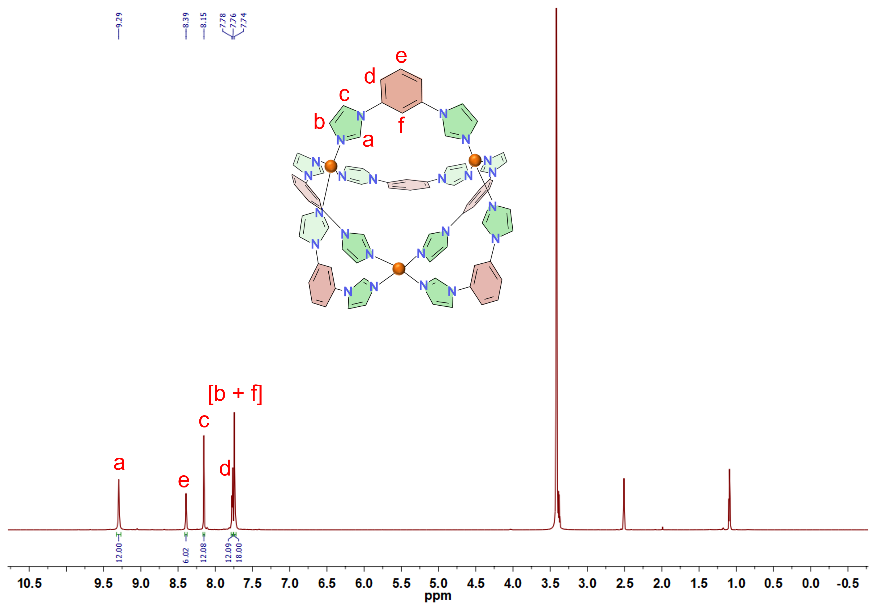


**Figure S38**. ^1^H NMR spectrum of **MC5** in DMSO-*d*_6_ (400 MHz, 298 K).


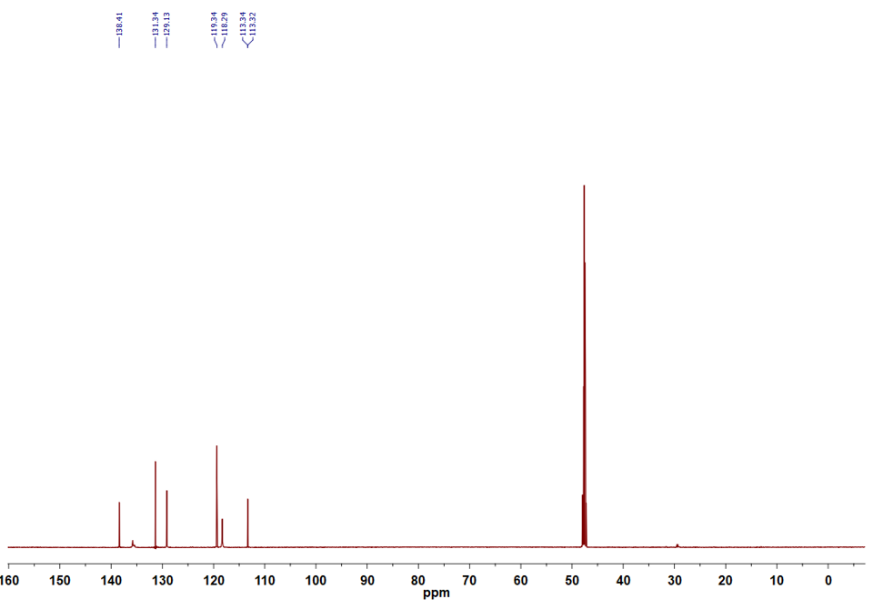


**Figure S39**. ^13^C NMR spectrum of **MC5** in DMSO-*d*_6_ (176 MHz, 298 K).


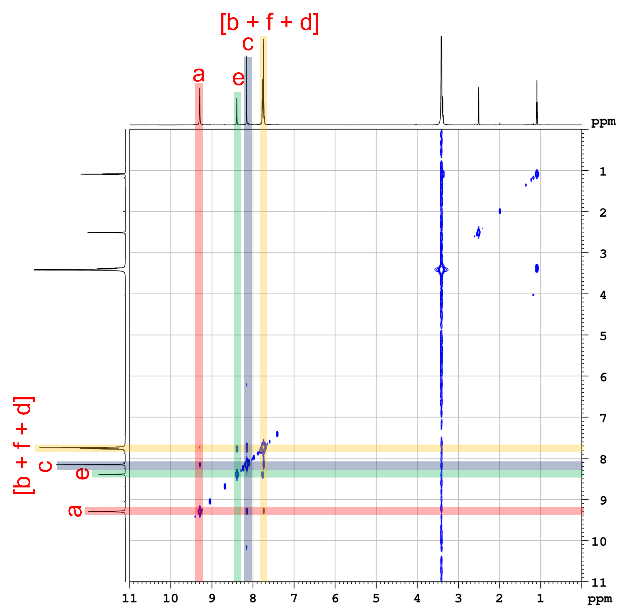


**Figure S40**. ^1^H-^1^H COSY spectrum of **MC5** in DMSO-*d*_6_ (700 MHz, 298 K).


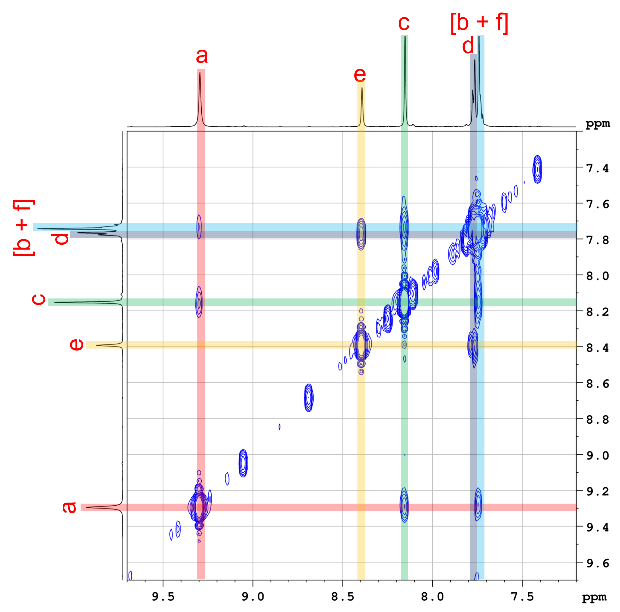


**Figure S41**. Partial ^1^H-^1^H COSY spectrum of **MC5** in DMSO-*d*_6_ (700 MHz, 298 K).


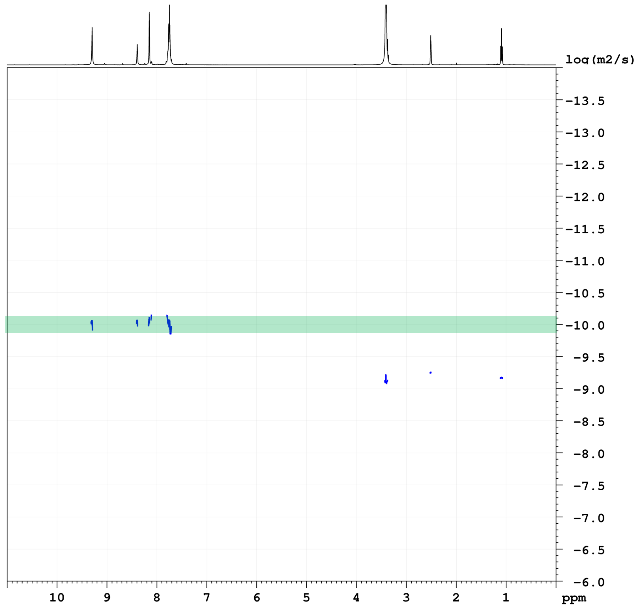


**Figure S42**. ^1^H DOSY NMR spectrum of **MC5** in DMSO-*d*_6_ (400 MHz, 298 K).


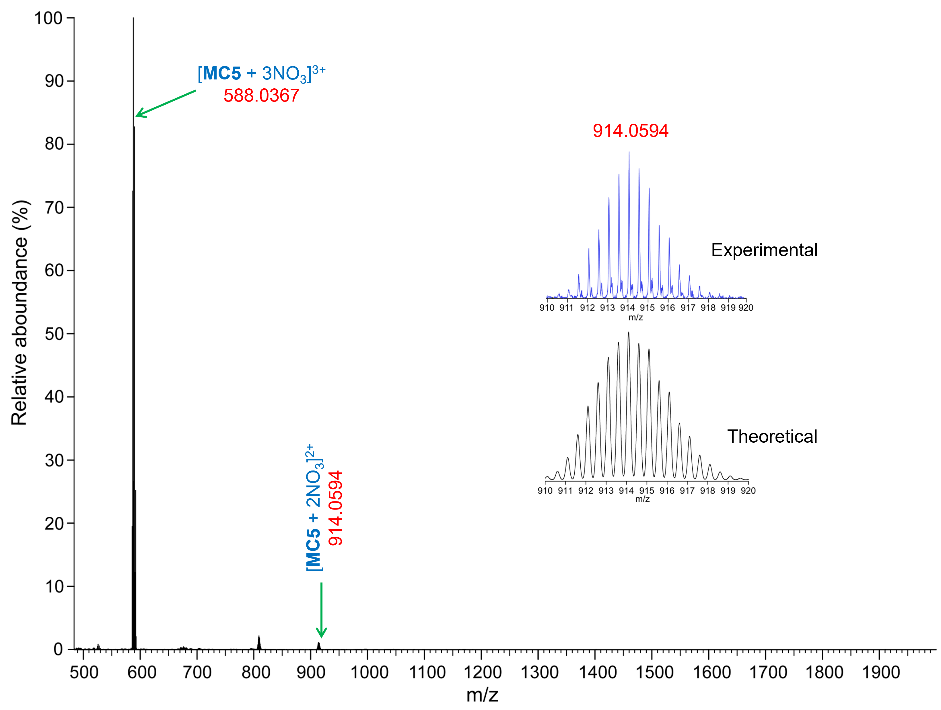


**Figure S43**. ESI-MS spectrum of **MC5** in DMSO and CH_3_CN mixture.

**3**. **X-ray Data Collection and Structure Refinement**

The structural analysis of three coordination cages (**MC1** and **MC2**) were performed using single crystal X-ray crystallography. Suitable single crystals for X-ray diffraction were selected, coated with crystal oil, and mounted under standard conditions. These crystals were assigned the accession codes CCDC 2433497 and 2433498 for **MC1** and **MC2** respectively. X-ray diffraction was conducted on a Rigaku Super-Nova dual diffractometer equipped with fine-focus Cu-Kα radiation (λ = 1.54184 Å) and multilayer optics with aluminum filtration. A PILATUS200K detector was used at 100 K. Intensity data were collected, corrected, and absorption corrections were applied using the multi-scan method with SCALE3 ABSPACK in CrysAlisPro 50 software. The single-crystal X-ray diffraction data were processed using Olex2 software^[7]^, and structural refinement was conducted via least-squares minimization in SHELXL^[8]^. Anisotropic refinement was applied to all non-hydrogen atoms. Hydrogen atoms, except those in water molecules due to their elongated O–H bond lengths, were placed in idealized geometric positions and refined using a riding model. The electron density corresponding to other disordered solvent molecules was squeezed. Despite multiple attempts, the precise positions of hydrogen atoms in water molecules could not be accurately determined.

**Table S1**: Crystallographic table of **MC1**.

| **Parameters** | **MC1** |
| --- | --- |
| Formula | C_66_H_54_F_20_N_30_O_3_P_8_Pd_3_ |
| *Fw* | 2262.35 |
| Crystal system | Hexagonal |
| Space group | *P*6_3_*/mmc* |
| Crystal colour and shape | Light grey and Block |
| *a*/Å | 16.2923(2) |
| *b*/Å | 16.2923(2) |
| *c*/Å | 32.3162(3) |
| *α*/° | 90 |
| *β*/° | 90 |
| *γ*/° | 120 |
| *V*/Å^3^ | 7428.75(19) |
| *Z* | 2 |
| *D*_c_/g cm^−3^ | 1.011 |
| *μ* Cu K_α_/mm^−1^ | 1.54184 |
| F000 | 2244.0 |
| *T*/K | 99.99(10) |
| *θ* max. | 77.469 |
| Total no. of reflections | 32088 |
| Independent reflections | 2932 |
| Observed reflections | 2794 |
| Parameters refined | 117 |
| *R*_1_, *I* > 2*σ*(*I*) | 0.0739 |
| w*R*_2_, *I* > 2*σ*(*I*) | 0.2301 |
| GOF (*F*^2^) | 1.096 |
| CCDC no. | 2433497 |

**Table S2**: Crystallographic data and refinement parameters for **MC2**

| **Parameters** | **MC2** |
| --- | --- |
| Formula | C_96_ H_72_N_26_O_6_Pd_3_ |
| *Fw* | 2004.99 |
| Crystal system | Orthorhombic |
| Space group | *Pnma* |
| Crystal colour and shape | Colourless and Block |
| *a*/Å | 44.8997(12) |
| *b*/Å | 27.7905(7) |
| *c*/Å | 12.3936(3) |
| *α*/° | 90 |
| *β*/° | 90 |
| *γ*/° | 90 |
| *V*/Å^3^ | 15464.6(7) |
| *Z* | 4 |
| *D*_c_/g cm^−3^ | 0.861 |
| *μ* Cu K_α_/mm^−1^ | 1.54184 |
| F000 | 4064.0 |
| *T*/K | 100.00(10) |
| *θ* max. | 51.554 |
| Total no. of reflections | 33825 |
| Independent reflections | 8472 |
| Observed reflections | 5425 |
| Parameters refined | 574 |
| *R*_1_, *I* > 2*σ*(*I*) | 0.0696 |
| w*R*_2_, *I* > 2*σ*(*I*) | 0.2354 |
| GOF (*F*^2^) | 1.131 |
| CCDC no. | 2433498 |

**4**. **Gas Sorption Isotherm**


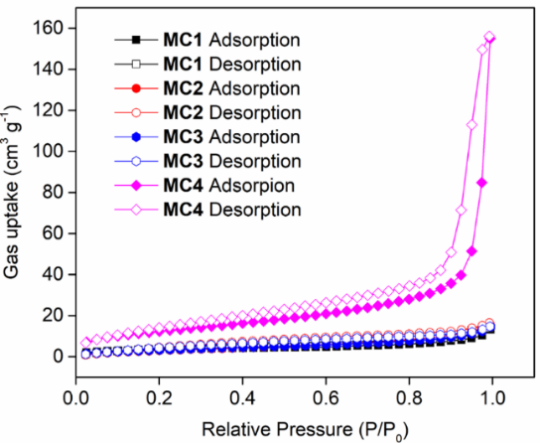


**Figure S44**. Nitrogen sorption isotherms of all four cages


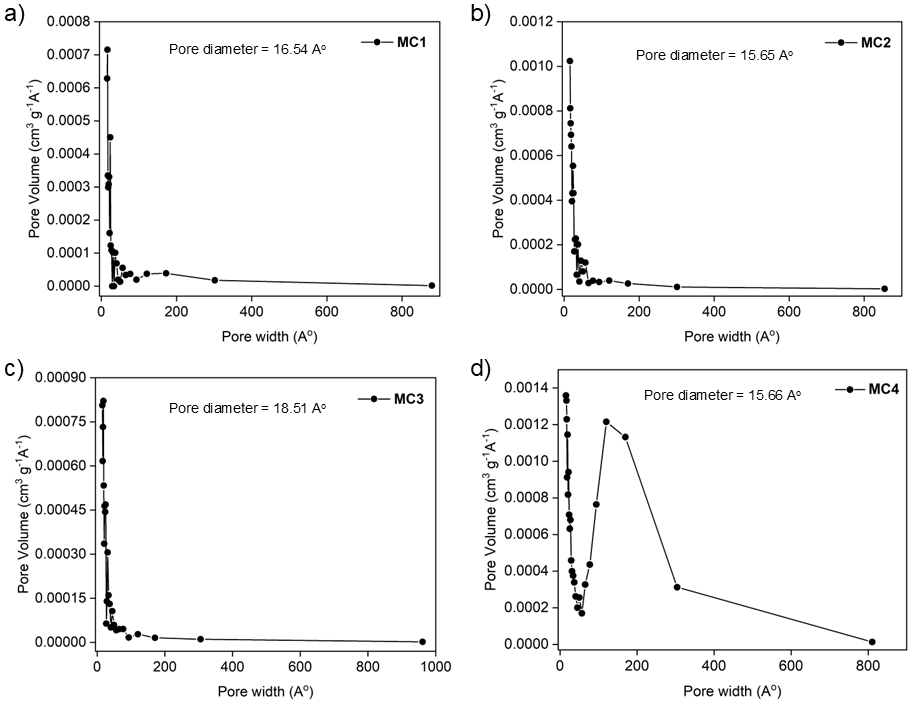


**Figure S45**. Pore size distributions of coordination cages (a) **MC1**, (b) **MC2**, (c) **MC3**, and (d) **MC4**.

**Table S3**. Summary of surface area, pore size and pore volume

| Cage | Surface Area  (m^2^ g) | Pore Volume  (cm^3^ g^‒1^) | Pore Diameter  (Å) |
| --- | --- | --- | --- |
| **MC1** | 12.92 | 0.016 | 16.54 |
| **MC2** | 12.55 | 0.020 | 15.65 |
| **MC3** | 13.19 | 0.017 | 18.51 |
| **MC4** | 45.409 | 0.223 | 15.66 |

**5**. **FE-SEM Image**


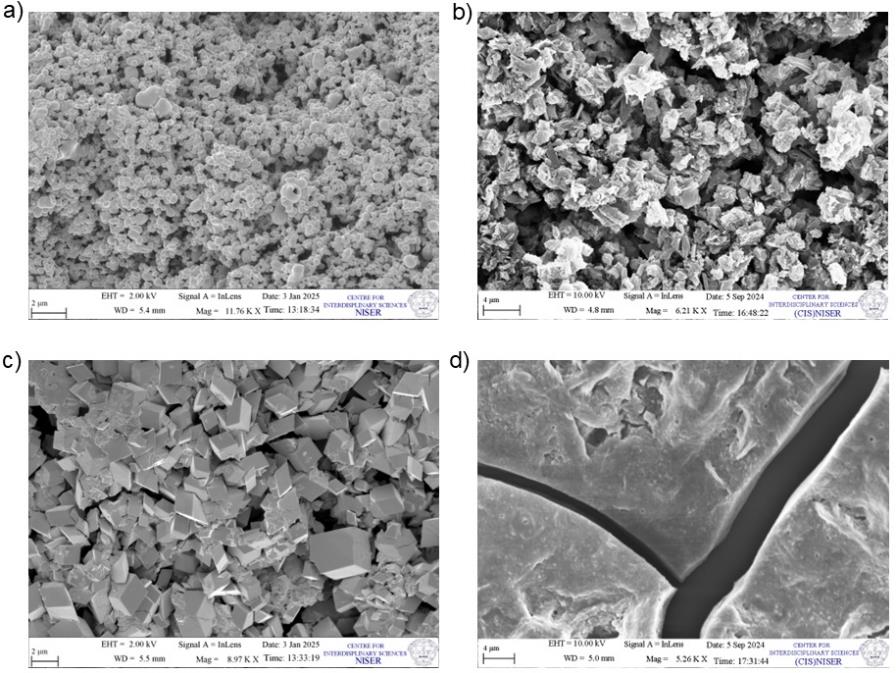


**Figure S46**. FE-SEM image of the cages (a) **MC1**, (b) **MC2**, (c) **MC3**, and (d) **MC4**.

**6**. **EDAX Profile for Cages**


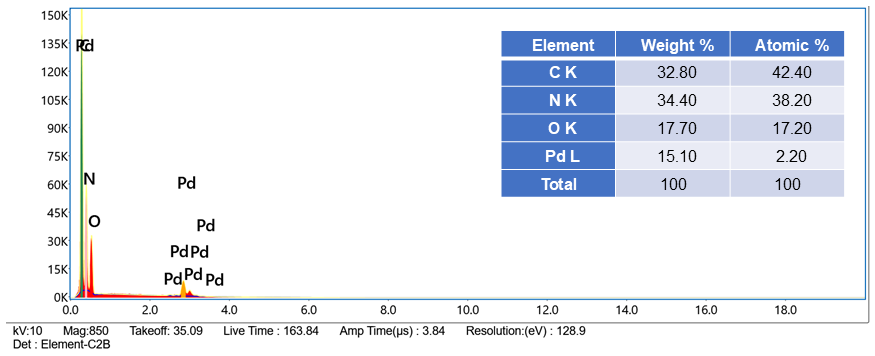


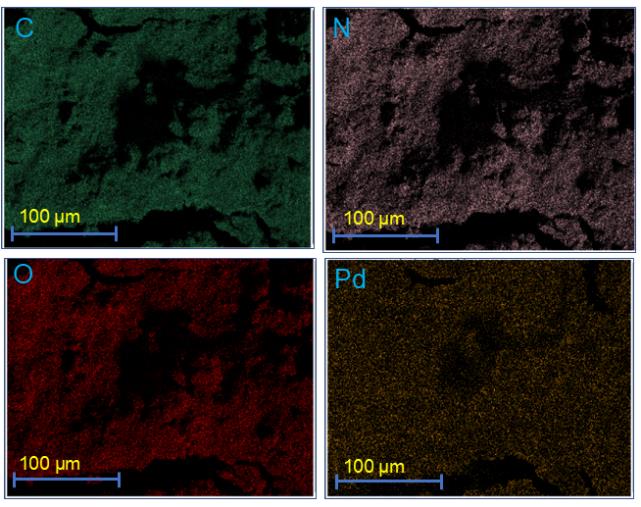


**Figure S47**. FE-SEM-based EDAX elemental analysis of **MC1** (top) and corresponding EDS elemental mapping of the **MC1** cage (bottom).


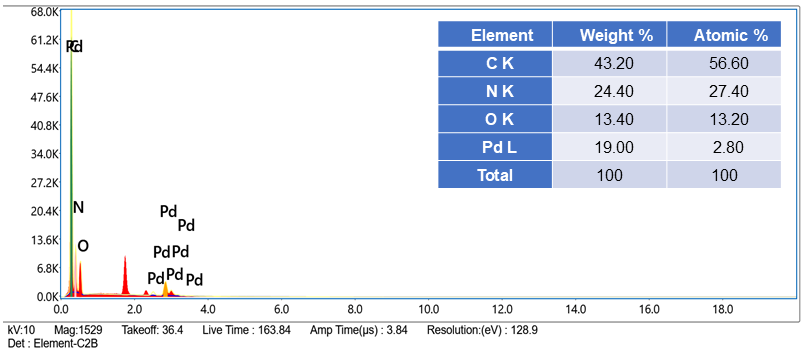


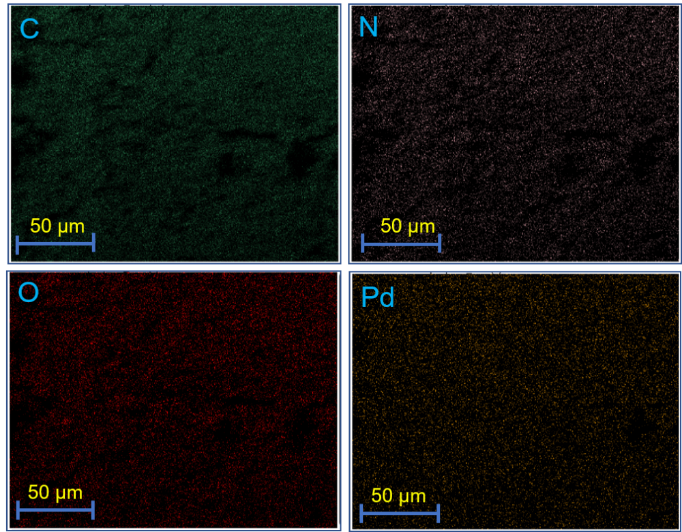


**Figure S48**. EDAX elemental analysis of **MC2** (top) and the corresponding EDS elemental mapping images (bottom).


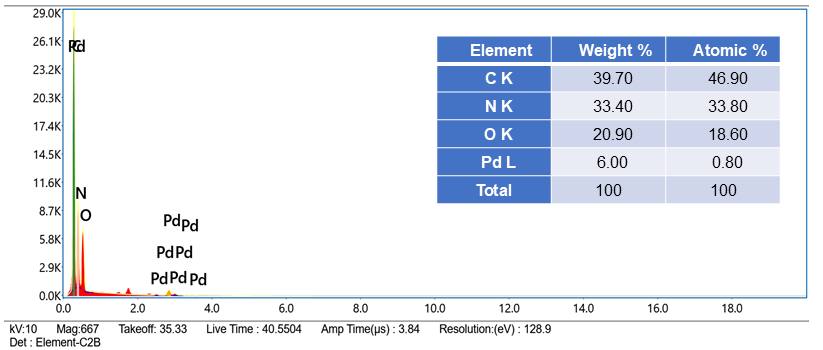


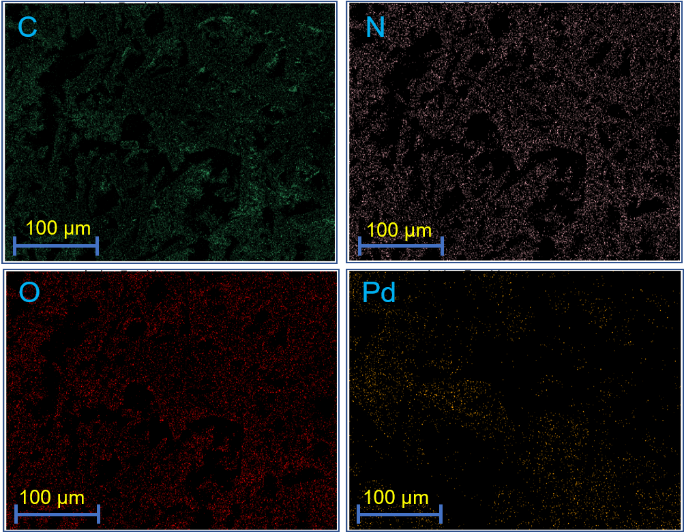


**Figure S49**. EDAX elemental analysis of **MC3** (top) and the corresponding EDS elemental mapping images (bottom).


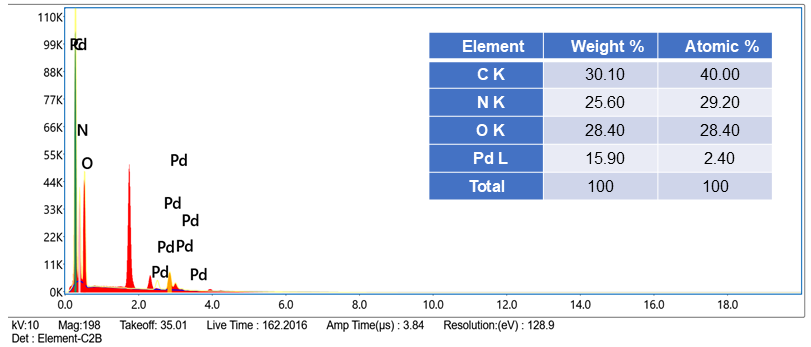


**
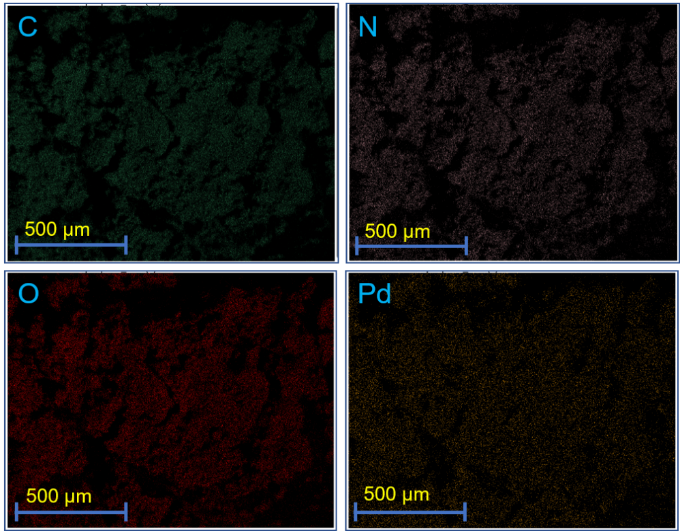
**

**Figure S50**. EDAX elemental analysis of **MC4** (top) and the corresponding EDS elemental mapping images (bottom).

**7**. **Iodine Vapour Adsorption Experiment**

**Vapor Phase Iodine Uptake Studies**:

The adsorption of iodine vapor onto different adsorbents was measured using a gravimetric approach.^[9]^ Before the adsorption process, each adsorbent sample underwent pre-activation by heating at 100 °C for 24 h. After this treatment, 10 mg of the activated adsorbent was precisely weighed and placed in small vials. These vials were then transferred to a sealed container maintained at 75 °C, with solid iodine present at the bottom. At specific time intervals, the vial was taken out, cooled to room temperature, and weighed to track the increase in mass due to iodine uptake. Then the vial was returned back to oven for continue the adsorption experiment. This cycle of heating, cooling, and weighing was repeated until the mass stabilized, signifying that adsorption equilibrium had been achieved.

$$w_{t}=\frac{w_{2}-w_{1}}{w_{1}}$$

Where, *w*_t_ (g g^−1^) represents the iodine adsorption capacity per gram of adsorbent at time t (minutes). *w*_2_ (mg) and *w*_1_ (mg) are the initial and final weight of the vails containing adsorbents respectively.

**Kinetic Models of Iodine Adsorption**:

The adsorption kinetic was investigated by fitting the experimental data to both pseudo-first-order (Eq.1) and pseudo-second-order (Eq.2) kinetic models.^[10]^ The linear forms of these models are expressed as follows:

$\ln\left( q_{e}-q_{t} \right)=\ln q_{e}-k_{1}t$………………………… (Eq.1)

$\frac{t}{q_{t}}=\frac{1}{k_{2}q_{e}^{2}}+\frac{t}{q_{e}}$................................................ (Eq.2)

In these equations, *q*_e_ and *q*_t_ (g g^−1^) denote the equilibrium adsorption capacity and the adsorption capacity at time *t* (minutes), respectively. *k*_1_ (min^−1^) represents the rate constant of the pseudo-first-order model, while *k*_2_ (g g^−1^ min^−1^) represents the rate constant of the pseudo-second-order model.


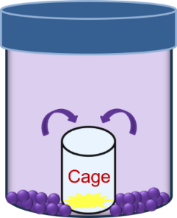


**Figure S51**. Experimental setup for iodine adsorption measurements utilizing gravimetric analysis.

**Retention Ability Test**:

To assess the long-term stability of iodine adsorption on the cage material (I_2_@**MC**), a measured amount of the iodine-loaded sample was placed in an open glass vial. This vial was then positioned inside a larger, empty bottle and kept at room temperature and atmospheric pressure. After 24 h, the mass of the vial containing the iodine-adsorbed material was recorded. The vial was then returned to the larger bottle, which remained undisturbed for another seven days. The large bottle was opened to further continue the experiment for next seven days.


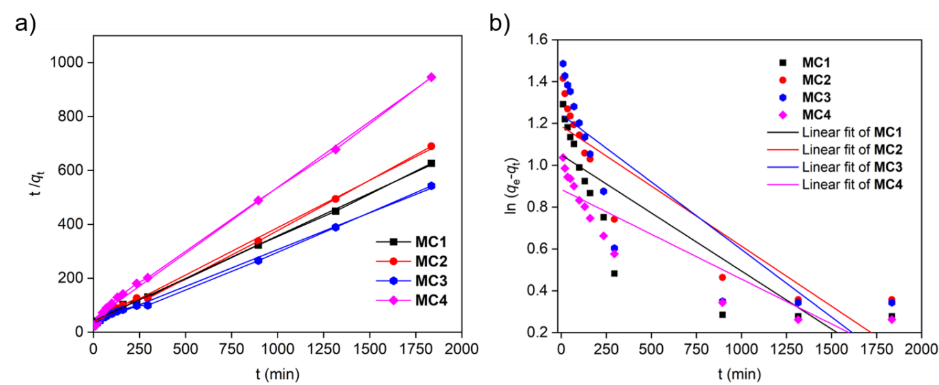


**Figure S52**. Kinetic modelling of iodine vapor adsorption using the (a) Pseudo-second-order model (b) Pseudo-first-order model at 75 °C.

**Table S4**. Kinetic parameters for the adsorption of iodine by the cages

| Cage | Pseudo-first order Kinetics | | | Pseudo-second order Kinetics | | |
| --- | --- | --- | --- | --- | --- | --- |
|  | R^2^ | *q*_e_ | *k*_1_  (g g^‒1^ h^‒1^) | R^2^ | *q*_e_ | *k*_2_  (g g^‒1^ h^‒1^) |
| **MC1** | 0.70 | 2.85 | 0.0333 | 0.99 | 2.84 | 0.2046 |
| **MC2** | 0.79 | 3.28 | 0.0344 | 0.99 | 3.16 | 0.1452 |
| **MC3** | 0.71 | 3.46 | 0.0387 | 0.99 | 3.66 | 0.1332 |
| **MC4** | 0.80 | 2.42 | 0.02568 | 0.99 | 2.047 | 0.2946 |


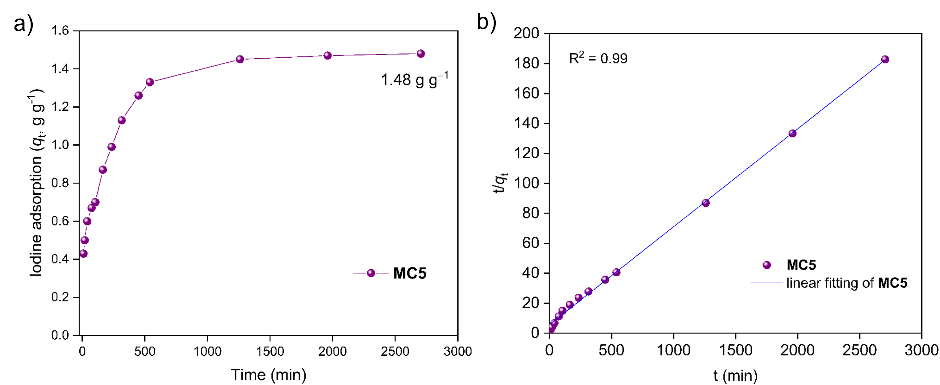


**Figure S53**. (a) Static adsorption of iodine vapor by **MC5** at 75 °C.
(b) Pseudo-second-order kinetic model describing the iodine adsorption behavior of **MC5**.


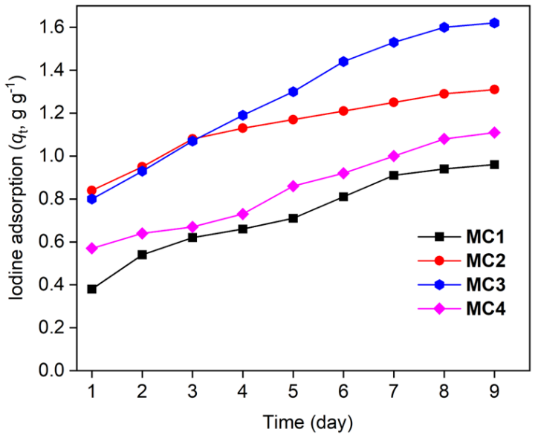


**Figure S54**. Iodine sorption onto the metal-organic cages (**MC**) at room temperature and ambient pressure.

**8**. **Absorption Mechanism Investigation**

**FT-IR Analysis**:


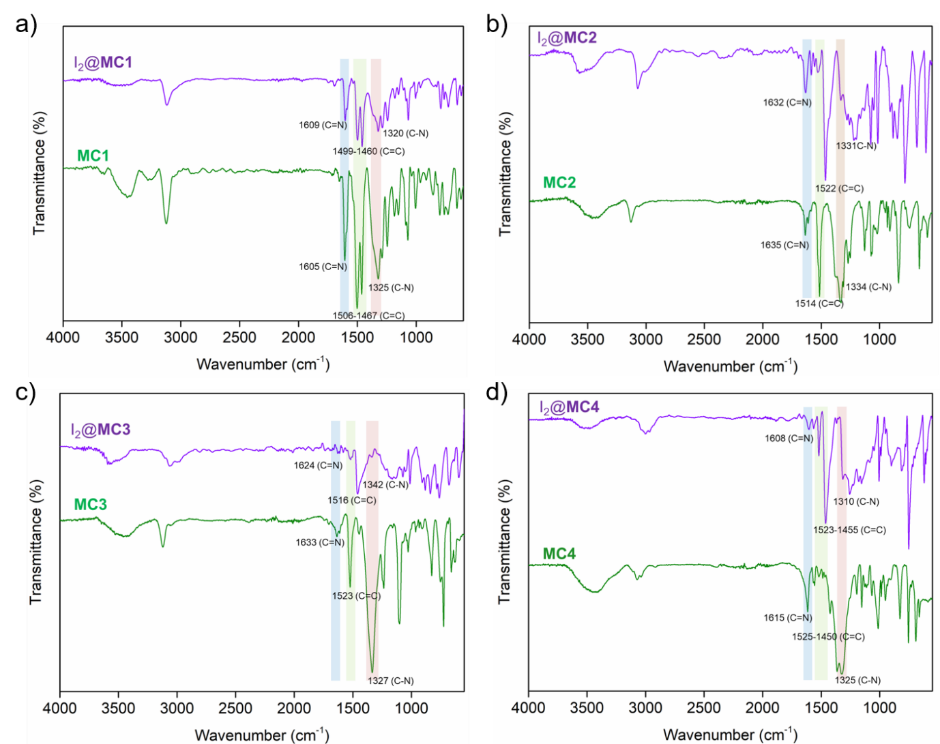


**Figure S55**. Comparative FT-IR spectra of (a) **MC1**, (b) **MC2**, (c) **MC3** and (d) **MC4** before (green) and after iodine adsorption (violet) showing potential interactions between iodine and the cage.

**TGA Analysis**:


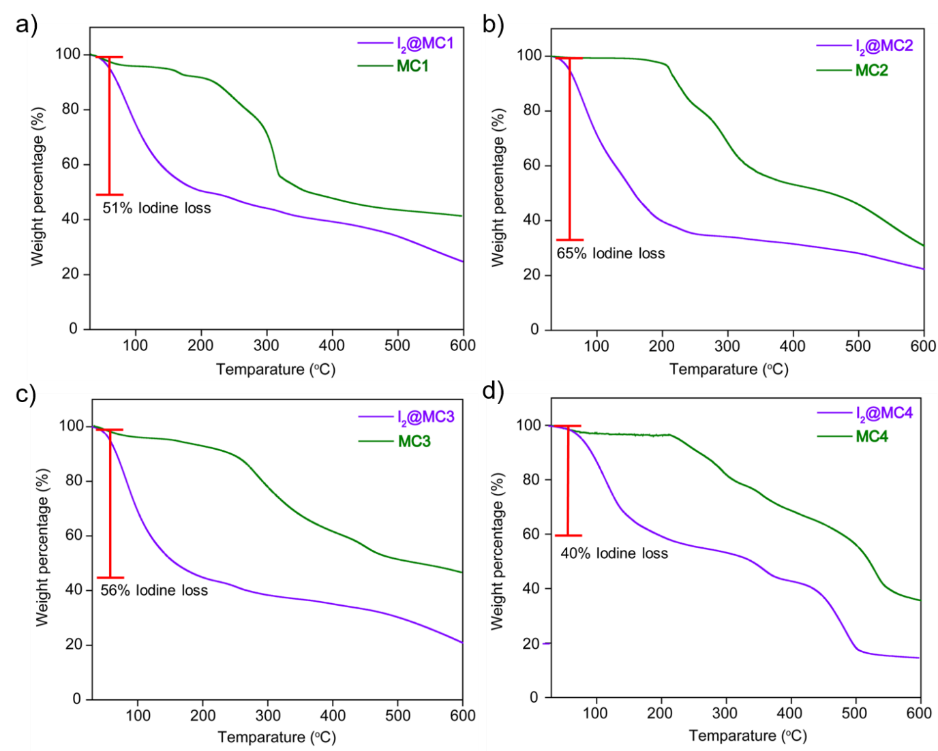


**Figure S56**. Thermogravimetric analysis of the pristine cages (green curve) and the iodine-loaded cages material (purple curve). A notable weight loss of approximately 51%, 65%, 56%, and 40% (wt%) was observed for I_2_@**MC1,** I_2_@**MC2,** I_2_@**MC3** and I_2_@**MC4**, respectively, between 40 °C and 180 °C, indicating the release of adsorbed iodine.

**PXRD Analysis**:


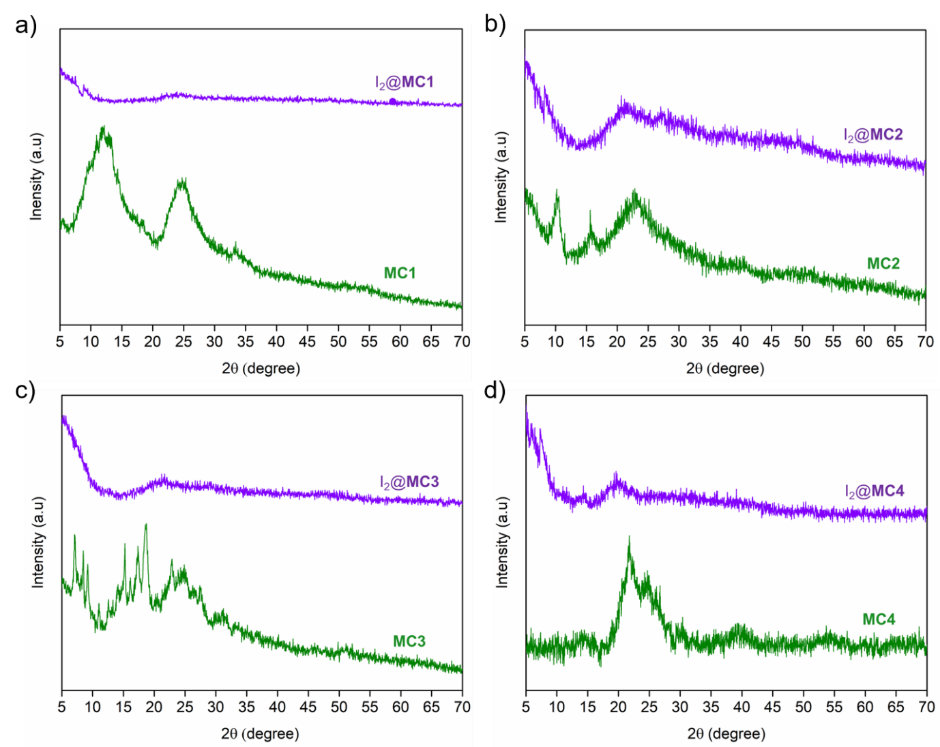


**Figure S57**. PXRD analysis of (a) **MC1**, (b) **MC2**, (c) **MC3**, and (d) **MC4** to examine structural changes after iodine adsorption. The green and purple curves represent the diffraction patterns of the pristine cages and iodine-loaded cages, respectively.

**EPR Spectroscopy**:


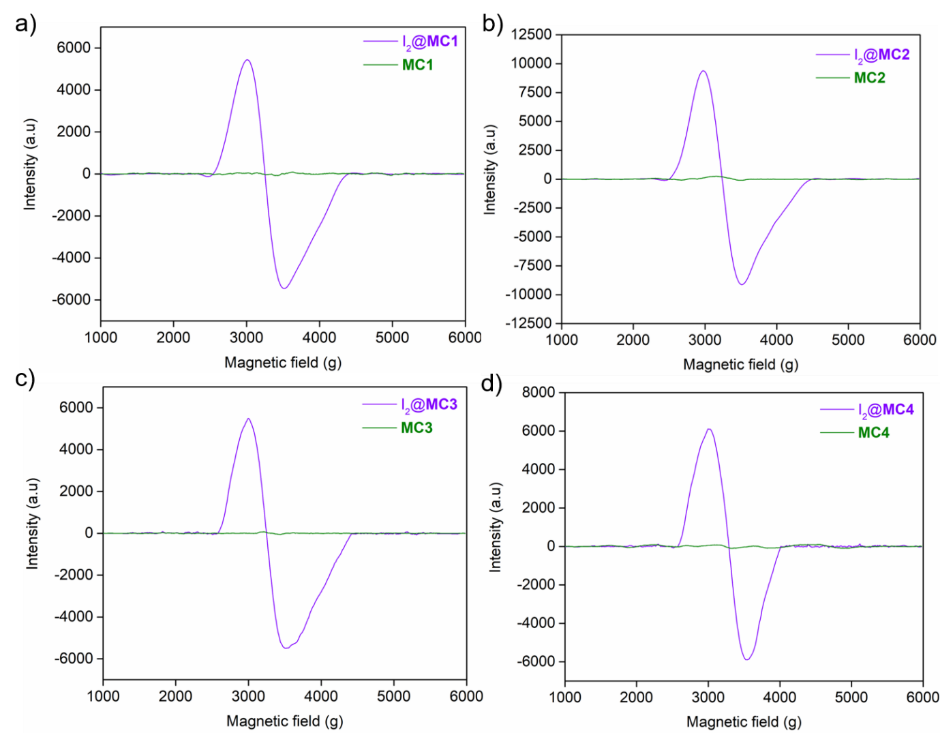


**Figure S58**. EPR spectroscopic analysis of cages **MC1**, **MC2**, **MC3**, and **MC4** before and after iodine adsorption.

**Raman Spectroscopy Analysis**:


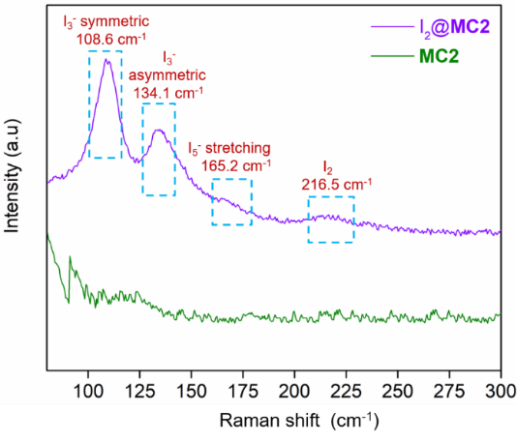


**Figure S59**. Raman spectra of cage **MC2** (green) and I_2_@**MC2** (violet).


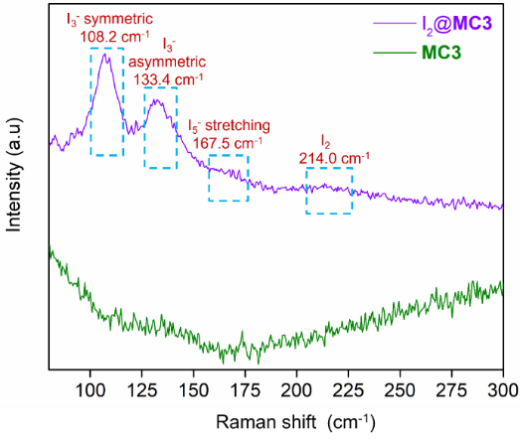


**Figure S60**. Raman spectra of the cage **MC3** before (green) and after (violet) iodine adsorption.

**
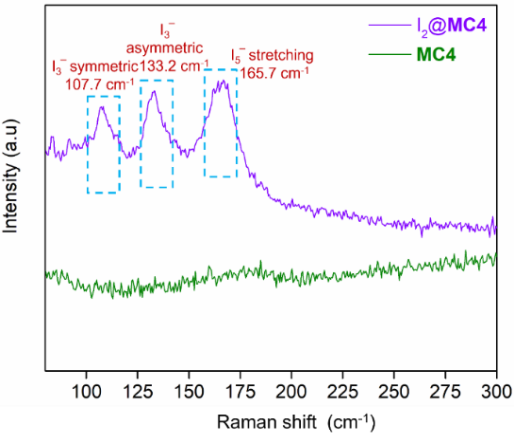
**

**Figure S61**. Raman spectra of **MC4** before (green) and after (violet) iodine adsorption.

**FE-SEM Image**:


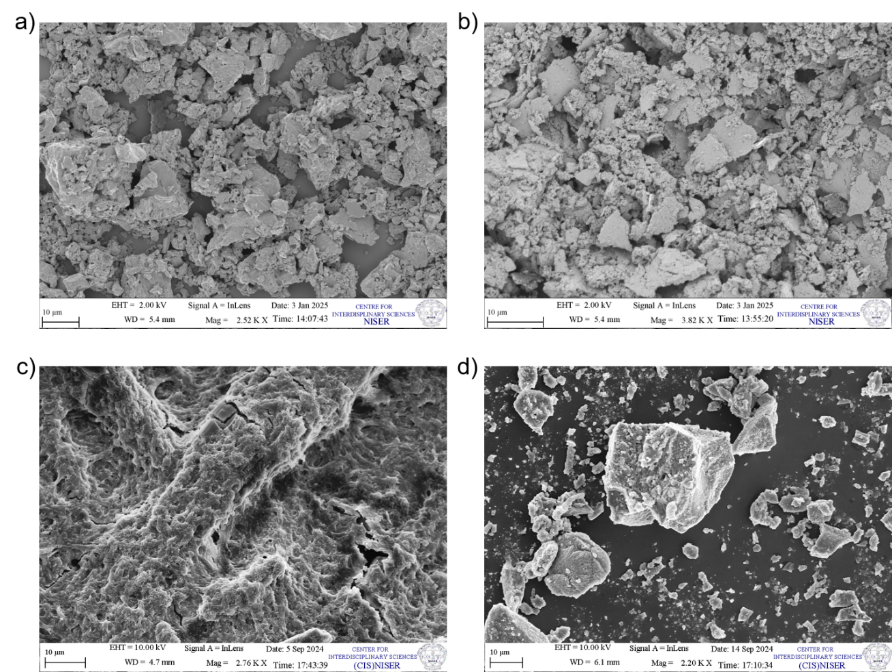


**Figure S62**. FE-SEM image of (a) I_2_@**MC1,** (b) I_2_@**MC2,** (c) I_2_@**MC3,** and (d) I_2_@**MC4**.

**EDAX Analysis and EDS Mapping**:


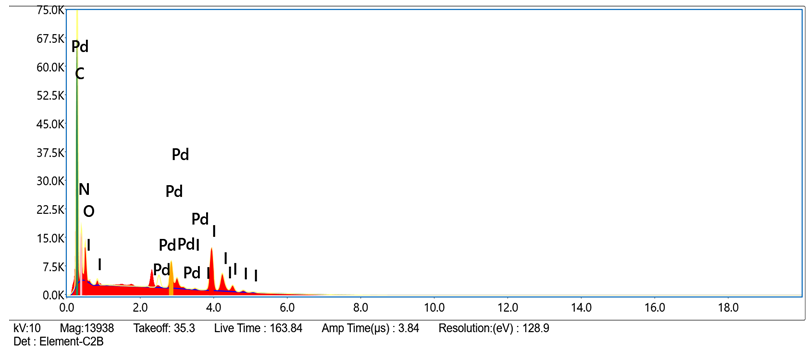


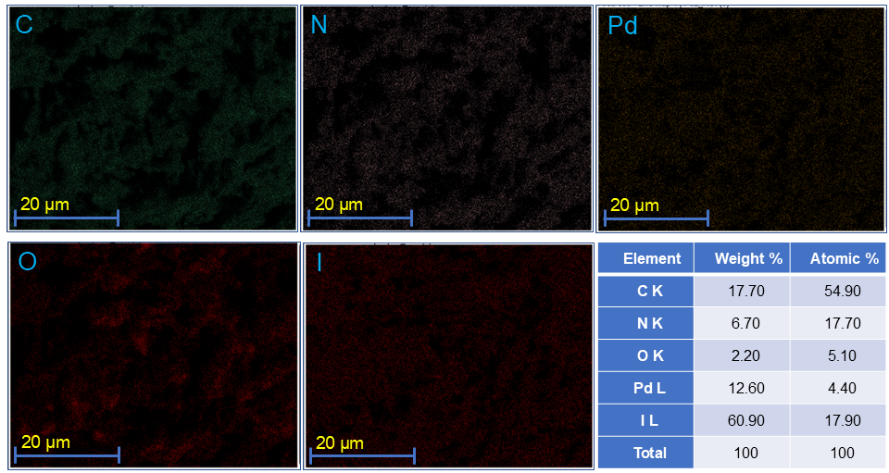


**Figure S63**. EDAX analysis of I_2_@**MC1** (top) and corresponding EDS elemental mapping (bottom).


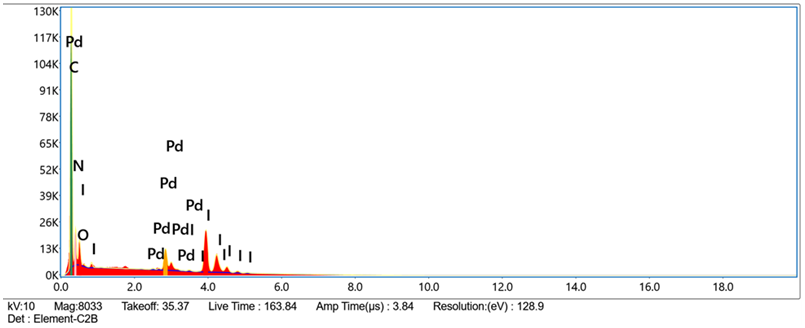


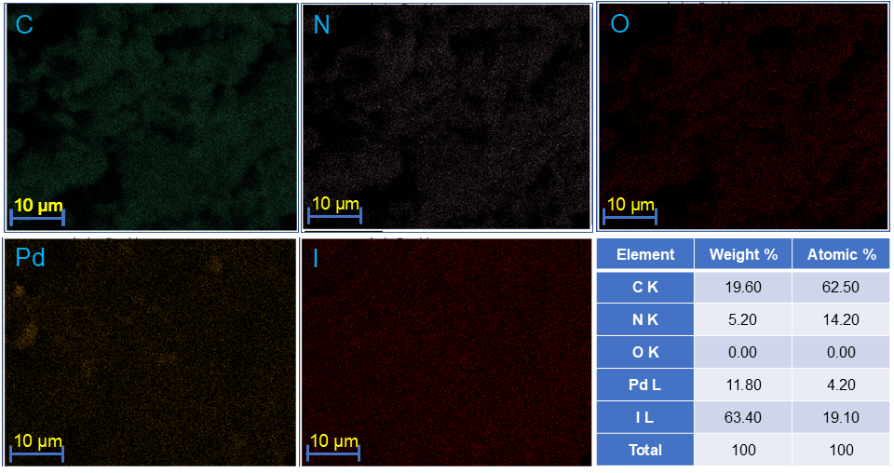


**Figure S64**. EDAX analysis of I_2_@**MC2**, along with elemental mapping of iodine, confirming successful iodine adsorption and its uniform distribution within the cage **MC2**.


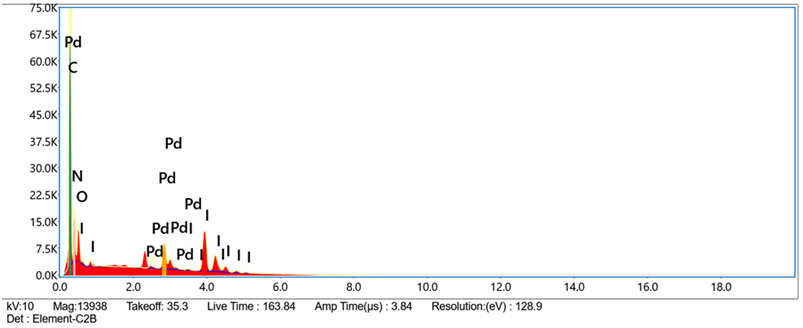


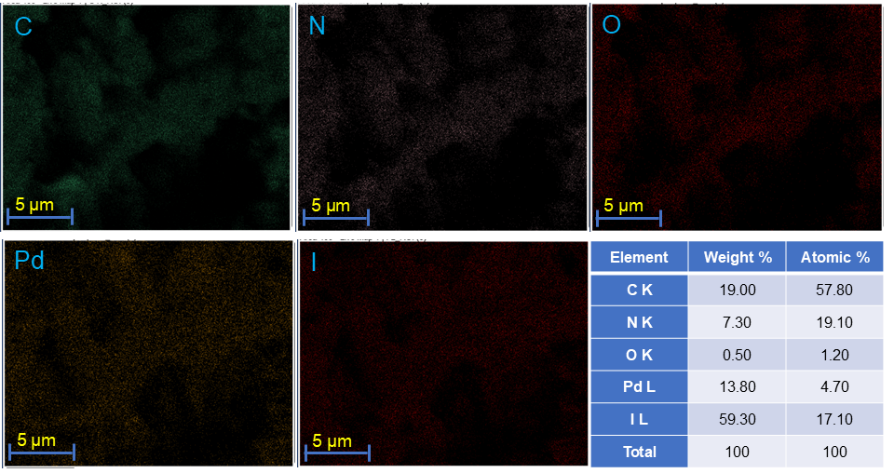


**Figure S65**. EDAX analysis of I_2_@**MC3** (top) and corresponding EDS elemental mapping (bottom).


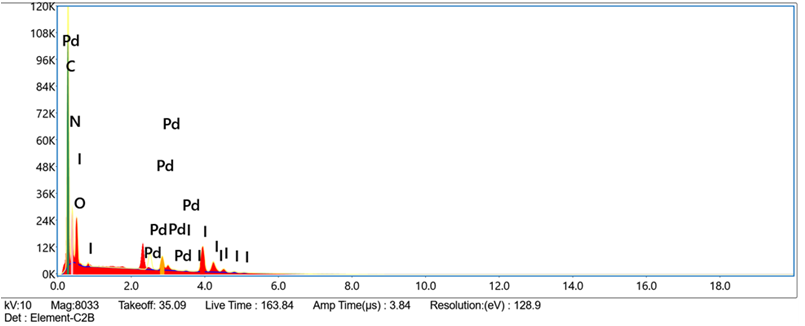


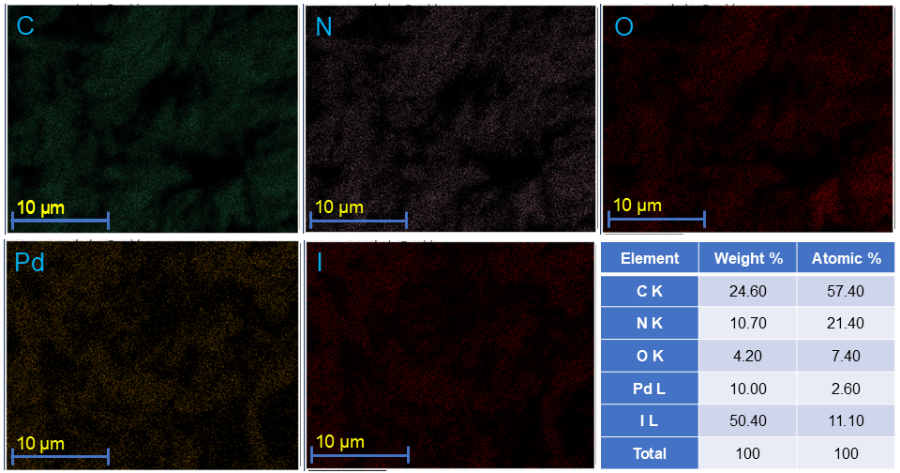


**Figure S66**. EDAX results for I_2_@**MC4** (top) and associated EDS elemental mapping (bottom), illustrating the spatial distribution of iodine within the cage framework.

**XPS Analysis**:


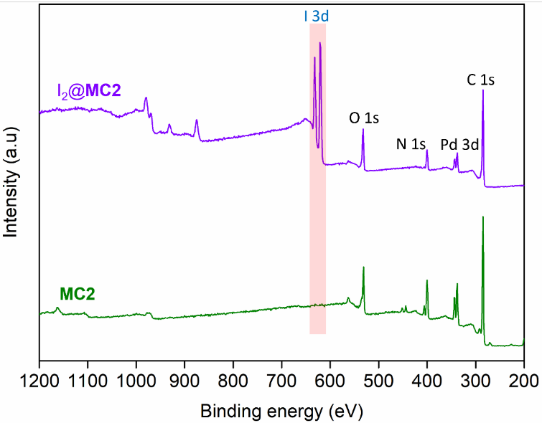


**Figure S67**. Full XPS spectra of **MC2** before (green) and after (violet) iodine adsorption.


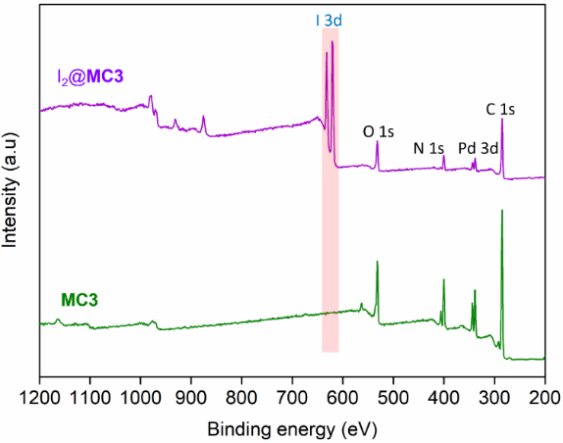


**Figure S68**. Comprehensive XPS survey scans of **MC3** cage material before (green) and after (violet) iodine adsorption.


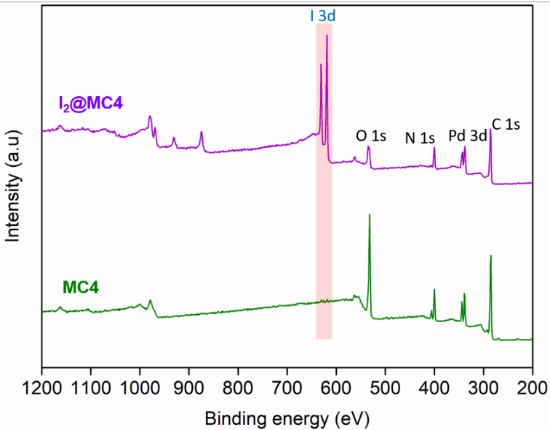


**Figure S69**. Comprehensive XPS spectra of **MC4** before (green) and after (violet) iodine adsorption.


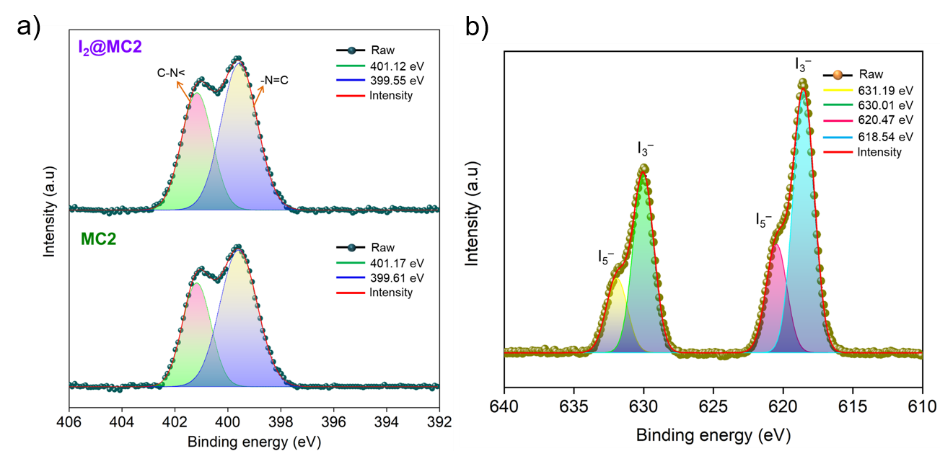


**Figure S70**. (a) Comparative XPS analysis of N 1s orbitals between I_2_@**MC2** and the empty cage. (b) XPS spectra of I 3d for **MC2** after iodine exposure.


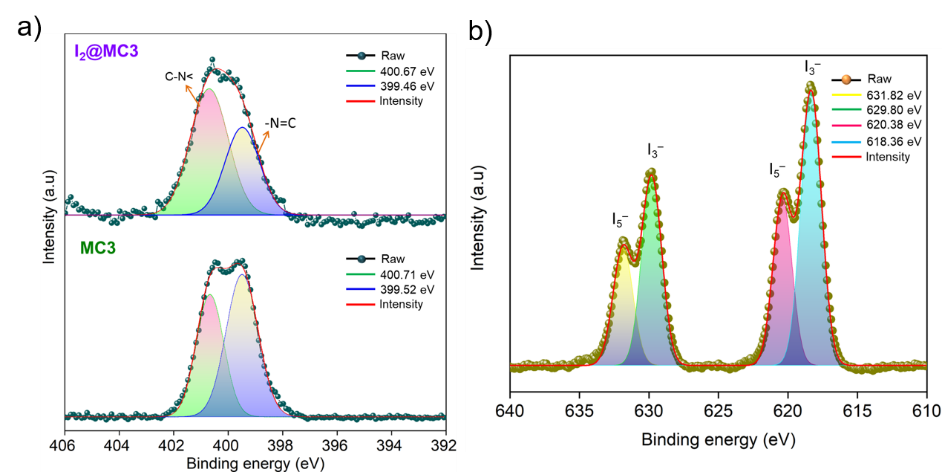


**Figure S71**. (a) XPS of the N 1s orbitals for **MC3** and I_2_@**MC3.** (b) XPS spectra of I 3d for I_2_@**MC3**.


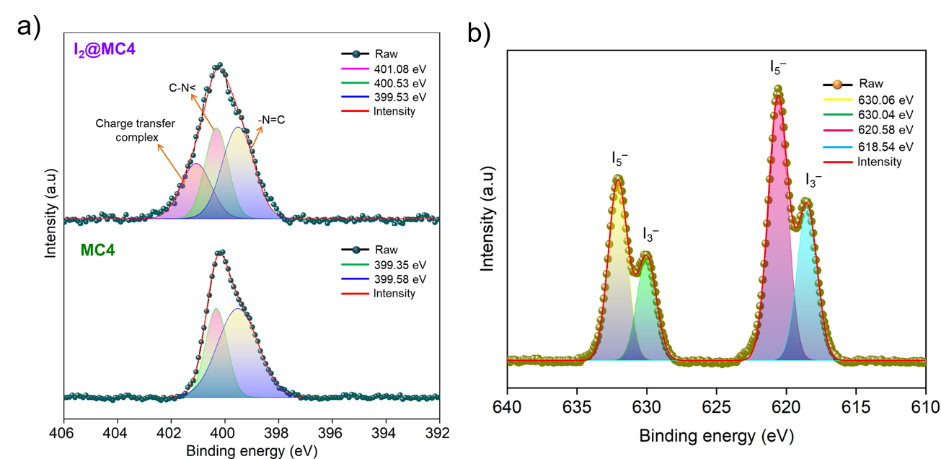


**Figure S72**. a) Comparative XPS analysis of N 1s binding energies in I_2_@**MC4** relative to the pristine **MC4** cage. (b) XPS characterization of the I 3d spectra in **MC4** following iodine exposure.


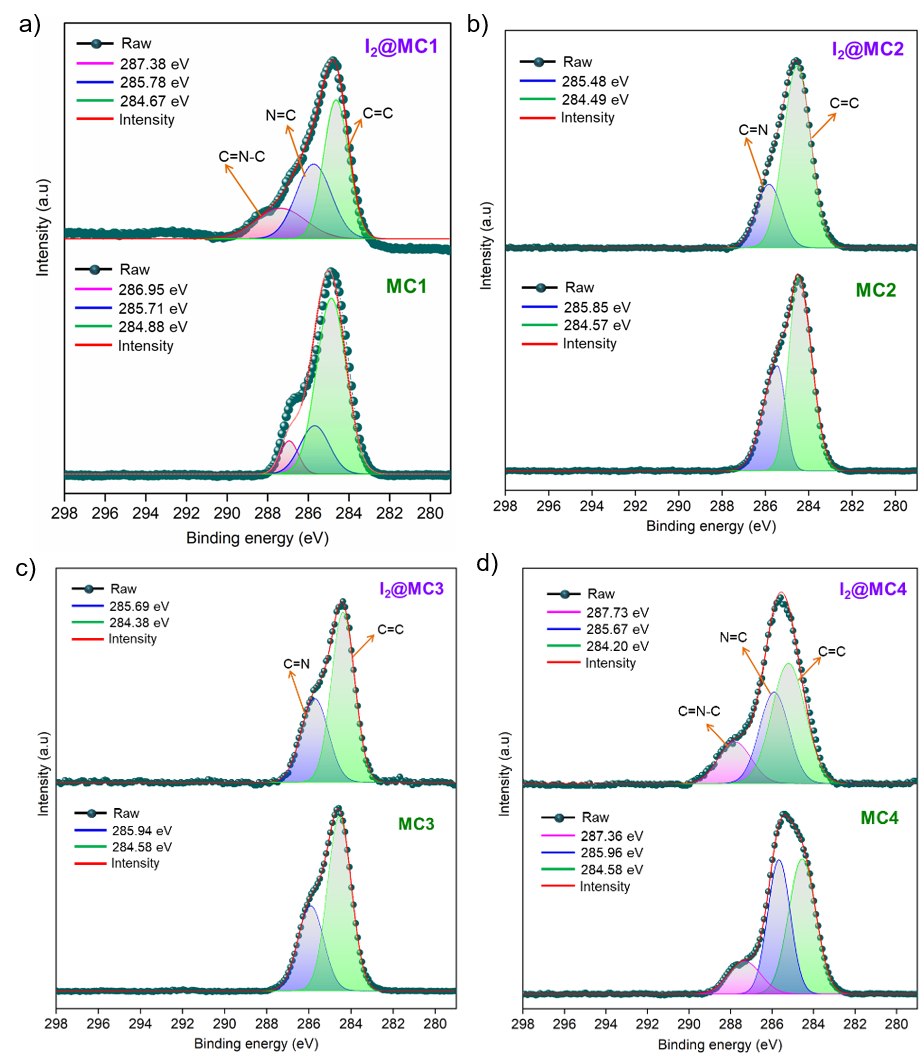


**Figure S73**. XPS spectra of C 1s for (a) **MC1** (b) **MC2** (c) **MC3** and (d) **MC4** before and after exposure to iodine vapor for 36 h.

**9**. **Iodine Uptake Investigation in Aqueous Phase**

**Adsorption from an Aqueous Iodine Solution**:

To evaluate the efficacy of the cages (**MC**) for iodine adsorption from aqueous solutions, 15 mg of each **MC** sample was evenly distributed across 5 vials, with each vial containing 3 mg of the material. Subsequently, 3 mL of a saturated aqueous iodine solution (1.2 mM) was added to each vial. The contents of the vials were stirred at ambient temperature approximately 25 °C for varying predetermined time intervals. Following this, the suspensions were centrifuge, and the residual iodine concentration in each filtrate was quantified using UV-Vis spectroscopy. A calibration curve was employed for quantitative analysis. The iodine removal efficiency of the cage was calculated using the following equation:

Removal efficiency = $\frac{C_{0}-C_{t}}{C_{0}} \times100$……………………. (Eq.3)

Where *C*_0_ (mM) and *C*_t_ (mM) represent the initial and final iodine concentrations, respectively, in the aqueous solution before and after treatment with the cage.

The quantity of iodine adsorbed from the aqueous phase was determined using the following equation:

Quantity of iodine uptake (*q*_t_) = $\frac{C_{0}-C_{t}}{m}\times v\times M$…………..…(Eq.4)

Where *q*_t_ (mg g^-1^) represents the amount of iodine adsorbed per gram of adsorbent at time t (min). *C*_0_ (mM) and *C*_t_ (mM) denote the initial and at a time iodine concentrations, respectively, in the aqueous solution before and after treatment with the cage adsorbent. *M* (g mol⁻^1^) is the molecular weight of iodine, *v* (mL) corresponds to the volume of iodine solution and *m* (mg) is the mass of the cage used in the adsorption experiment.

**Maximum Iodine Uptake from a Saturated Aqueous Iodine Solution**:

To accurately evaluate the iodine adsorption capacity of the cages, we performed a control experiment to account for iodine loss due to volatilization. This allowed us to distinguish between iodine physically adsorbed by the cages and that lost to evaporation.

**Control Experiment**: A 10 mL aliquot of aqueous iodine solution ($C_{0}$ = 1.2 mM) was stirred at ambient temperature for 48 h in a sealed vial without any adsorbent. Afterward, the residual iodine concentration (𝐶_c_) was measured via UV-Vis spectroscopy. The amount of iodine lost due to volatilization ${(Q}_{C}$) was calculated using:

$$Q_{C}=\left( C_{0}-C_{c} \right)\times v\times M$$

where *v* is the volume of the solution, and *M* (g mol⁻^1^) is the molecular weight of iodine.

**Adsorption Experiment**: Each cage sample (15 mg) was added to a round-bottom flask containing 200 mL of saturated iodine solution (1.2 mM) and stirred at room temperature for 48 h. After adsorption, the cage material was separated by filtration. The residual iodine concentration in the filtrate ($C_{f}$) was determined using UV-Vis spectroscopy and compared against a calibration curve. The apparent iodine uptake ($Q_{a}$) was calculated as:

$$Q_{a}=\left( C_{0}-C_{f} \right)\times v\times M$$

**Adsorption Capacity**: The actual adsorption capacity per gram of cage material ($Q_{t}$)^[11]^ was determined by correcting for iodine volatilization:

$$Q_{t}=\frac{\left( Q_{a}-Q_{c} \right)}{m}\times M$$

where *m* is the mass of the cage. This method ensures that the calculated adsorption capacity reflects only the iodine retained by the adsorbent, excluding losses due to evaporation.

**Experiments to Assess Selective Adsorption**:

3 mg of the each cage under investigation was introduced into an aqueous iodine solution (20 mL, 1.2 mM) containing 1-100 equiv. of all salt of KF, KCl, KBr, K_2_SO_4_ and KNO_3_. The suspension was subjected to magnetic stirring at 25 °C for 60 minutes. Subsequently, the cage was isolated via centrifuge, and the residual iodine concentration in the filtrate was determined using UV-Vis spectrophotometry. The removal efficiency of the cage was calculated using Eq.3. A control experiment was conducted using 20 mL of iodine aqueous solution instead of the competing anion solution for comparison.

Distribution coefficient (*k*_d_) was calculated using the following Eq.5.^[12]^

Distribution coefficient ($k_{d})=\left( \frac{c_{0}-c_{t}}{c_{t}} \right)\times\frac{v}{m}$………….… (Eq.5)

In this context, k_d_ represents the distribution coefficient, where *C*_0_ and *C*_t_ denote the initial and final iodine concentrations, respectively, in the aqueous solution. *v* (mL) signifies the volume of the solution, while *m* (mg) represents the mass of the sorbent.

**Preparation of a 0.5 mM aqueous solution of I₃⁻**:

Triiodide (I_3_⁻) solutions were prepared fresh immediately before experiments. This was accomplished by dissolving 21 mg of iodine (I_2_) and 42 mg of potassium iodide (KI) in 100 mL of deionized water under continuous stirring.^[13]^ The resultant aqueous solution contained 0.50 mM I_3_⁻, as governed by the equilibrium reaction: I_2_ (aq) + I⁻ (aq) ⇌ I_3_⁻ (aq), with an equilibrium constant K ≈ 783.^[14]^

**Aqueous Phase Static Tri-iodide (I_2_/KI) Capture Studies**:

For determining the maximum uptake capacity of the cages, 15 mg of each cage material was introduced into a vail containing 3 g potassium iodide (KI), 1.5 g iodine (I_2_), and 6 mL of deionized water. The resulting mixture was subjected to stirring at ambient temperature for a duration of 48 hours. Subsequently, the cage samples were isolated via filtration, washed thoroughly with deionized water, and allowed to air-dry, yielding a black solid product. The filtrate was collected and subjected to a bisulfite-starch titration, as described below.^[11, 15]^

**Bisulfite Starch Titration**:

5 mL of a 1% (w/v) aqueous starch indicator solution was added to the combined filtrate and washings solution. The resulting mixture was then titrated dropwise with a 0.05 M aqueous sodium bisulfite solution until the characteristic blue color of the starch-iodine complex was completely discharged. The iodine adsorption capacity of the **MC** under investigation was subsequently determined by quantifying the net change in iodine concentration within the aqueous solution before and after cage exposure.

**Selectivity Test in Presence of Other Competing Ions**:

To investigate the influence of competing anions on iodide capture, 3 mg cage were individually exposed to solutions consisting of 3 mL of I_3_⁻ (2.5 mM) solution and containing 1-100 equiv. of a single competing anion (NO_3_^-^, Cl^-^, Br^-^, F^-^, SO_4_^2-^) salt. These mixtures were stirred for 60 minutes at ambient temperature. Subsequently, the cage samples were isolated via centrifuge, and the residual I_3_⁻ concentration in the filtrate was determined by UV-Vis spectrophotometric analysis. The capture efficiency in the presence of each competing anion was evaluated relative to a control experiment where 3 mL of I_3_⁻ (2.5 mM) solution was used without the competing anion solution. The removal efficiency and distribution coefficient were calculated from the following Eq.4 and Eq.5 respectively.

**Isotherm Models of Iodine Adsorption**:

To elucidate the adsorption mechanism, the Langmuir and Freundlich isotherm models were employed to simulate the experimental adsorption data.^[10]^ The Langmuir model, which describes monolayer adsorption, signifies that each adsorption site can accommodate only a single adsorbate molecule and that there are no significant interactions between adsorbed molecules on neighbouring sites. This model is mathematically represented by Eq.6.

$\frac{Cⅇ}{q_{e}}=\frac{1}{k_{L}q_{m}}+\frac{Cⅇ}{q_{m}}$ ………………………………..(Eq.6)

where *k*_L_ (L mg⁻^1^) is the Langmuir constant, and *q*_m_ (mg g⁻^1^) is the maximum adsorption capacity.

The Freundlich model represents as Eq.7.

$\ln q_{e}=\ln k_{F}+\left( \frac{1}{n} \right)\ln c_{e}$…………………….. (Eq..7)

Where, *k*_F_ and (1/n) are the Freundlich model constant, indicating capacity and intensity of adsorption respectively.


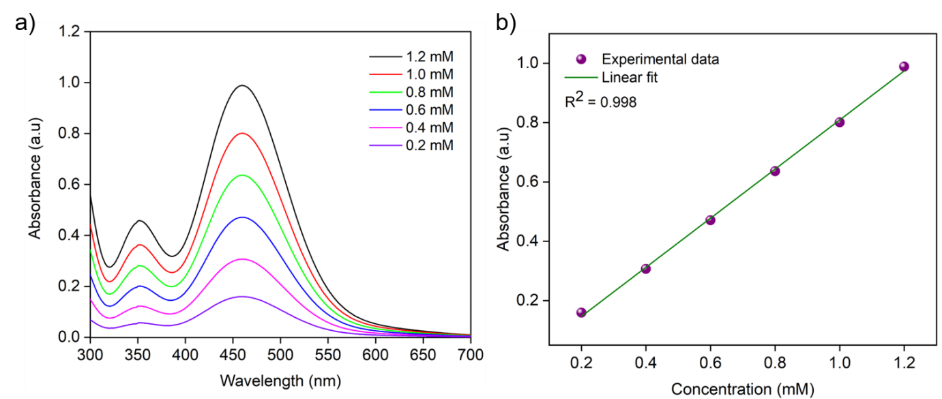


**Figure S74**. (a) UV-Vis absorption spectra of aqueous iodine solutions at varying concentrations. (b) A standard curve was constructed based on the absorbance values measured at 460 nm.


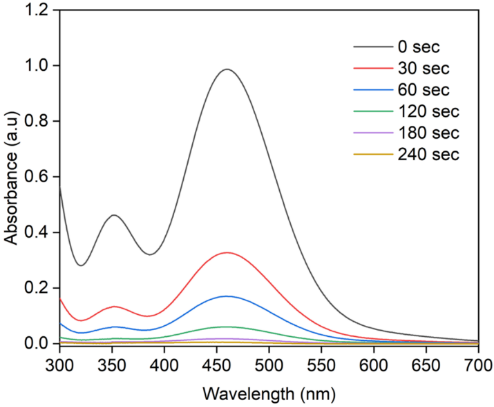


**Figure S75**. Time-resolved UV-Vis spectra of molecular iodine (I_2_) in aqueous solution demonstrating rapid diminishing upon treatment with **MC1**.


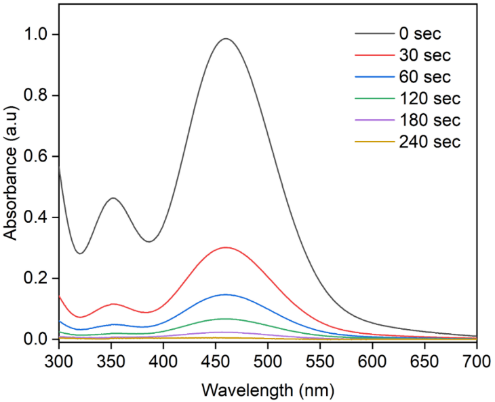


**Figure S76**. Time-dependent UV-Vis spectra of 3 mL iodine in aqueous (1.2 mM) upon the addition of 3 mg of **MC3**.


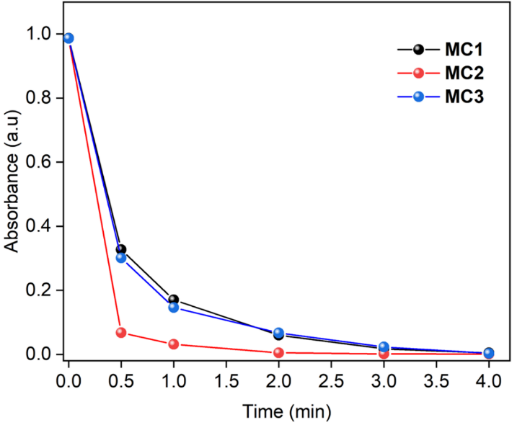


**Figure S77**. Profile of decrease in the absorbance of iodine in water with time by the treatment of **MC1**, **MC2,** and **MC3**.


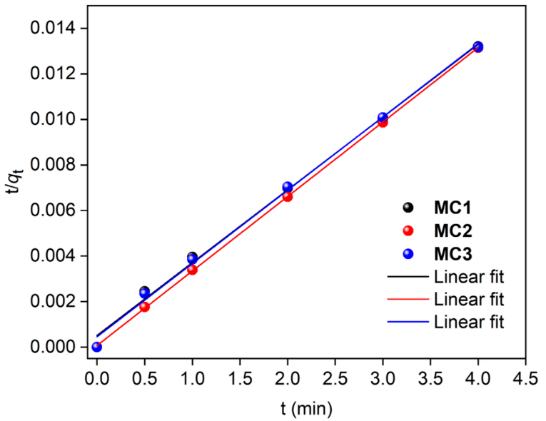


**Figure S78**. Pseudo-second-order linear fit for iodine adsorption from water by treating with different cages.

**Table S5**. Pseudo second order kinetic parameter for the adsorption of iodine from aqueous solution.

| Cage | *q*_e_ (mg g⁻^1^)  (calculated) | *k*_2_  (g mg⁻^1^ min⁻^1^) | R^2^ |
| --- | --- | --- | --- |
| **MC1** | 312.5 | 0.02198 | 0.99 |
| **MC2** | 305.81 | 0.1463 | 0.99 |
| **MC3** | 310.55 | 0.022621 | 0.99 |


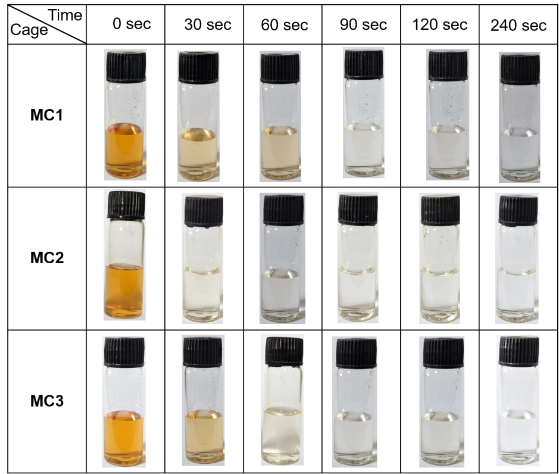


**Figure S79**. Digital images of color changes of the iodine (I_2_) solution in water with time upon treatment of **MC1**, **MC2** and **MC3**.


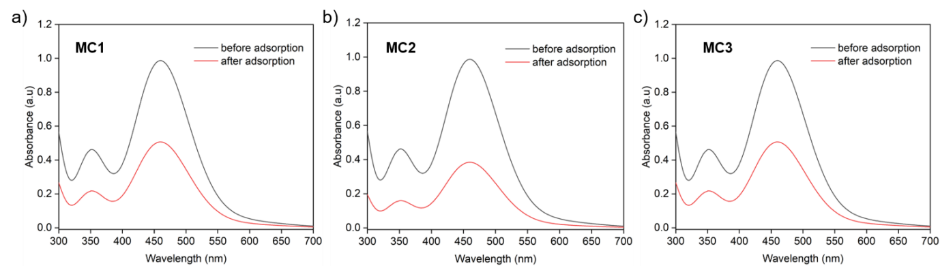


**Figure S80**. UV-Vis spectra of 200 mL of a 1.2 mM aqueous iodine aqueous solution were recorded before and after the introduction of 15 mg of (a) **MC1**, (b) **MC2**, and (c) **MC3**, respectively.


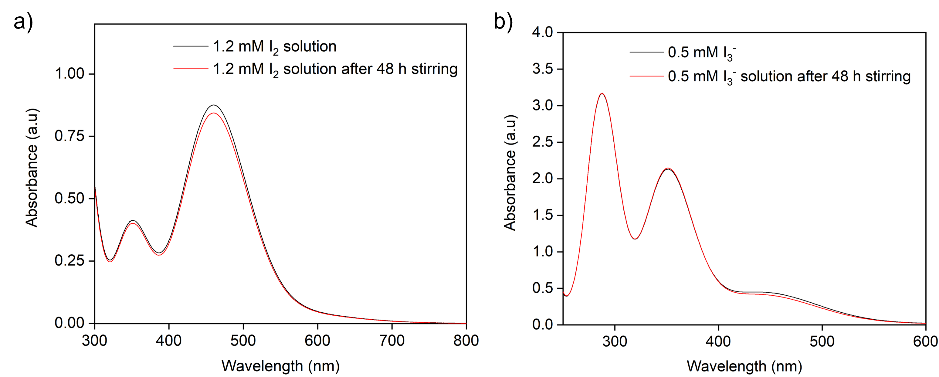
**Figure S81**. UV-Vis spectra of (a) an aqueous solution of molecular iodine (I₂), and (b) an aqueous solution of an I₂/KI mixture before and after 48 h of stirring.

**
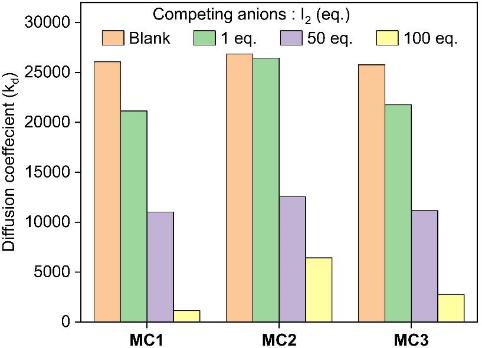
**

**Figure S82**. Determination of relative distribution coefficients (*k*_d_) for I_2_ in aqueous solutions containing varying concentrations (1-100 equivalents) of different interfering anions together in the presence of the cage materials.


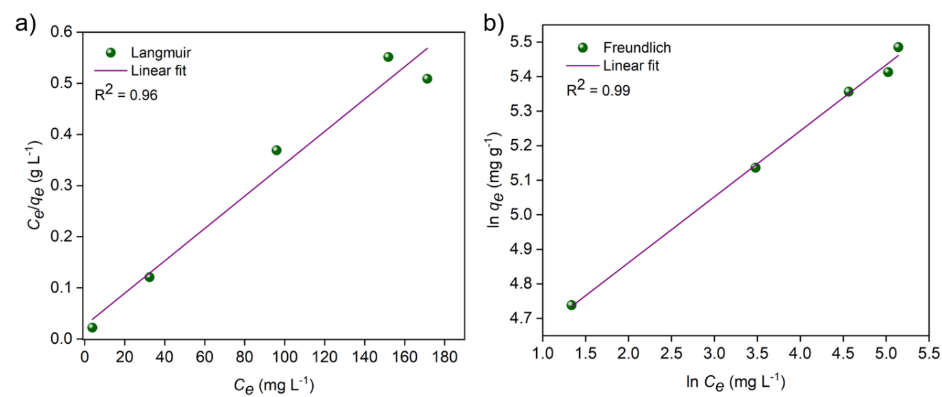


**Figure S83**. (a) Langmuir isotherm model of iodine adsorption in an aqueous solution by cage **MC1**. (b) Freundlich isotherm model of iodine adsorption in an aqueous solution by cage **MC1**.


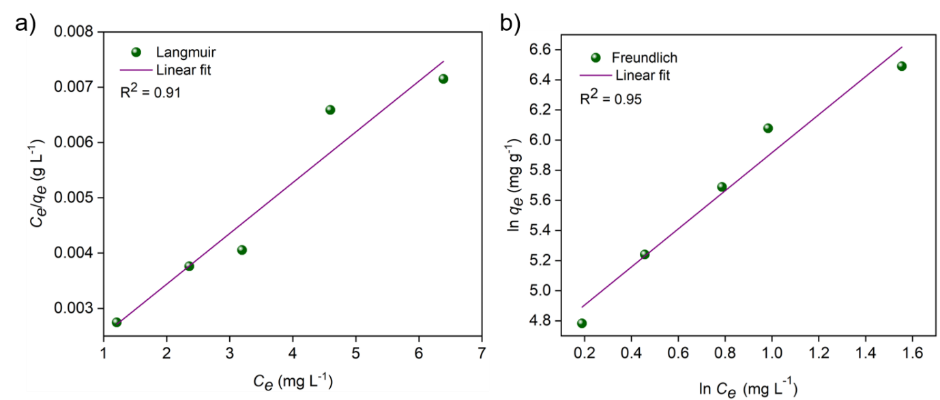


**Figure S84**. Iodine adsorption isotherms of cage **MC2** in aqueous solution.

**
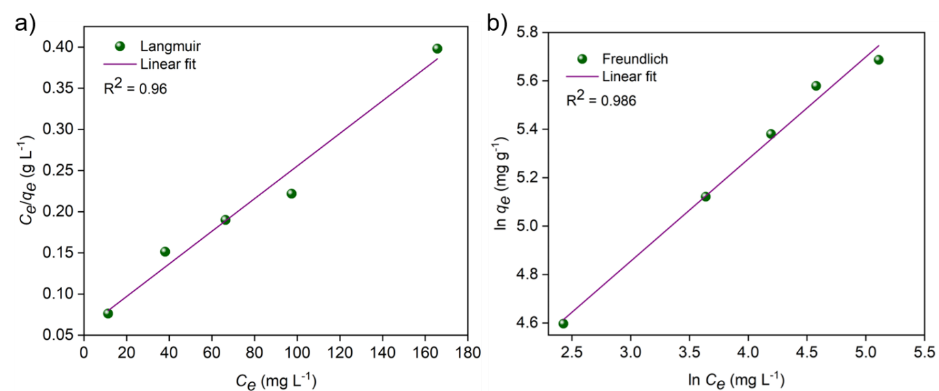
**

**Figure S85**. (a) Langmuir and (b) Freundlich isotherm models for the adsorption of iodine in an aqueous solution by cage **MC3**.

**Table S6**. Isotherm parameters of two model

| Cage | Langmuir isotherm model | | | Freundlich isotherm model | | |
| --- | --- | --- | --- | --- | --- | --- |
|  | *q*_m_  (mg g^-1^) | *k*_L_  (L mg^-1^) | R^2^ | 1/*n* | *k*_F_  (L mg^-1^) | R^2^ |
| **MC1** | 505.05 | 0.0345 | 0.96 | 5.2391 | 88.19 | 0.99 |
| **MC2** | 468.44 | 0.16125 | 0.91 | 1.1682 | 89.51 | 0.95 |
| **MC3** | 316.45 | 0.082874 | 0.96 | 2.3614 | 36.51 | 0.98 |


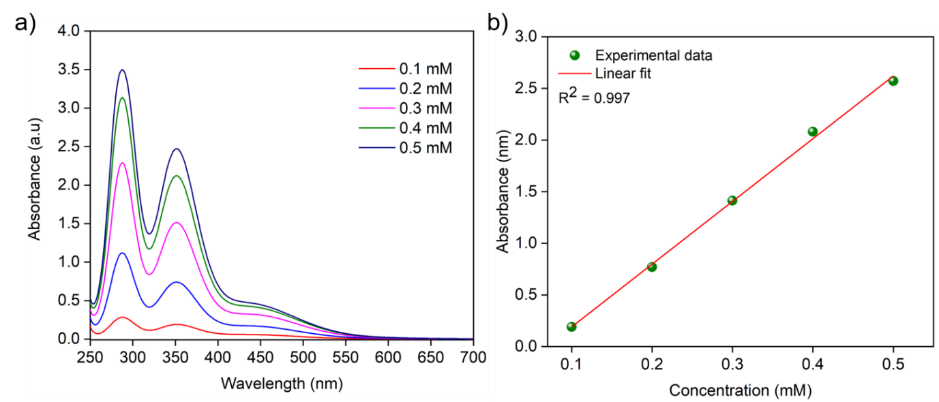


**Figure S86**. (a) UV-Vis absorption spectra of aqueous I_3_^−^ solutions were acquired over a range of concentrations. (b) Calibration curve was constructed by plotting the corresponding absorbance.


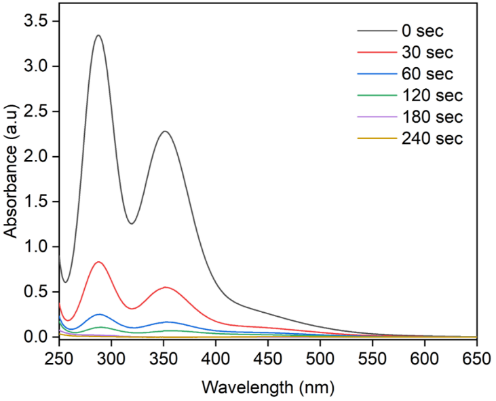


**Figure S87**. Time-dependent UV-Vis spectra of I_3_^−^ solution in presence of **MC1**.


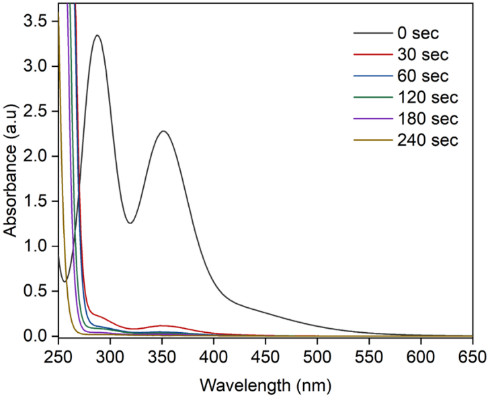


**Figure S88**. UV-Vis spectra of I_3_^−^ solutions in the presence of **MC2** over time.


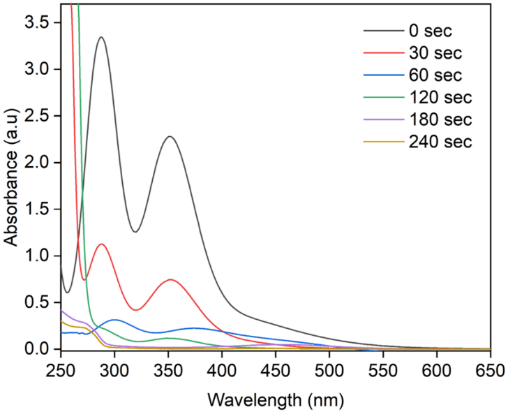


**Figure S89**. UV-Vis spectra of I_3_⁻ solutions in the presence of **MC3** over time.


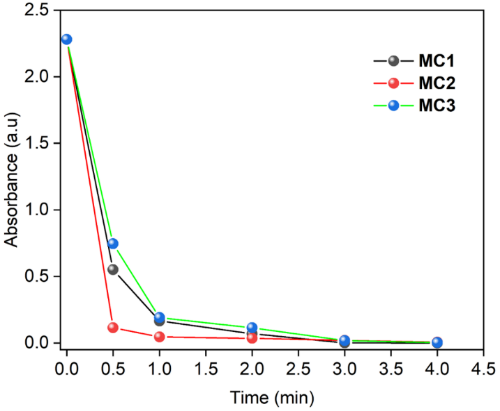


**Figure S90**. The profile of the reduction in I_3_^−^ absorbance in water over time following treatment with **MC1**, **MC2**, and **MC3**.


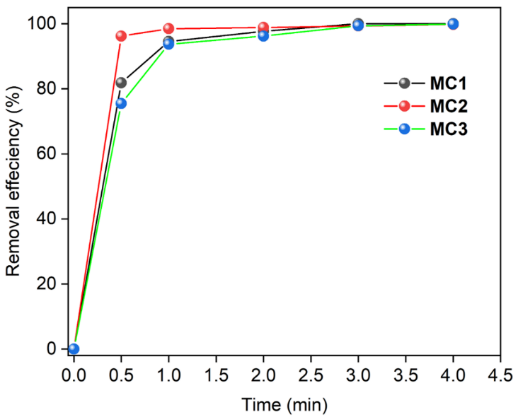


**Figure S91**. Rapid increase in removal efficiency of I_3_^−^ from aqueous solution upon the addition of cage.

**Figure S92**. Kinetic analysis of I_3_^−^ adsorption from aqueous using the pseudo-second-order model.

**Table S7**. Kinetic parameters for I_3_^−^ adsorption by the cage

| Cage | *q*_e_ (mg g⁻^1^)  (calculated) | *k*_2_  (g mg⁻^1^ min⁻^1^) | R^2^ |
| --- | --- | --- | --- |
| **MC1** | 193.05 | 0.08391 | 0.99 |
| **MC2** | 190.11 | 0.4251 | 0.99 |
| **MC3** | 193.74 | 0.0597 | 0.99 |

**Figure S93**. (a) Adsorption of I_3_^−^ from aqueous solutions containing varying excess amounts (1 to 100 equivalents) of individual competing anions (Br^−^, Cl^−^, F^−^, NO_3_^−^, and SO_4_^2−^) using **MC1**. (b) Corresponding relative distribution coefficients (*k*_d_) for I₃⁻ in the presence of each competing anion.

**Figure S94**. (a) I_3_^−^ removal from aqueous solutions containing an excess (1 to 100 equivalents) of individual competing anions (Br^−^, Cl^−^, F^−^, NO_3_^−^, and SO_4_^2−^) in the presence of **MC2**. (b) Corresponding relative distribution coefficients (*k*_d_) for I_3_^−^ in the presence of each competing anion.

**Figure S95**. (a) I_3_^−^ adsorption from aqueous solutions containing varying excess amounts (1 to 100 equivalents) of a single competing anion, viz. Br^−^, Cl^−^, F^−^, NO_3_^−^, or SO_4_^2−^, in the presence of **MC3**. (b) Corresponding relative distribution coefficients (*k*_d_) for I_3_^−^ in the presence of each of the competing anions.

**Figure S96**. Adsorption isotherms of (**a**) Langmuir and (**b)** Freundlich models for I_3_^−^ adsorption by **MC1**.

**Figure S97**.  Data fitting of (a) Langmuir and (b) Freundlich adsorption isotherms for I_3_^−^ adsorption in the presence of **MC2**.

**Figure S98**. Iodine adsorption isotherms of **MC3** in aqueous solution.

**Table S8**. Isotherm parameters for the adsorption of I_3_^−^ by the cages

| Cage | Langmuir isotherm model | | | Freundlich isotherm model | | |
| --- | --- | --- | --- | --- | --- | --- |
|  | *q*_m_  (mg g^−1^) | *k*_L_  (L mg^−1^) | R^2^ | 1/*n* | *k*_F_  (L mg^−1^) | R^2^ |
| **MC1** | 196.68 | 0.0527 | 0.81 | 1.269 | 47.14 | 0.91 |
| **MC2** | 197.40 | 0.6459 | 0.88 | 1.267 | 75.09 | 0.96 |
| **MC3** | 195.74 | 0.06732 | 0.89 | 1.1719 | 14.69 | 0.96 |

**Figure S99**. Time-dependent UV-Vis spectroscopic studies of I_2_/KI aqueous solutions: (a) 0.5 mM I_2_ + 2.5 mM KI, (b) 0.5 mM I_2_ + 5.0 mM KI, (c) 0.5 mM I_2_ + 10.0 mM KI in the presence of **MC1**. (d) Kinetic analysis of the I_2_/KI system using the pseudo-second-order model.

**Figure S100**. Time-dependent UV-Vis spectroscopy of I_2_/KI aqueous solutions: (a) 0.5 mM I_2_ + 2.5 mM KI, (b) 0.5 mM I_2_ + 5.0 mM KI, and (c) 0.5 mM I_2_ + 10.0 mM KI in the presence of **MC2**. (d) Kinetic analysis of the I_2_/KI system using the pseudo-second-order model.

**Figure S101**. Time-dependent UV-Vis spectroscopic studies of I_2_/KI aqueous solutions: (a) 0.5 mM I_2_ + 2.5 mM KI, (b) 0.5 mM I_2_ + 5.0 mM KI, (c) 0.5 mM I_2_ + 10.0 mM KI in the presence of **MC1**. (d) Kinetic analysis of the I_2_/KI system using the pseudo-second-order model.

**Table S9**. Comparison of the kinetics of different cages at varying amounts of KI

| Cage | Pseudo second order rate constant (*k*_2_)  (g mg⁻^1^ min⁻^1^) | | |
| --- | --- | --- | --- |
|  | 0.5 mM I_2_  + 2.5 mM KI | 0.5 mM I_2_  + 5.0 mM KI | 0.5 mM I_2_  + 10.0 mM KI |
| **MC1** | 0.2728 | 0.3779 | 0.5685 |
| **MC2** | 0.450 | 0.65275 | 0.7418 |
| **MC3** | 0.2246 | 0.34399 | 0.36776 |

**10**. **Dynamic I_3_^−^ Uptake in Aqueous Solution**

To evaluate the continuous I_3_^−^ capture efficiency of the cage material, a dynamic column-based flow-through experiment was performed. A dried 15 mg sample of the cage material was packed into a column and secured between two layers of degreased glass wool. A 0.5 mM I_3_^−^ solution was then continuously passed through the column using a water flow regulator. The effluent, collected in a 1 mL microcuvette after dynamic adsorption, was analyzed using UV-Vis spectrophotometry. The breakthrough volume was determined based on the breakthrough point observed during the column flow-through experiment.^[16]^

**Figure S102**. (a) UV-Vis spectra of the effluent collected after the breakthrough of **MC1**. (b) Corresponding breakthrough profile for I₃⁻ adsorption.

**Figure S103**. (a) UV-Vis spectra of the effluent collected after the breakthrough of **MC3** during I₃⁻ adsorption. (b) Dynamic flow-through adsorption of I₃⁻ on 0.5 mm **MC3** produced a breakthrough profile.

**Figure S104**. Photograph of the experimental setup for a column-based flow-through I_2_/KI capture study using the **MC2** cage as the adsorbent material.

**Figure S105**. Photographs of **MC2** packed columns after breakthrough experiments conducted with aqueous solution of I_3_^−^.

**11**. **Low Concentration Iodine Uptake from Aqueous Solution**

The Leuco-crystal violet method was effectively utilized for the determination of low-concentration iodine and iodide residues in seawater and drinking water.^[17]^ The detailed mechanism for solution preparation and subsequent measurement is outlined below.

**Scheme S7**. Mechanism of the Leuco-crystal violet method for determining I_2_/I⁻ residues.

**Preparation of Standard Iodide Solution**: A stock solution of 100 ppm iodide (I⁻) was prepared by dissolving 13.01 mg of potassium iodide (dried at 105 °C for 5 hours) in 100 mL of deionized water. Subsequent dilutions were performed to obtain a series of working standards ranging from 20 ppb to 100 ppb for the calibration curve.

**Preparation of Phosphate Buffer Solution**: A pH 4.0 phosphate buffer solution (100 mL) was prepared by dissolving 3.40 g of potassium dihydrogen phosphate (KH₂PO₄) in approximately 30 mL of distilled water in a 100 mL volumetric flask. To adjust the pH, 2 mL of 85% phosphoric acid was added, followed by dilution to a final volume of 100 mL with distilled water.

**Preparation of N-Chlorosuccinimide-Succinimide Solution**: A 100 mL solution was prepared by dissolving 100 mg (0.75 mmol) of N-chlorosuccinimide and 1.0 g (10.1 mmol) of succinimide in distilled water within a 100 mL volumetric flask.

**Preparation of Leuco-Crystal Violet Solution**: A 100 mL solution of leuco-crystal violet was prepared by adding 0.25 mL of 85% phosphoric acid and 25 mg (0.067 mmol) of 4,4',4"-methylidynetris (N, N-dimethylaniline) (leuco-crystal violet) to approximately 30 mL of distilled water in a 100 mL volumetric flask. The mixture was shaken vigorously until complete dissolution of the solid, and then diluted to a final volume of 100 mL with distilled water.

**Residual Concentration of [I_2_ + I⁻] Measurement**: To determine the residual concentration of [I₂ + I⁻], 25 mL of iodinated water was mixed with 1 mL of phosphate buffer solution and 1 mL of N-chlorosuccinimide-succinimide reagent in a suitable container. Subsequently, 1 mL of leuco-crystal violet solution was added, and the mixture was thoroughly shaken. The absorbance of the solution was measured at 591 nm using a UV-Vis spectrophotometer at 25 °C exactly 20 min after the addition of leuco-crystal violet. The residual concentration of [I₂ + I⁻] was determined using the calibration curve.

**Figure S106**. (a) UV-Vis spectra of standard iodide solution after 25 minutes of reaction with Leuco-crystal violet at different concentrations. (b) Standard curve based on absorbance at 591 nm.

**12**. **Iodine Adsorption from *n*-Hexane Solution**

Time-resolved UV-Vis absorption spectra were recorded for a solution of iodine in *n*-hexane (1.5 mM) following the addition of 5 mg of each cage materials (**MC1**, **MC2**, **MC3**, and **MC4**).

**Figure S107**. (a) UV-Vis spectra of iodine in *n*-hexane at different concentrations. (b) Calibration curve of iodine in *n*-hexane.

**Figure S108**. (a) Time-dependent UV-Vis absorption spectra of iodine in *n*-hexane after the addition of **MC1**. (b) Iodine adsorption from *n*-hexane solution.

**Figure S109**. (a) UV-Vis spectra showing iodine adsorption by cage **MC2** over time. (b) Corresponding I₂ removal efficiency in *n*-hexane solution.

**Figure S110**. (a) Time-dependent UV-Vis absorption spectra of iodine in *n*-hexane after the addition of **MC3**. (b) Iodine adsorption from *n*-hexane solution.

**Figure S111**. (a) UV-Vis spectra of iodine adsorption by cage **MC4** at different time intervals. (b) Corresponding removal efficiency of I_2_ in *n*-hexane solution.

**Figure S112**. Time-dependent absorbance changes at 523 nm in *n*-hexane.

**Figure S113**. Pseudo-second-order kinetic model for iodine adsorption by the cages (**MC1**, **MC2**, **MC3**, and **MC4**) from *n*-hexane solution.

**Table S10**. Kinetics parameter for the iodine adsorption from *n*-hexane solution

| Cage | *q*_e_ (mg g^−1^)  (calculated) | *k*_2_  (g mg^−1^ min^−1^) | R^2^ |
| --- | --- | --- | --- |
| **MC1** | 341.29 | 4.21 × 10^-5^ | 0.99 |
| **MC2** | 338.98 | 6.51 × 10^-5^ | 0.99 |
| **MC3** | 192.67 | 6.43 × 10^-5^ | 0.99 |
| **MC4** | 279.32 | 8.15 × 10^-5^ | 0.99 |

**Figure S114**. Photographs showing color changes in iodine *n*-hexane solutions after treatment with **MC1**, **MC2**, **MC3** and **MC4**.

**Figure S115**. Langmuir isotherm model for iodine adsorption in *n*-hexane solution by cages (a) **MC1**, (b) **MC2**, (c) **MC3**, and (d) **MC4**.

**Figure S116**. Freundlich adsorption isotherms of cages (a) **MC1,** (b) **MC2,** (c) **MC3 and** (d) **MC4** in *n*-hexane solution.

**Table S11**. Isotherm parameters for the adsorption of I_2_ from *n*-hexane solution

| Cage | Langmuir isotherm model | | | Freundlich isotherm model | | |
| --- | --- | --- | --- | --- | --- | --- |
|  | *q*_m_  (mg g^−1^) | *k*_L_  (L mg^−1^) | R^2^ | 1/*n* | *k*_F_  (L mg^−1^) | R^2^ |
| **MC1** | 363.64 | 5.54 × 10^−3^ | 0.94 | 1.619 | 6.52 | 0.986 |
| **MC2** | 392.22 | 3.28 × 10^−3^ | 0.90 | 4.296 | 6.30 | 0.989 |
| **MC3** | 205.65 | 8.19 × 10^−3^ | 0.95 | 0.828 | 12.51 | 0.99 |
| **MC4** | 385.43 | 4.52 × 10^−3^ | 0.83 | 1.869 | 11.52 | 0.985 |

**13**. **Iodine Release and Recyclability Study**

**Recyclability of Iodine Adsorption by Heating**:

To assess the recyclability of iodine adsorption, 10 mg of each coordination cage (**MC1**, **MC2**, **MC3**, and **MC4**) was placed separately in small vials. These vials were then enclosed in sealed containers containing solid iodine and heated to 75 °C under ambient pressure to facilitate I₂ adsorption. Once equilibrium was reached, the iodine-loaded cages (I₂@**MC1**, I₂@**MC2**, I₂@**MC3**, and I₂@**MC4**) were further heated to 110 °C to induce the release of adsorbed iodine. The recovered cage powders were subsequently reused for additional cycles of I₂ vapor adsorption.^[11]^

**Evaluation of Iodine Desorption Efficiency**:

The desorption efficiency of the iodine-loaded cages was analyzed using a gravimetric method. Pre-weighed samples of I₂-loaded cages were placed in small, sealed vials inside a larger evacuated chamber maintained at 110 °C under ambient pressure. The extent of iodine desorption was monitored at regular time intervals to evaluate the release efficiency.

**Iodine Release in Methanol Solution**:

For solution-phase iodine release studies, approximately 2.0 mg of each I₂-loaded cage (prepared via vapor-phase adsorption) was immersed in 3 mL of methanol. The release of iodine was monitored by measuring the time-dependent increase in absorbance at 445 nm using UV-Vis spectroscopy.

**Figure S117**. (a) UV-Vis spectra of iodine in methanol at various concentrations of I_2_ for standardization. (b) Linear fitting of absorbance versus concentration for I₂ in methanol.

**Figure S118**. (a) UV-Vis absorption spectra showing the release of I₂ from I₂@**MC1** in methanol at different time intervals. (b) Absorbance at 445 nm tracking I₂ release over various time points.

**Figure S119**. (a) Time-dependent UV-Vis absorption spectra of I₂ release from I₂@**MC2** in methanol. (b) Absorbance variation at 445 nm in methanol over different time intervals.

**Figure S120**. (a) UV-Vis absorption spectra showing the release of I₂ from I₂@**MC3** in methanol at different time intervals. (b) Absorbance at 445 nm tracking the I₂ release over time.

**Figure S121**. (a) Time-dependent UV-Vis absorption spectra showing the release of I₂ from I₂@**MC4** in methanol. (b) Absorbance changes at 445 nm over time in methanol.

**Figure S122**. Removal efficiency of I_2_ saturated cages (I_2_@**MC1,** I_2_**@MC2,** I_2_**@MC3** and I_2_**@MC4)** in methanol over time.

**Figure S123**. Time-dependent images of the I_2_ release process from adsorbed cages in methanol.

**Figure S124**. Pseudo-second-order kinetic model or I₂ removal from iodine-loaded cages: (a) I_2_@**MC1,** (b) **I_2_@MC2,** (c) **I_2_@MC3** and (d) **I_2_@MC4.**

**Table S12**. Iodine release kinetics in methanol

| Pseudo-second-order kinetics | **MC1** | **MC2** | **MC3** | **MC4** |
| --- | --- | --- | --- | --- |
| *k*_2_  (g mg^−1^ min^−1^) | 0.4888 | 0.8545 | 1.3256 | 0.5118 |

**Figure S125.** ^1^H NMR spectra of cage **MC1** before and after I_2_ adsorption from aqueous solution, and followed by subsequent I_2_ release (DMSO-*d*_6_, 700 MHz, 298K).

**Figure S126.** ^1^H NMR spectra of **MC2** prior to, after I_2_ loading in water, and followed by subsequent release of I_2_ (DMSO-*d*_6_, 700MHz, and 298 K).

**Figure S127.** ¹H NMR spectra of cage **MC3** before and after I_2_ adsorption from an aqueous solution, followed by subsequent I_2_ (DMSO-*d*_6_, 700 MHz, 298 K).

**Figure S128.** ^1^H NMR spectra of **MC4** prior to, after I_2_ loading in n-hexane, and following I_2_ release (DMSO-*d*_6_, 700MHz, and 298 K).

**Figure S129**. Iodine removal efficiency of recycled adsorbent **MC1** over five cycles.

**Figure S130**. Cyclic iodine adsorption and release efficiency of cage **MC3**.

**Figure S131**. Cyclic assessment of the reusability of **MC4** for I_2_ capture.

**14**. **Static Vapor-Phase Capture of Methyl Iodide**

The experimental setup for static CH_3_I capture was analogous to that employed for iodine vapor phase capture.^[18]^ Capture efficiency was determined using (Eq.1), and the kinetic data were found to be consistent with a pseudo-second-order kinetic model as described by (Eq.3).

**Figure S132**. Pseudo second order kinetics for methyl iodide capture by cages.

**Table S13**. Kinetics parameter for the methyl iodide adsorption

| Cage | Pseudo second order kinetics | | |
| --- | --- | --- | --- |
|  | *q*_e_ (mg g^−1^)  (calculated) | *k*_2_  (g g^−1^ h^−1^) | R^2^ |
| **MC1** | 1.112 | 1.4032 | 0.99 |
| **MC2** | 0.546 | 2.54 | 0.99 |
| **MC3** | 0.626 | 1.2 | 0.99 |
| **MC4** | 0.852 | 1.5117 | 0.99 |

**Figure S133**. CH_3_I retention efficiency of all four cages (**MC1**, **MC2**, **MC3** and **MC4**).

**Figure S134**. (a) Energy-dispersive X-ray spectroscopy data of the cage **MC1** after exposure to methyl iodide vapour. (b) Elemental mapping displaying the distribution of iodine within the adsorbed cage.

**Figure S135**. (a) Energy-dispersive X-ray spectroscopy data of the cage **MC2** following exposure to methyl iodide vapour. (b) Elemental mapping showing the distribution of iodine within the cage.

**Figure S136**. (a) EDAX of CH_3_I@**MC3**. (b) Elemental mapping showing the distribution of iodine within the cage.

**Figure S137**. (a) EDAX analysis of CH_3_I@**MC4**. (b) Elemental mapping showing the distribution of iodine within the cage.

**Figure S138**. In-situ FT-IR spectra of the cages recorded during methyl iodide adsorption.

**Figure S139**. Comparative analysis of N 1s binding energies in XPS spectra before and after methyl iodide adsorption for cages: (a) **MC2**, (b) **MC3** and (c) **MC4**.

**Figure S140**. XPS spectra of the I 3d region for methyl iodide-saturated cages: (a) CH_3_I@**MC2**, (b) CH_3_I@**MC3**, and (c) CH_3_I@**MC4.**

**Figure S141**. ^1^H NMR spectrum of the cage **MC1** after CH_3_I adsorption (DMSO-*d*_6_, 700 MHz, 298 K).

**Figure S142**. ^1^H NMR spectrum of **MC2** following exposure to CH_3_I vapour (DMSO-*d*_6_, 700 MHz, 298 K).

**Figure S143.** ^1^H NMR spectrum of **MC3** following CH_3_I adsorption (DMSO-*d*_6_, 700 MHz, 298 K).

**Figure S144**. ^1^H NMR spectrum of **MC4** after exposure in CH_3_I vapour (DMSO-*d*_6_, 700 MHz, 298 K).

**Figure S145**. ^13^C NMR spectra of cages (a) **MC1**, (b) **MC2**, (c) **MC3** and (d) **MC4** before and after exposure to CH_3_I vapour (DMSO-*d*_6_, 700 MHz, 298 K).

**15**. **DFT Calculations**

**Computational Details**.

All the structures were fully optimized^[19]^ using the B3-LYP functional with the triple-zeta valence basis set (def2TZVP) developed by Ahlrichs and co-workers^[20]^ using the Gaussian 16 program.^[21]^ Grimme’s D3 dispersion correction added to account for van der Waals (dispersion) interactions.^[22]^

**Figure S146**. Optimized structures of (a) **MC1**, (b) **MC2**, (c) **MC3** and (d) **MC4** (a representative fragment of the **MC4** cage was used due to its large molecular size), (Color code: C-grey, N-blue, H-white, Pd-green), along with the corresponding electrostatic potential (ESP) distributions.

**Figure S147**. DFT calculations of the binding energies (kcal·mol⁻¹) for **MC1** with I₂ at various binding sites, along with the corresponding electrostatic potential (ESP) distributions.

**Figure S148**. Calculations of binding energies of the I_3_⁻ ion interacting with **MC1** across various binding sites, along with the corresponding ESP.

**Figure S149**. DFT calculations of the binding energies for **MC2** with I_2_ at various binding sites, along with the ESP distributions.

**Figure S150**. The minimum binding energy site for the I_3_⁻ ion interacting with **MC2**, along with the corresponding ESP.

**Figure S151**. Calculations of the binding energies for **MC3** with I_2_ at various binding sites, along with the corresponding ESP distributions.

**Figure S152**. Binding energy calculated from DFT for the I_3_⁻ ion interacting with **MC3** across various binding sites, along with the corresponding ESP.

**Figure S153**. DFT calculation of the binding energy for **MC4** with I_2_, along with the corresponding ESP distribution.

**Figure S154**. The minimum binding energy site for the I_3_⁻ ion interacting with **MC4**, along with the corresponding ESP. (For **MC4**, a representative fragment of the cage was used due to its large molecular size).

**16**. **Comparative Study of Adsorbents for Vapor Phase Iodine Adsorption.**

**Table S14**. Comparison of iodine vapor adsorption capacities for various adsorbents

| Materials | Temperature (°C) | Uptake capacity (g g^−1^) | Materials type | Reference |
| --- | --- | --- | --- | --- |
| **MC3** | **75** | **3.38** | **MOC** | **This work** |
| C3 | 75 | 3.16 | MOC | *Inorg. Chem.* **2024**,  *63* (34), 15973– 15983 |
| cage-1 | 80 | 1.42 | MOC | *Chem. Commun*.  **2020**, *56*, 2491–2494 |
| Co-NDC-Br | 75 | 0.452 | MOC | *Cryst. Growth Des*.  **2022**, *22*, 3182 |
| Zn_12_L_4_ | 25 | 0.74 | MOC | *Langmuir* **2023**,  *39*(21), 7337-7344 |
| MOC19 | 75 | 0.33 | MOC | *Chem. Asian J*.  **2016**, *11*, 216 |
| 3.6Br | 75 | 5.89 | POC | *Nat. Commun*.  **2023**, *14*, 6082. |
| BPPOC | 75 | 5.64 | POC | *J. Am. Chem. Soc*. **2022**, *144*, 12390–12399 |
| OMC3 | 75 | 3.78 | POC | *Angew. Chemie - Int. Ed*.,**2020**, *59*, 20846–20851 |
| Bpy-cage | 75 | 3.23 | POC | *J. Am. Chem. Soc*.  **2022**, *144*, 113– 117 |
| BTPOC | 75 | 3.21 | POC | *J. Chem. Eng. J*.  **2022**, *428*, 131129 |
| CC3 | 20 | 0.45 | POC | *J. Am. Chem. Soc*. **2011**, *133*, 14920- 14923 |
| COF-OH-33 | 75 | 6.81 | COF | *Angew. Chem. Int. Ed*. **2021**, *60*, 22432–22440. |
| TPB-DMTP | 75 | 6.26 | COF | *Adv. Mater.* **2018**, *30*, 1801991 |
| Meso-COF-3 | 75 | 4.0 | COF | *Ind. Eng. Chem. Res*. **2019**, *58*, 10495 |
| QTD-COF-V | 75 | 6.29 | COF | *Angew. Chem., Int. Ed*. **2020**, 59, 22697 |
| ZIF-8 | 75 | 1.20 | MOF | *Ind. Eng. Chem. Res*. **2012**, *51*, 614 |
| PCN-333(Al) | 75 | 4.42 | MOF | *J. Mater. Chem. A*. **2019**, *7*, 18324–18329 |
| MOF-808 | 80 | 2.18 | MOF | ACS Appl. Mater. Interfaces **2020**, 12, 20429–20439 |
| CaIP4-Li | 75 | 3.18 | POP | *Chem. Mater*. **2017**, *29*, 8968–8972 |
| PAF-24 | 75 | 2.76 | POP | *Angew. Chem., Int. Ed*. **2015**, *54*, 12733 |

**17**. **Reference for Iodine Uptake in Aqueous Media**

**Table S15**. Comparison of iodine adsorption capacities in aqueous solutions across various materials

| Material | I_2_/ I_3_^−^ | Uptake capacity  (g g^−1^) | Materials  type | Reference |
| --- | --- | --- | --- | --- |
| SUPE-py-Imine-Cage | I_2_/KI | 9.01 | NAS | *Nat. Commun*.  **2023**, *14*, 5388. |
| IPcomp-7 | I_2_/KI | 5.16 | Hybrid composite | *Nat. Commun*.  **2024***,* *15*, 1278 |
| COFP–1 | I_2_ | 5.05 | COF | *Chem. Mater*.  **2022**, *34*, 11062–11071 |
| Impyr-HCl | I_2_/KI | 4.22 | COF | *Small*  **2024**, *20*, 2404994 |
| C[4]P-BTP | I_2_/KI | 3.24 | POP | *Angew. Chem. Int. Ed*.  **2022** *61*, e202113724 |
| HcOF-4 | I_2_/KI | 3.6 | HOF | *J. Am. Chem. Soc*.  **2019**, *141*, 10915-10923 |
| CaCOP3 | I_2_ | 3.1 | POP | *Mater. Chem. Phys*.  **2020**, *239*, 122328 |
| **MC2** | **I_2_** | **2.73** | **MOC** | **This work** |
| CalCOP1 | I_2_/KI | 2.32 | COP | *J. Mater. Sci*.  **2020**, *55*, 1854–1864 |
| **MC2** | **I_2_/KI** | **2.26** | **MOC** | **This work** |
| MeO–CTF600 | I_2_/KI | 2.21 | COF | ACS Appl. Nano Mater.  **2022**, *5*, 8783-8793 |
| HCOFs–1 | I_2_/KI | 2.1 | COF | *J. Am. Chem. Soc*.  **2017**, *139*, 7172-7175 |
| TIEPE-DABCO | I_2_/KI | 1.8 | XOF | Small  **2023**, *19*, 2302902 |
| BC@Dopa–ZIF | I_2_/KI | 1.31 | MOF | *Cryst. Growth Des*.  **2017**, *18*, 356-363 |
| 3D MOF-1 | I_2_/KI | 1.10 | MOF | *ACS Appl. Mater. Interfaces* **2020**, *12*, 46107-46118 |
| MBM-MOF | I_2_/KI | 0.88 | MOF | *Angew. Chem. Int. Ed*.  **2018**, *57*, 10148-10152 |
| G-QP6 | I_2_/KI | 0.247 | POP | *Angew. Chem. Int. Ed*.  **2019**, *58*, 3885 |
| Cd(II)- triazole MOF | I_2_/KI | 0.18 | MOF | *Chem. Commun*.  **2011**, *47*, 7185-7187 |

**18. Comparative Analysis of Adsorbent Recyclability for Vapor-Phase Iodine**

**Table S16**. A comparative analysis of iodine adsorption recyclability in the vapor phase across different materials

| Materials | Materials type | Regeneration method | Regeneration efficiency after the following cycles | No. of cycle | Reference |
| --- | --- | --- | --- | --- | --- |
| **MC2** | **MOC** | **Thermal desorption**  **(110 °C)** | **88%** | **5** | **This work** |
| C4 | MOC | Cyclohexane solution | 97.15 | 5 | *Inorg. Chem.*  **2024**, *63*, 15973– 15983 |
| TAPA-DKP COF | COF | Thermal desorption | 76% | 4 | *Small* **2025**, *21*, 2411199 |
| COF@EPNM | COF | Thermal desorption  (80 °C) | 68% | 4 | *Small* **2024**, 2409495 |
| ETTA-PyTTA-COF | COF | Methanol solution | 76.09 | 5 | *Chem. Res. Chin. Univ*. **2022**, *38*, 472–477. |
| Compound-1 and Compound-2 | POP | Methanolic solution of tetrabutyl  ammonium bromide | 80% | 3 | *ACS Appl. Mater. Interfaces* **2021**, *13*, 34188–34196 |
| (OFT-RTC 6+6Br⁻) | POC | Thermal desorption  (100 °C) | 95.4% | 5 | *Adv. Sci*. **2024**, *11*, 2408494 |
| TFB–Td COF | COF | Thermal desorption  (120 °C) | 35.41 | 5 | *ACS Appl. Mater. Interfaces* **2021**, *13*, 10513–10523 |
| TA-PDA COP | COP | Thermal desorption  (120 °C) | 97.2 % | 7 | *J. Hazard. Mater*. **2025**, 137753 |
| MOF-808 | MOF | Ethanol solution | 70% | 3 | *ACS Appl. Mater. Interfaces* **2020**, *12*, 20429–20439 |
| FV-1 | MOF | Ethanol solution | 74 | 4 | *CrystEngComm.* **2023**, 25, 2280–2297 |
| PD-POP | POP | Ethanol solution | 82.72 | 5 | *ACS Appl. Nano Mater.*  **2024**, *7*, 1797–1803 |
| C[4]PTPE | POP | Thermal desorption  (150 °C) | 86% | 5 | *Angew. Chem., Int. Ed.* **2022**, *61*, e202113724 |
| SCU-COF-2 | COF | Thermal desorption | 70% | 3 | *Chem* **2021**, *7*, 699–714, |

**19**. **Comparison of Rate Constant for I_3_^−^ Adsorption from Water**

**Table S17**. Adsorption kinetics of triiodide ions by various common adsorbents

| Materials | *k*_2_  (g mg^−1^ min^−1^) | Time (min) | Material type | Reference |
| --- | --- | --- | --- | --- |
| **MC2** | **4.25×10^−1^** | **4** | **MOC** | **This work** |
| **MC1** | **8.39×10^−2^** | **4** | **MOC** | **This work** |
| **MC3** | **5.97×10^−2^** | **4** | **MOC** | **This work** |
| OH-ExR4 | 1.18×10^−2^ | 27 | Supramolecular macrocycle | *Angew. Chem., Int. Ed*. **2024**, *63*, e202411261 |
| MOF nanosheets | 2.20×10^−3^ | 60 | MOF | *Inorg. Chem*.  **2022**, *61*, 13883–13892. |
| C[4]P-BTP | 7.81×10^−3^ | 10 | POP | *Angew. Chem., Int. Ed*.  **2022** *61*, e202113724 |
| LFC–K | 1.90×10^−3^ | 15 | ACM | *Ind. Crops Prod*.  **2021**, *169*, 113649 |
| PEG hydrogel | 1.50×10^−3^ | 270 | Others | *ACS Appl. Mater.*  *Interfaces* **2023**, *15*, 42942–42953 |
| TIEPE-DABCO | 6.7×10^−4^ | 300 | XOF | *Small*  **2023**, *19*, 2302902 |
| PTIBBL | 6.19×10^−4^ | 270 | POP | *Chem. Commun*.  **2020**, *56*, 1401–1404 |
| PP–3 | 5.90×10^−4^ | 30 | POP | J. Mater. Chem. A  **2022**, *10*, 20090–20100. |
| SUPE-py-Imine-Cage | 4.67×10^−4^ | 30 | NAS | *Nat. Commun*.  **2023**, *14*, 5388. |

**20**. **References**

[1] P. Karak, C. Dutta, T. Dutta, A. L. Koner, J. Choudhury, *Chem Commun.* **2019**, *55*, 6791-6794.

[2] Y. Zhang, S. Zhang, H. Wu, X. Dong, P. Shi, H. Qu, Y. Chen, X.-Y. Cao, Z.-Q. Tian, X. Hu, L. Yang, *J. Am. Chem. Soc.* **2022**, *144*, 19410-19416.

[3] C. Rizzo, F. D'Anna, R. Noto, M. Zhang, R. G. Weiss, *Chem. Eur. J.* **2016**, *22*, 11269-11282.

[4] P. Howlader, S. Mukherjee, R. Saha, P. S. Mukherjee, *Dalton Trans.* **2015**, *44*, 20493-20501.

[5] L. Yang, G. Chang, L. Luo, F. Ding, J. You, *Inorg. Chim. Acta* **2013**, *406*, 307-314.

[6] D. Samanta, P. S. Mukherjee, *Chem. Eur. J.* **2014**, *20*, 12483-12492.

[7] O. V. Dolomanov, L. J. Bourhis, R. J. Gildea, J. A. K. Howard, H. Puschmann, *J. Appl. Crystallogr.* **2009**, *42*, 339-341.

[8] (a) G. M. Sheldrick, *Acta Cryst. A* **2007**, *64*, 112-122; (b) G. M. Sheldrick, *Acta Cryst. A* **2015**, *71*, 3-8.

[9] F. Ren, Z. Zhu, X. Qian, W. Liang, P. Mu, H. Sun, J. Liu, A. Li, *Chem Commun.* **2016**, *52*, 9797-9800.

[10] S. Maji, R. Natarajan, *Small* **2023**, *19*, 2302902.

[11] Z. Zheng, Q. Lin, L. Xie, X. Chen, H. Zhou, K. Lin, D. Zhang, X. Chi, J. L. Sessler, H. Wang, *J. Mater. Chem. A* **2023**, *11*, 13399-13408.

[12] A. Sen, S. Sharma, S. Dutta, M. M. Shirolkar, G. K. Dam, S. Let, S. K. Ghosh, *ACS Appl. Mater. Interfaces* **2021**, *13*, 34188-34196.

[13] S. Fajal, W. Mandal, A. Torris, D. Majumder, S. Let, A. Sen, F. Kanheerampockil, M. M. Shirolkar, S. K. Ghosh, *Nat. Commun.* **2024**, *15*.

[14] J. L. Pursell, C. J. Pursell, *J. Phys. Chem. A.* **2016**, *120*, 2144-2149.

[15] L. Xie, Z. Zheng, Q. Lin, H. Zhou, X. Ji, J. L. Sessler, H. Wang, *Angew. Chem. Int. Ed.* **2021**, *61*, e202113724.

[16] (a) G. Matthys, A. Laemont, N. De Geyter, R. Morent, R. Lavendomme, P. Van Der Voort, *Small* **2024**, *20*, 2404994; (b) W. Zhou, A. Li, M. Zhou, Y. Xu, Y. Zhang, Q. He, *Nat. Commun.* **2023**, *14*, 5388.

[17] (a) M. Zhang, J. Samanta, B. A. Atterberry, R. Staples, A. J. Rossini, C. Ke, *Angew. Chem. Int. Ed.* **2022**, *61*, e202214189; (b) J. L. Lambert, G. L. Hatch, B. Mosier, *Anal. Chem.* **2002**, *47*, 915-916.

[18] (a) Y. Xie, T. Pan, Q. Lei, C. Chen, X. Dong, Y. Yuan, W. A. Maksoud, L. Zhao, L. Cavallo, I. Pinnau, Y. Han, *Nat. Commun.* **2022**, *13*, 2878; (b) S. Fajal, D. Majumder, W. Mandal, S. Let, G. K. Dam, M. M. Shirolkar, S. K. Ghosh, *J. Mater. Chem. A* **2023**, *11*, 26580-26591.

[19] (a) A. D. Becke, *J. Chem. Phys.* **1993**, *98*, 5648-5652; (b) C. Lee, W. Yang, R. G. Parr, *Phys. Rev. B* **1988**, *37*, 785-789.

[20] F. Weigend, R. Ahlrichs, *Phys. Chem. Chem. Phys.* **2005**, *7*.

[21] (a) M. J. Frisch, G. W. Trucks, H. B. Schlegel, G. E. Scuseria, M. A. Robb, J. R. Cheeseman, J. A. Montgomery Jr, T. Vreven, K. N. Kudin, J. C. Burant, J. M. Millam, S. S. Iyengar, J. Tomasi, V. Barone, B. Mennucci, M. Cossi, G. Scalmani, N. Rega, G. A. Petersson, H. Nakatsuji, M. Hada, M. Ehara, K. Toyota, R. Fukuda, J. Hasegawa, M. Ishida, T. Nakajima, Y. Honda, O. Kitao, H. Nakai, M. Klene, X. Li, J. E. Knox, H. P. Hratchian, J. B. Cross, C. Adamo, J. Jaramillo, R. Gomperts, R. E. Stratmann, O. Yazyev, A. J. Austin, R. Cammi, C. Pomelli, J. W. Ochterski, P. Y. Ayala, K. Morokuma, G. A. Voth, P. Salvador, J. J. Dannenberg, V. G. Zakrzewski, S. Dapprich, A. D. Daniels, M. C. Strain, O. Farkas, D. K. Malick, A. D. Rabuck, K. Raghavachari, J. B. Foresman, J. V. Ortiz, Q. Cui, A. G. Baboul, S. Clifford, J. Cioslowski, B. B. Stefanov, G. Liu, A. Liashenko, P. Piskorz, I. Komaromi, R. L. Martin, D. J. Fox, T. Keith, M. A. Al-Laham, C. Y. Peng, A. Nanayakkara, M. Challacombe, P. M. W. Gill, B. Johnson, W. Chen, M. W. Wong, C. Gonzalez and J. A. Pople, Gaussian 03, Revision E.01, Gaussian, Inc., Pittsburgh PA, **2003**. (b) M. J. Frisch, G. W. Trucks, H. B. Schlegel, G. E. Scuseria, M. A. Robb, J. R. Cheeseman, G. Scalmani, V. Barone, G. A. Petersson, H. Nakatsuji, X. Li, M. Caricato, A. V. Marenich, J. Bloino, B. G. Janesko, R. Gomperts, B. Mennucci, H. P. Hratchian, J. V. Ortiz, A. F. Izmaylov, J. L. Sonnenberg, D. Williams-Young, F. Ding, F. Lipparini, F. Egidi, J. Goings, B. Peng, A. Petrone, T. Henderson, D. Ranasinghe, V. G. Zakrzewski, J. Gao, N. Rega, G. Zheng, W. Liang, M. Hada, M. Ehara, K. Toyota, R. Fukuda, J. Hasegawa, M. Ishida, T. Nakajima, Y. Honda, O. Kitao, H. Nakai, T. Vreven, K. Throssell, J. A. Montgomery Jr, J. E. Peralta, F. Ogliaro, M. J. Bearpark, J. J. Heyd, E. N. Brothers, K. N. Kudin, V. N. Staroverov, T. A. Keith, R. Kobayashi, J. Normand, K. Raghavachari, A. P. Rendell, J. C. Burant, S. S. Iyengar, J. Tomasi, M.Cossi, J. M. Millam, M. Klene, C. Adamo, R. Cammi, J. W. Ochterski, R. L. Martin, K. Morokuma, O. Farkas, J. B. Foresman and D. J. Fox, Gaussian 16, Revision A.03, Gaussian, Inc., Wallingford CT, **2016.**

[22] S. Grimme, J. Antony, S. Ehrlich, H. Krieg, *J. Chem. Phys.* **2010**, *132*.
